# Big data insights into the diagnostic values of CBC parameters for sepsis and septic shock in burn patients: a retrospective study

**DOI:** 10.1038/s41598-023-50695-z

**Published:** 2024-01-08

**Authors:** Myongjin Kim, Dohern Kym, Jongsoo Park, Jaechul Yoon, Yong Suk Cho, Jun Hur, Wook Chun, Dogeon Yoon

**Affiliations:** 1 Department of Surgery and Critical Care, Burn Center, Hangang Sacred Heart Hospital, Hallym University Medical Center, 12, Beodeunaru-Ro 7-Gil, Youngdeungpo-Gu, 07247 Seoul, South Korea; 2 Burn Institutes, Hangang Sacred Heart Hospital, Hallym University Medical Center, 12, Beodeunaru-Ro 7-Gil, Youngdeungpo-Gu, 07247 Seoul, South Korea

**Keywords:** Medical research, Risk factors

## Abstract

Sepsis and septic shock are prevalent and life-threatening complications in burn patients. Despite their severity, existing diagnostic methods are limited. This study aims to evaluate the efficacy of Complete Blood Count (CBC) and CBC ratio markers in diagnosing sepsis and septic shock, and in predicting mortality among burn patients. A cohort of 2757 burn patients was examined to ascertain the correlation between various CBC parameters, their ratios, and the incidence of sepsis and related mortality. Key markers analyzed included Red Cell Distribution Width (RDW), Mean Platelet Volume (MPV), Neutrophil-to-Lymphocyte Ratio (NLR), Platelet-to-Lymphocyte Ratio (PLR), and Mean Platelet Volume-to-Platelet Ratio (MPVPR). Our findings indicate that 65.5% of the patients developed sepsis, and 24.3% succumbed to their conditions. The CBC parameters RDW, MPV, NLR, MPVPR, and MPV-to-Lymphocyte Ratio (MPVLR) were significantly associated with sepsis and mortality. These markers showed considerable temporal variation and yielded an Area Under the Curve (AUC) of over 0.65 in an unadjusted Generalized Estimating Equations (GEE) model. This study underscores the potential of RDW, MPV, NLR, MPVPR, and MPVLR as vital prognostic tools for diagnosing sepsis, septic shock, and predicting mortality in burn patients. Although based on a single-center dataset, our results contribute to the enhancement of sepsis management by facilitating earlier, more precise diagnosis and treatment strategies. Further multi-center research is necessary to confirm these findings and broaden their applicability, establishing a solid base for future explorations in this crucial field.

## Introduction

Sepsis, a critical condition caused by the body's extreme response to infection, is a common and serious complication among burn patients, leading to high mortality rates and a significant global health challenge. The early detection of sepsis is crucial to prevent adverse outcomes, yet diagnosis is often difficult due to the limitations of current biomarkers. These include moderate diagnostic and prognostic accuracy, lengthy turnaround times, and high costs^1^. In this context, Complete Blood Count (CBC) and CBC ratio markers have gained recognition as potentially valuable diagnostic tools. These markers, derived from routine, cost-effective, and widely available tests, could help clinicians promptly identify patients at high risk of developing sepsis and anticipate adverse outcomes^2^.

Recent research underscores the effectiveness of the Neutrophil-to-Lymphocyte Ratio (NLR), Platelet-to-Lymphocyte Ratio (PLR), and Monocyte-to-Lymphocyte Ratio (MLR) as biomarkers for early sepsis detection. Significantly altered in sepsis patients, these ratios offer independent, accessible, and affordable predictors, especially following procedures like percutaneous nephrolithotomy^3,4^. Furthermore, the Mean Platelet Volume (MPV) to Platelet count ratio, indicative of severe conditions, has been investigated as a potential biomarker that could improve diagnostic accuracy in conjunction with other clinical and laboratory findings^5^. Platelet count and related indices, including platelet dynamics, have also provided substantial prognostic insights into sepsis^6^. Among these CBC parameters, the Red Cell Distribution Width (RDW) has emerged as a reliable prognostic biomarker, correlating with disease severity and mortality^7^.

Given the high incidence and mortality rates of sepsis in burn patients, this study aims to evaluate the dual role of CBC and CBC ratio markers in burn-related injuries. We focus on their effectiveness as diagnostic tools for sepsis and septic shock, and their prognostic value in predicting mortality rates among burn victims. This research is vital for enhancing our understanding and use of these markers, potentially improving diagnostic capabilities and patient outcomes. It emphasizes the need for continued exploration and validation of these promising biomarkers in clinical settings.

## Material and methods

### Study setting and participant selection

**Figure 1:**
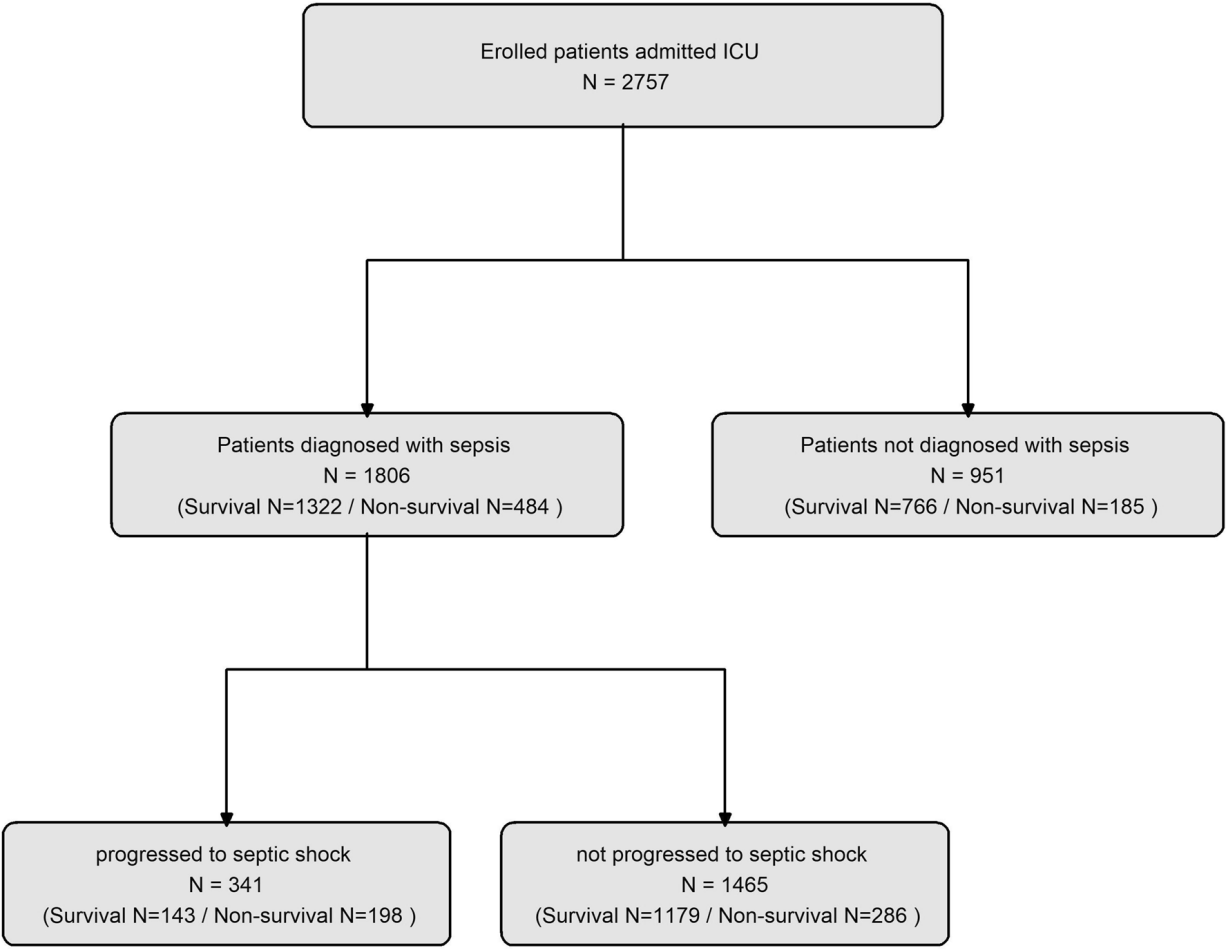
The flowchart of the enrollment process for patients included in this study.

This retrospective cohort study included 2757 adults (aged 18 and older) admitted to the Hangang Sacred Heart Hospital Burn Intensive Care Unit (ICU) for burn injuries from January 2010 to December 2022. The study imposed no exclusion criteria. We primarily investigated the incidence of sepsis in all patients, and a secondary analysis involved 1806 patients who developed sepsis, focusing on the occurrence of septic shock. A detailed patient participation flowchart is presented in Fig. 1.

### Data acquisition

Patient data were sourced from the Clinical Data Warehouse (CDW), a comprehensive system for storing medical data. This data encompassed essential patient details like age, sex, diagnosis, and ICU stay duration. Diagnoses of sepsis and septic shock were based on the Sepsis-3 criteria^8^, supported by our institution's research indicating its effectiveness in diagnosing burn patients^9^. The findings from the Surviving Sepsis After Burn (SSABC) guidelines in the 2023 Burns journal further corroborate this choice^10^. CBC parameters were collected daily throughout each patient's ICU stay, focusing particularly on the week preceding sepsis or septic shock onset. Key disease severity indicators such as APACHE IV^11^, SOFA score^12^, ABSI^13^, rBaux^14^, and Hangang Score^15^ were also gathered. The Hangang Score, specifically developed for burn patients, employs logistic regression coefficients for variables like age, total body surface area burned (% TBSA), inhalation injury presence, and levels of lactate, pH, prothrombin time, serum bilirubin, myoglobin, creatinine, and lactate dehydrogenase. Each variable contributes to a cumulative score estimating mortality risk.

The study's primary outcome was the incidence of sepsis and septic shock, while the secondary outcome focused on the 60-day in-hospital mortality rate. The study approved and the informed consent was waived due to retrospective nature by the Institutional Review Board (IRB) of Hangang Sacred Heart Hospital.

#### Definition of ratio markers

In this study, several ratio markers were defined as follows:

1. Neutrophil-to-Lymphocyte Ratio (NLR): Absolute Neutrophil Count/Absolute Lymphocyte Count.
  The NLR measures the balance between neutrophils and lymphocytes in the blood. Elevated NLR values are associated with poor outcomes^16^.
2. Platelet-to-Lymphocyte Ratio (PLR): Platelet Count/Lymphocyte Count.
  The PLR represents the ratio of platelet count to lymphocyte count. High PLR values indicate a high level of systemic inflammation and are indicative of a worse prognosis for the disease^17^.
3. Monocyte-to-Lymphocyte Ratio (MLR): Monocyte Count/Lymphocyte Count.
  The MLR is calculated by dividing the monocyte count by the lymphocyte count. Increased MLR values can be associated with poor outcomes in various diseases, including cancer^18^.
4. Systemic Immune-Inflammation Index (SII): Platelet Count × Neutrophil Count/Lymphocyte Count.
  The SII is an index that incorporates platelet count, neutrophil count, and lymphocyte count. It has been associated with poor outcomes in various diseases, including cancer^19^.
  Additionally, the following ratios have been suggested in some studies, although they are not standard markers: Refs.^20,21^.
5. MPV-to-Platelet Ratio (MPVPR) : MPV/Platelet Count.
6. MPV-to-Lymphocyte Ratio (MPVLR): MPV/Lymphocyte Count.
7. MPV-to-Monocyte Ratio (MPVMR): MPV/Monocyte Count.
8. MPV-to-Neutrophil Ratio (MPVNR): MPV/Neutrophil Count.

### Statistical analysis

Continuous variables following a normal distribution were presented as means ± standard deviation (SD), while those not normally distributed were shown as medians with interquartile range (IQR). Categorical variables were expressed as percentages. Statistical analysis involved independent t-tests for normally distributed continuous variables, Mann–Whitney U-tests for non-normally distributed variables, and the Chi-square test for categorical variables. To limit the effect of outliers, Winsorization was applied.

Predictors were scaled due to unit differences, and Generalized Estimating Equations (GEE) were used to analyze temporal changes and predict sepsis and septic shock with unbalanced repeated data. GEE estimates population-averaged effects and is suitable for unbalanced repeated measures data. An autoregressive order 1 (AR1) structure was included in the GEE model to account for correlations in repeated measures.

The predictive value of the GEE model was assessed using Area Under the Curve (AUC) analysis, along with accuracy, sensitivity, specificity, positive predictive value (PPV), and negative predictive value (NPV). For mortality prediction, the survival distribution was compared using the log-rank test, and hazard ratios (HR) were calculated using the Cox proportional hazards model. Adjustments were made for age, TBSA, and inhalation injuries, significant predictors in burn patients. All statistical tests were two-tailed, with a p-value of less than 0.05 considered significant. Analyses were conducted using the R-project program version 4.3.0.

### Ethics approval and consent to participate

Conducted in alignment with the Declaration of Helsinki, this study was approved and informed consent was waived due to its retrospective nature by the Institutional Review Board (IRB) of Hangang Sacred Heart Hospital (HG2 2023-003).

## Results

### Characteristics and prediction of sepsis and mortality in enrolled patients

In this study, out of the 2757 patients, 1806 (65.5%) developed sepsis, while 669 (24.3%) experienced mortality. Gender differences were not statistically significant in relation to sepsis or mortality outcomes. All CBC parameters, except eosinophils, MCH, PLR, MPVLR, and MPVMR, showed significant differences related to sepsis and mortality outcomes (Table 1). The odds ratios for age, TBSA, and inflammation, crucial prognostic factors in burn patients, are presented in Table S1, both adjusted and unadjusted.

**Table 1:** Characteristics of Enrolled Patients by Sepsis and Mortality.

Group	Variables	Sepsis				Mortality		
		Overall, N = 2,757	Yes, N = 1806 (65.5%)	No, N = 951 (34.5%)	p-value	Yes, N = 669 (24.3%)	No, N = 2088 (75.7%)	p-value
Demographics	Age							
	Median [IQR]	52 [41, 63]	52 [42, 63]	50 [39, 61]	0.003	56 [47, 70]	50 [40, 60]	< 0.001
	Sex							
	Male	2180 (79.1%)	1418 (78.5%)	762 (80.1%)	0.349	520 (77.7%)	1660 (79.5%)	0.326
	Female	577 (20.9%)	388 (21.5%)	189 (19.9%)		149 (22.3%)	428 (20.5%)	
	Type							
	FB	1918 (69.6%)	1340 (74.2%)	578 (60.9%)	< 0.001	578 (86.4%)	1340 (64.2%)	< 0.001
	SB	275 (10.0%)	173 (9.6%)	102 (10.7%)		48 (7.2%)	227 (10.9%)	
	EB	369 (13.4%)	168 (9.3%)	201 (21.2%)		16 (2.4%)	353 (16.9%)	
	ChB	46 (1.7%)	26 (1.4%)	20 (2.1%)		5 (0.7%)	41 (2.0%)	
	CoB	147 (5.3%)	99 (5.5%)	48 (5.1%)		22 (3.3%)	125 (6.0%)	
	TBSA							
	Median [IQR]	27 [15, 46]	33 [20, 51]	20 [10, 30]	< 0.001	61 [40, 84]	22 [12, 34]	< 0.001
	Inhalation	1,063 (38.6%)	757 (41.9%)	306 (32.2%)		399 (59.6%)	664 (31.8%)	
	LOS							
	Median [IQR]	12 [5, 27]	21 [10, 35]	4 [2, 6]	< 0.001	11 [4, 21]	12 [5, 30]	< 0.001
Severity scores	ABSI							
	Median [IQR]	8 [6, 10]	8 [7, 10]	6 [5, 8]	< 0.001	12 [9, 14]	7 [6, 8]	< 0.001
	rBaux							
	Median [IQR]	88 [70, 110]	95 [78, 114]	74 [60, 94]	< 0.001	129 [108, 148]	81 [65, 95]	< 0.001
	Hangang							
	Median [IQR]	131 [119, 147]	135 [124, 149]	121 [113, 136]	< 0.001	162 [149, 178]	125 [116, 135]	< 0.001
	APACHE_IV							
	Median [IQR]	40 [24, 62]	44 [29, 63]	30 [18, 60]	< 0.001	66 [49, 91]	33 [21, 51]	< 0.001
	SOFA							
	Median [IQR]	3 [2, 5]	3 [2, 5]	2 [1, 4]	< 0.001	5 [3, 8]	3 [1, 4]	< 0.001
WBC related	WBC							
	Median [IQR]	16.9 [11.8, 23.8]	18.3 [12.6, 24.1]	14.7 [10.9, 20.2]	< 0.001	24.1 [17.5, 24.1]	15.3 [11.1, 20.5]	< 0.001
	Neutrophil							
	Median [IQR]	13.9 [9.1, 19.8]	15.2 [9.9, 20.3]	12.0 [8.0, 16.8]	< 0.001	19.7 [12.9, 20.3]	12.8 [8.6, 17.6]	< 0.001
	Lymphocyte							
	Median [IQR]	1.56 [1.07, 2.40]	1.54 [1.02, 2.34]	1.60 [1.11, 2.46]	0.017	2.14 [1.23, 2.74]	1.48 [1.03, 2.10]	< 0.001
	Monocyte							
	Median [IQR]	0.85 [0.58, 1.26]	0.94 [0.61, 1.35]	0.70 [0.50, 1.05]	< 0.001	1.20 [0.67, 1.37]	0.80 [0.55, 1.12]	< 0.001
	Eosinophil							
	Median [IQR]	0.10 [0.04, 0.20]	0.10 [0.04, 0.19]	0.10 [0.05, 0.20]	0.041	0.10 [0.05, 0.20]	0.10 [0.04, 0.19]	0.192
	Basophil							
	Median [IQR]	0.06 [0.03, 0.10]	0.06 [0.03, 0.10]	0.05 [0.03, 0.10]	< 0.001	0.09 [0.05, 0.13]	0.05 [0.03, 0.09]	< 0.001
	Immature Granulocyte							
	Median [IQR]	0.11 [0.06, 0.25]	0.14 [0.07, 0.29]	0.07 [0.04, 0.17]	< 0.001	0.28 [0.15, 1.05]	0.09 [0.05, 0.17]	< 0.001
RBC related	RBC							
	Median [IQR]	4.91 [4.30, 5.19]	4.99 [4.23, 5.19]	4.81 [4.34, 5.19]	0.020	5.19 [4.36, 5.19]	4.83 [4.29, 5.19]	< 0.001
	RDW							
	Median [IQR]	12.93 [12.30, 13.90]	13.09 [12.40, 14.03]	12.70 [12.17, 13.64]	< 0.001	14.03 [13.10, 15.70]	12.70 [12.20, 13.40]	< 0.001
	Hct							
	Median [IQR]	45.6 [40.0, 46.2]	46.2 [39.7, 46.2]	44.3 [40.2, 46.2]	0.003	46.2 [42.3, 46.2]	44.8 [39.8, 46.2]	< 0.001
	Hb							
	Median [IQR]	15.30 [13.40, 16.20]	15.60 [13.30, 16.40]	15.00 [13.50, 16.00]	< 0.001	16.00 [13.50, 16.50]	15.10 [13.40, 16.20]	< 0.001
	MCV							
	Median [IQR]	92.0 [89.0, 95.3]	92.1 [89.1, 95.4]	91.8 [88.9, 94.8]	0.149	93.2 [90.1, 96.7]	91.7 [88.8, 94.7]	< 0.001
	MCH							
	Median [IQR]	31.20 [30.10, 32.30]	31.20 [30.10, 32.30]	31.10 [30.10, 32.10]	0.025	31.00 [29.90, 32.40]	31.20 [30.10, 32.30]	0.199
	MCHC							
	Median [IQR]	34.00 [33.20, 34.70]	34.10 [33.30, 34.80]	33.90 [33.20, 34.70]	0.039	33.70 [32.58, 34.50]	34.10 [33.40, 34.80]	< 0.001
Platelet related	Platelet							
	Median [IQR]	254 [197, 322]	255 [192, 327]	251 [205, 311]	0.291	288 [201, 397]	247 [196, 305]	< 0.001
	MPV							
	Median [IQR]	9.80 [9.14, 10.54]	9.94 [9.34, 10.64]	9.50 [8.94, 10.20]	< 0.001	10.19 [9.14, 11.20]	9.76 [9.14, 10.34]	< 0.001
	PDW							
	Median [IQR]	10.90 [9.78, 12.50]	11.05 [9.91, 12.70]	10.50 [9.50, 11.90]	< 0.001	11.80 [10.01, 14.20]	10.70 [9.70, 12.00]	< 0.001
	PCT							
	Median [IQR]	0.20 [0.20, 0.30]	0.20 [0.20, 0.30]	0.20 [0.20, 0.27]	0.243	0.23 [0.20, 0.35]	0.20 [0.19, 0.27]	< 0.001
Ratios	NLR							
	Median [IQR]	11 [7, 17]	12 [7, 18]	8 [5, 14]	< 0.001	12 [7, 19]	10 [6, 16]	< 0.001
	PLR							
	Median [IQR]	189 [129, 273]	199 [131, 288]	178 [127, 247]	< 0.001	190 [123, 271]	189 [132, 273]	0.180
	MLR							
	Median [IQR]	0.66 [0.42, 1.00]	0.73 [0.48, 1.09]	0.52 [0.34, 0.79]	< 0.001	0.75 [0.47, 1.14]	0.64 [0.41, 0.95]	< 0.001
	SII							
	Median [IQR]	2,475 [1,379, 4,157]	2,736 [1,528, 4,465]	2,073 [1,086, 3,550]	< 0.001	2,898 [1,733, 4,771]	2,362 [1,279, 3,941]	< 0.001
	MPVPR							
	Median [IQR]	0.043 [0.033, 0.060]	0.045 [0.034, 0.065]	0.041 [0.032, 0.053]	< 0.001	0.049 [0.032, 0.076]	0.043 [0.033, 0.055]	< 0.001
	MPVLR							
	Median [IQR]	8.1 [5.5, 11.8]	8.9 [5.8, 12.9]	7.1 [4.9, 10.1]	< 0.001	8.2 [4.7, 13.0]	8.1 [5.7, 11.6]	0.483
	MPVMR							
	Median [IQR]	14 [10, 21]	13 [9, 20]	15 [11, 21]	< 0.001	13 [8, 23]	14 [10, 20]	0.086
	MPVNR							
	Median [IQR]	0.88 [0.60, 1.40]	0.83 [0.57, 1.37]	0.93 [0.66, 1.45]	< 0.001	0.72 [0.54, 1.24]	0.90 [0.63, 1.43]	< 0.001

**Figure 2:**
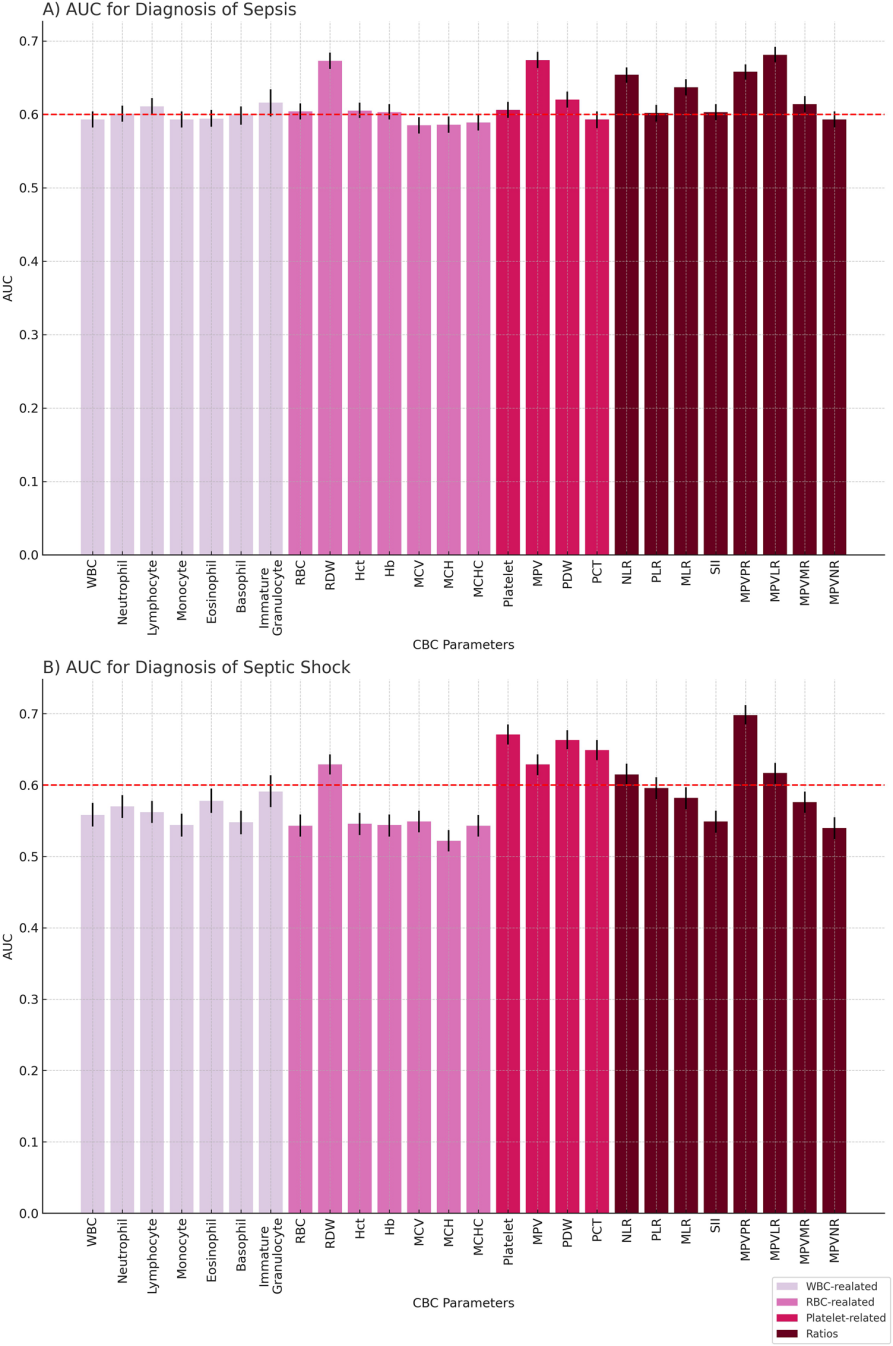
Comparative AUC Bar Graphs for CBC Parameters (**A**) in the Diagnosis of Sepsis, (**B**) in the Diagnosis of Septic Shock.

Using the unadjusted GEE model, AUC analysis identified markers with an AUC over 0.65: RDW (AUC = 0.673), MPV (AUC = 0.674), NLR (AUC = 0.654), MPVPR (AUC = 0.658), and MPVLR (AUC = 0.681), as shown in Fig. 2A. Other diagnostic performance metrics, such as accuracy, sensitivity, specificity, PPV, and NPV, are detailed in Table 2. For mortality prediction, RDW, MPV, PDW, and MPVPR exhibited HR and adjusted HR above 1 in all sepsis patients (Table 3). Temporal changes of all markers in relation to the presence or absence of sepsis are depicted in Fig. S1.

**Table 2:** Performance of Generalized Estimating Equations (GEE) Model in Diagnosis Sepsis: AUC Analysis.

CBC parameters	Variables	AUC (95%CI)	Accuracy (95%CI)	Sensitivity (95%CI)	Specificity (95%CI)	PPV (95%CI)	NPV (95%CI)
WBC-related	WBC	0.593 (0.582 ~ 0.604)	0.556 (0.547 ~ 0.565)	0.538 (0.528 ~ 0.549)	0.603 (0.586 ~ 0.619)	0.785 (0.774 ~ 0.795)	0.327 (0.315 ~ 0.339)
	Neutrophil	0.601 (0.590 ~ 0.612)	0.558 (0.549 ~ 0.566)	0.540 (0.529 ~ 0.550)	0.606 (0.589 ~ 0.623)	0.787 (0.777 ~ 0.797)	0.328 (0.316 ~ 0.340)
	Lymphocyte	0.611 (0.600 ~ 0.622)	0.565 (0.556 ~ 0.574)	0.545 (0.534 ~ 0.555)	0.620 (0.603 ~ 0.636)	0.794 (0.784 ~ 0.804)	0.336 (0.324 ~ 0.348)
	Monocyte	0.593 (0.582 ~ 0.604)	0.559 (0.550 ~ 0.568)	0.541 (0.530 ~ 0.551)	0.608 (0.591 ~ 0.625)	0.788 (0.778 ~ 0.798)	0.329 (0.318 ~ 0.341)
	Eosinophil	0.594 (0.583 ~ 0.606)	0.567 (0.558 ~ 0.577)	0.560 (0.549 ~ 0.571)	0.588 (0.570 ~ 0.605)	0.784 (0.773 ~ 0.794)	0.333 (0.320 ~ 0.346)
	Basophil	0.599 (0.586 ~ 0.611)	0.564 (0.554 ~ 0.573)	0.551 (0.540 ~ 0.562)	0.602 (0.583 ~ 0.621)	0.804 (0.793 ~ 0.815)	0.311 (0.298 ~ 0.324)
	Immature Granulocyte	0.616 (0.597 ~ 0.634)	0.564 (0.550 ~ 0.578)	0.548 (0.531 ~ 0.564)	0.611 (0.583 ~ 0.638)	0.801 (0.784 ~ 0.817)	0.321 (0.302 ~ 0.341)
RBC-related	RBC	0.604 (0.593 ~ 0.615)	0.570 (0.561 ~ 0.579)	0.548 (0.538 ~ 0.559)	0.629 (0.613 ~ 0.646)	0.799 (0.789 ~ 0.809)	0.341 (0.329 ~ 0.353)
	RDW	0.673 (0.662 ~ 0.684)	0.607 (0.598 ~ 0.616)	0.573 (0.563 ~ 0.584)	0.697 (0.681 ~ 0.712)	0.836 (0.826 ~ 0.845)	0.378 (0.366 ~ 0.390)
	Hct	0.605 (0.595 ~ 0.616)	0.570 (0.561 ~ 0.578)	0.548 (0.538 ~ 0.558)	0.627 (0.611 ~ 0.644)	0.798 (0.788 ~ 0.808)	0.341 (0.329 ~ 0.353)
	Hb	0.603 (0.593 ~ 0.614)	0.573 (0.565 ~ 0.582)	0.550 (0.540 ~ 0.561)	0.635 (0.618 ~ 0.651)	0.802 (0.792 ~ 0.812)	0.344 (0.333 ~ 0.356)
	MCV	0.585 (0.574 ~ 0.596)	0.554 (0.545 ~ 0.562)	0.537 (0.526 ~ 0.547)	0.599 (0.582 ~ 0.615)	0.782 (0.772 ~ 0.793)	0.325 (0.313 ~ 0.336)
	MCH	0.586 (0.575 ~ 0.597)	0.551 (0.542 ~ 0.560)	0.535 (0.525 ~ 0.546)	0.594 (0.577 ~ 0.610)	0.780 (0.769 ~ 0.790)	0.322 (0.310 ~ 0.334)
	MCHC	0.589 (0.578 ~ 0.600)	0.558 (0.550 ~ 0.567)	0.542 (0.531 ~ 0.552)	0.603 (0.587 ~ 0.620)	0.786 (0.776 ~ 0.796)	0.329 (0.317 ~ 0.341)
Platelet-related	Platelet	0.606 (0.595 ~ 0.617)	0.563 (0.554 ~ 0.572)	0.543 (0.533 ~ 0.553)	0.616 (0.599 ~ 0.632)	0.792 (0.781 ~ 0.802)	0.334 (0.322 ~ 0.346)
	MPV	0.674 (0.663 ~ 0.685)	0.604 (0.595 ~ 0.613)	0.572 (0.562 ~ 0.582)	0.690 (0.674 ~ 0.706)	0.833 (0.823 ~ 0.842)	0.375 (0.362 ~ 0.387)
	PDW	0.620 (0.609 ~ 0.631)	0.571 (0.562 ~ 0.580)	0.549 (0.539 ~ 0.559)	0.631 (0.614 ~ 0.647)	0.800 (0.790 ~ 0.810)	0.342 (0.330 ~ 0.354)
	PCT	0.593 (0.581 ~ 0.604)	0.563 (0.554 ~ 0.572)	0.543 (0.533 ~ 0.554)	0.616 (0.599 ~ 0.633)	0.792 (0.781 ~ 0.802)	0.334 (0.322 ~ 0.346)
Ratios	NLR	0.654 (0.643 ~ 0.664)	0.586 (0.577 ~ 0.595)	0.559 (0.549 ~ 0.569)	0.658 (0.642 ~ 0.675)	0.815 (0.805 ~ 0.825)	0.356 (0.344 ~ 0.369)
	PLR	0.602 (0.590 ~ 0.613)	0.563 (0.554 ~ 0.572)	0.543 (0.533 ~ 0.554)	0.617 (0.600 ~ 0.633)	0.792 (0.782 ~ 0.803)	0.334 (0.322 ~ 0.346)
	MLR	0.637 (0.626 ~ 0.648)	0.577 (0.568 ~ 0.586)	0.553 (0.543 ~ 0.563)	0.642 (0.625 ~ 0.658)	0.806 (0.796 ~ 0.816)	0.348 (0.336 ~ 0.360)
	SII	0.603 (0.592 ~ 0.614)	0.561 (0.552 ~ 0.570)	0.546 (0.535 ~ 0.556)	0.603 (0.586 ~ 0.619)	0.787 (0.777 ~ 0.797)	0.330 (0.318 ~ 0.342)
	MPVPR	0.658 (0.647 ~ 0.668)	0.593 (0.584 ~ 0.602)	0.564 (0.553 ~ 0.574)	0.671 (0.655 ~ 0.687)	0.822 (0.812 ~ 0.831)	0.364 (0.352 ~ 0.376)
	MPVLR	0.681 (0.671 ~ 0.692)	0.604 (0.595 ~ 0.612)	0.571 (0.561 ~ 0.581)	0.692 (0.676 ~ 0.707)	0.833 (0.824 ~ 0.843)	0.374 (0.362 ~ 0.386)
	MPVMR	0.614 (0.603 ~ 0.625)	0.574 (0.565 ~ 0.582)	0.550 (0.540 ~ 0.561)	0.636 (0.619 ~ 0.653)	0.803 (0.793 ~ 0.813)	0.344 (0.332 ~ 0.356)
	MPVNR	0.593 (0.582 ~ 0.604)	0.564 (0.555 ~ 0.573)	0.544 (0.533 ~ 0.554)	0.618 (0.601 ~ 0.635)	0.794 (0.783 ~ 0.804)	0.334 (0.322 ~ 0.346)

**Table 3:** HR and Adjusted HR for Mortality using GEE Model in Enrolled Patients.

CBC parameters	Variables	Hazard Ratio (95%CI)	p-value	adjusted Hazard Ratio (95%CI)	p- value	Log-Lank test p-value
WBC-related	WBC	0.886 (0.823 ~ 0.954)	0.001*	0.905 (0.841 ~ 0.974)	0.008*	0.001*
	Neutrophil	0.944 (0.876 ~ 1.017)	0.127	0.950 (0.882 ~ 1.024)	0.179	0.124
	Lymphocyte	0.931 (0.864 ~ 1.003)	0.061	0.808 (0.750 ~ 0.871)	< 0.001**	0.059
	Monocyte	0.838 (0.778 ~ 0.903)	< 0.001**	0.851 (0.789 ~ 0.917)	< 0.001**	< 0.001**
	Eosinophil	0.905 (0.836 ~ 0.981)	0.015*	0.820 (0.757 ~ 0.888)	< 0.001**	0.014*
	Basophil	0.886 (0.817 ~ 0.961)	0.004*	0.867 (0.799 ~ 0.941)	< 0.001**	0.003*
	Immature Granulocyte	1.051 (0.932 ~ 1.185)	0.415	1.001 (0.888 ~ 1.129)	0.984	0.416
RBC-related	RBC	0.781 (0.726 ~ 0.841)	< 0.001**	0.854 (0.793 ~ 0.919)	< 0.001**	< 0.001**
	RDW	1.921 (1.765 ~ 2.091)	< 0.001**	1.371 (1.259 ~ 1.493)	< 0.001**	< 0.001**
	Hct	0.730 (0.678 ~ 0.785)	< 0.001**	0.799 (0.743 ~ 0.861)	< 0.001**	< 0.001**
	Hb	0.786 (0.730 ~ 0.846)	< 0.001**	0.840 (0.780 ~ 0.905)	< 0.001**	< 0.001**
	MCV	0.816 (0.759 ~ 0.879)	< 0.001**	0.801 (0.745 ~ 0.863)	< 0.001**	< 0.001**
	MCH	0.766 (0.711 ~ 0.824)	< 0.001**	0.779 (0.724 ~ 0.839)	< 0.001**	< 0.001**
	MCHC	0.715 (0.665 ~ 0.770)	< 0.001**	0.749 (0.695 ~ 0.806)	< 0.001**	< 0.001**
Platelet-related	Platelet	1.275 (1.183 ~ 1.375)	< 0.001**	0.945 (0.877 ~ 1.020)	0.145	< 0.001**
	MPV	1.809 (1.669 ~ 1.962)	< 0.001**	1.225 (1.129 ~ 1.329)	< 0.001**	< 0.001**
	PDW	1.669 (1.542 ~ 1.806)	< 0.001**	1.164 (1.075 ~ 1.261)	< 0.001**	< 0.001**
	PCT	0.914 (0.847 ~ 0.986)	0.019*	0.809 (0.750 ~ 0.873)	< 0.001**	0.019*
Ratios	NLR	1.326 (1.226 ~ 1.434)	< 0.001**	1.015 (0.937 ~ 1.098)	0.720	< 0.001**
	PLR	0.634 (0.589 ~ 0.684)	< 0.001**	0.703 (0.652 ~ 0.758)	< 0.001**	< 0.001**
	MLR	0.984 (0.913 ~ 1.060)	0.669	0.931 (0.864 ~ 1.004)	0.064	0.660
	SII	0.724 (0.672 ~ 0.780)	< 0.001**	0.807 (0.749 ~ 0.870)	< 0.001**	< 0.001**
	MPVPR	1.866 (1.720 ~ 2.024)	< 0.001**	1.159 (1.066 ~ 1.259)	< 0.001**	< 0.001**
	MPVLR	1.491 (1.375 ~ 1.617)	< 0.001**	0.964 (0.888 ~ 1.046)	0.377	< 0.001**
	MPVMR	1.271 (1.176 ~ 1.374)	< 0.001**	0.911 (0.842 ~ 0.985)	0.020*	< 0.001**
	MPVNR	0.905 (0.840 ~ 0.976)	0.010*	0.804 (0.746 ~ 0.867)	< 0.001**	0.009*

** indicates a *p*-value < 0.001, and * signifies a *p*-value < 0.05.

### Characteristics and prediction of septic shock and mortality in sepsis patients

Among the 1806 patients with sepsis, 341 (18.9%) developed septic shock, and 484 (26.8%) succumbed to mortality. Gender differences were not statistically significant for septic shock or mortality outcomes. Most CBC parameters, except for inhalation injury, eosinophil count, Hb, MCV, MCH, MCHC, Platelet count, PCT, SII, MPVLR, and MPVMR, demonstrated significant differences related to septic shock and mortality (Table S2).

RDW and SII were statistically significant in all temporal changes, adjusted or not, for diagnosing septic shock (Table S3). The odds ratio for septic shock diagnosis is detailed in Table S3. For diagnosing septic shock, markers with AUC above 0.65 were platelet count (AUC = 0.671), PDW (AUC = 0.663), and MPVPR (AUC = 0.698), as shown in Fig. 2B. Diagnostic performance metrics such as accuracy, sensitivity, specificity, PPV, and NPV are included in Table 4. For mortality prediction, all except monocyte count and SII showed statistical differences, and both HR and adjusted HR for the risk group were above 1 (Table 5). Temporal changes in all markers relative to the presence or absence of septic shock are illustrated in Fig. S2.

**Table 4:** Performance of Generalized Estimating Equations (GEE) Model in Diganosis Septic Shock: AUC Analysis.

CBC parameters	Variables	AUC (95%CI)	Accuracy (95%CI)	Sensitivity (95%CI)	Specificity (95%CI)	PPV (95%CI)	NPV (95%CI)
WBC-related	WBC	0.558 (0.542 ~ 0.575)	0.525 (0.515 ~ 0.535)	0.569 (0.545 ~ 0.593)	0.515 (0.504 ~ 0.527)	0.205 (0.194 ~ 0.217)	0.845 (0.834 ~ 0.855)
	Neutrophil	0.570 (0.554 ~ 0.586)	0.527 (0.516 ~ 0.537)	0.575 (0.550 ~ 0.599)	0.516 (0.505 ~ 0.528)	0.208 (0.196 ~ 0.220)	0.846 (0.835 ~ 0.857)
	Lymphocyte	0.562 (0.547 ~ 0.578)	0.526 (0.515 ~ 0.536)	0.572 (0.548 ~ 0.596)	0.516 (0.504 ~ 0.527)	0.207 (0.195 ~ 0.219)	0.845 (0.834 ~ 0.856)
	Monocyte	0.544 (0.528 ~ 0.560)	0.515 (0.505 ~ 0.525)	0.543 (0.518 ~ 0.567)	0.509 (0.497 ~ 0.520)	0.196 (0.185 ~ 0.208)	0.835 (0.823 ~ 0.845)
	Eosinophil	0.578 (0.561 ~ 0.595)	0.526 (0.515 ~ 0.537)	0.600 (0.573 ~ 0.626)	0.511 (0.499 ~ 0.523)	0.202 (0.190 ~ 0.215)	0.861 (0.850 ~ 0.871)
	Basophil	0.548 (0.531 ~ 0.564)	0.515 (0.503 ~ 0.526)	0.554 (0.527 ~ 0.580)	0.506 (0.494 ~ 0.519)	0.197 (0.185 ~ 0.210)	0.838 (0.826 ~ 0.850)
	Immature Granulocyte	0.591 (0.569 ~ 0.614)	0.534 (0.518 ~ 0.551)	0.576 (0.541 ~ 0.611)	0.522 (0.503 ~ 0.541)	0.260 (0.240 ~ 0.281)	0.809 (0.790 ~ 0.827)
RBC-related	RBC	0.543 (0.528 ~ 0.559)	0.513 (0.503 ~ 0.523)	0.536 (0.512 ~ 0.561)	0.508 (0.496 ~ 0.519)	0.193 (0.182 ~ 0.205)	0.833 (0.821 ~ 0.843)
	RDW	0.629 (0.615 ~ 0.643)	0.550 (0.540 ~ 0.560)	0.648 (0.624 ~ 0.671)	0.529 (0.517 ~ 0.540)	0.232 (0.220 ~ 0.245)	0.872 (0.862 ~ 0.882)
	Hct	0.546 (0.530 ~ 0.561)	0.515 (0.504 ~ 0.525)	0.541 (0.516 ~ 0.565)	0.509 (0.497 ~ 0.520)	0.195 (0.184 ~ 0.207)	0.834 (0.823 ~ 0.845)
	Hb	0.544 (0.528 ~ 0.559)	0.513 (0.503 ~ 0.523)	0.539 (0.515 ~ 0.564)	0.507 (0.496 ~ 0.519)	0.194 (0.183 ~ 0.206)	0.833 (0.822 ~ 0.844)
	MCV	0.549 (0.534 ~ 0.564)	0.516 (0.506 ~ 0.527)	0.550 (0.525 ~ 0.574)	0.509 (0.498 ~ 0.520)	0.198 (0.186 ~ 0.210)	0.837 (0.826 ~ 0.848)
	MCH	0.522 (0.507 ~ 0.537)	0.507 (0.496 ~ 0.517)	0.524 (0.499 ~ 0.548)	0.503 (0.491 ~ 0.514)	0.188 (0.177 ~ 0.200)	0.828 (0.816 ~ 0.838)
	MCHC	0.543 (0.528 ~ 0.558)	0.519 (0.509 ~ 0.529)	0.553 (0.529 ~ 0.578)	0.511 (0.500 ~ 0.523)	0.199 (0.188 ~ 0.211)	0.839 (0.828 ~ 0.849)
Platelet-related	Platelet	0.671 (0.657 ~ 0.685)	0.563 (0.553 ~ 0.573)	0.675 (0.652 ~ 0.698)	0.538 (0.527 ~ 0.550)	0.244 (0.231 ~ 0.256)	0.883 (0.873 ~ 0.892)
	MPV	0.629 (0.614 ~ 0.643)	0.550 (0.540 ~ 0.561)	0.649 (0.626 ~ 0.673)	0.529 (0.517 ~ 0.540)	0.232 (0.220 ~ 0.245)	0.873 (0.863 ~ 0.882)
	PDW	0.663 (0.650 ~ 0.677)	0.577 (0.566 ~ 0.587)	0.713 (0.690 ~ 0.735)	0.547 (0.535 ~ 0.558)	0.257 (0.244 ~ 0.270)	0.897 (0.887 ~ 0.905)
	PCT	0.649 (0.635 ~ 0.663)	0.548 (0.538 ~ 0.559)	0.687 (0.663 ~ 0.709)	0.518 (0.507 ~ 0.530)	0.236 (0.224 ~ 0.249)	0.884 (0.874 ~ 0.893)
Ratios	NLR	0.615 (0.600 ~ 0.630)	0.548 (0.538 ~ 0.559)	0.634 (0.610 ~ 0.657)	0.530 (0.518 ~ 0.541)	0.229 (0.217 ~ 0.242)	0.868 (0.857 ~ 0.877)
	PLR	0.596 (0.580 ~ 0.611)	0.537 (0.526 ~ 0.547)	0.601 (0.577 ~ 0.625)	0.522 (0.511 ~ 0.534)	0.217 (0.205 ~ 0.230)	0.856 (0.845 ~ 0.866)
	MLR	0.582 (0.566 ~ 0.597)	0.535 (0.524 ~ 0.545)	0.596 (0.571 ~ 0.620)	0.521 (0.510 ~ 0.533)	0.215 (0.203 ~ 0.228)	0.854 (0.843 ~ 0.864)
	SII	0.549 (0.533 ~ 0.564)	0.516 (0.506 ~ 0.527)	0.545 (0.521 ~ 0.570)	0.510 (0.499 ~ 0.522)	0.197 (0.186 ~ 0.209)	0.836 (0.825 ~ 0.846)
	MPVPR	0.698 (0.685 ~ 0.712)	0.583 (0.573 ~ 0.594)	0.731 (0.709 ~ 0.753)	0.551 (0.539 ~ 0.562)	0.263 (0.251 ~ 0.277)	0.903 (0.894 ~ 0.912)
	MPVLR	0.617 (0.602 ~ 0.631)	0.547 (0.537 ~ 0.557)	0.631 (0.606 ~ 0.654)	0.529 (0.517 ~ 0.540)	0.228 (0.215 ~ 0.240)	0.867 (0.856 ~ 0.877)
	MPVMR	0.576 (0.561 ~ 0.591)	0.530 (0.520 ~ 0.541)	0.584 (0.559 ~ 0.608)	0.518 (0.507 ~ 0.530)	0.211 (0.199 ~ 0.223)	0.850 (0.839 ~ 0.860)
	MPVNR	0.540 (0.524 ~ 0.555)	0.520 (0.509 ~ 0.530)	0.554 (0.529 ~ 0.579)	0.512 (0.500 ~ 0.523)	0.200 (0.188 ~ 0.212)	0.839 (0.828 ~ 0.850)

**Table 5:** HR and Adjusted HR for Mortality using GEE Model in Sepsis Patients.

CBC parameters	Variables	Hazard Ratio (95%CI)	p- value	adjusted Hazard Ratio (95%CI)	p-value	Log-Lank test p-value
WBC-related	WBC	1.146 (1.057 ~ 1.243)	< 0.001**	1.216 (1.121 ~ 1.319)	< 0.001**	< 0.001**
	Neutrophil	1.224 (1.127 ~ 1.329)	< 0.001**	1.243 (1.145 ~ 1.349)	< 0.001**	< 0.001**
	Lymphocyte	1.396 (1.285 ~ 1.516)	< 0.001**	1.238 (1.140 ~ 1.345)	< 0.001**	< 0.001**
	Monocyte	1.024 (0.943 ~ 1.111)	0.577	1.135 (1.045 ~ 1.231)	0.002*	0.570
	Eosinophil	1.329 (1.217 ~ 1.451)	< 0.001**	1.167 (1.068 ~ 1.274)	< 0.001**	< 0.001**
	Basophil	1.212 (1.108 ~ 1.325)	< 0.001**	1.165 (1.065 ~ 1.274)	< 0.001**	< 0.001**
	Immature Granulocyte	1.300 (1.140 ~ 1.483)	< 0.001**	1.209 (1.059 ~ 1.380)	0.005*	< 0.001**
RBC-related	RBC	1.185 (1.093 ~ 1.285)	< 0.001**	1.117 (1.030 ~ 1.211)	0.008*	< 0.001**
	RDW	2.736 (2.486 ~ 3.010)	< 0.001**	1.961 (1.780 ~ 2.161)	< 0.001**	< 0.001**
	Hct	1.207 (1.113 ~ 1.309)	< 0.001**	1.099 (1.014 ~ 1.192)	0.022*	< 0.001**
	Hb	1.157 (1.067 ~ 1.254)	< 0.001**	1.101 (1.015 ~ 1.194)	0.020*	< 0.001**
	MCV	1.534 (1.413 ~ 1.666)	< 0.001**	1.353 (1.245 ~ 1.471)	< 0.001**	< 0.001**
	MCH	1.142 (1.053 ~ 1.238)	0.001*	1.106 (1.020 ~ 1.200)	0.014*	0.001*
	MCHC	1.308 (1.205 ~ 1.419)	< 0.001**	1.192 (1.099 ~ 1.294)	< 0.001**	< 0.001**
Platelet-related	Platelet	2.512 (2.296 ~ 2.749)	< 0.001**	1.639 (1.495 ~ 1.797)	< 0.001**	< 0.001**
	MPV	2.421 (2.213 ~ 2.649)	< 0.001**	1.726 (1.576 ~ 1.891)	< 0.001**	< 0.001**
	PDW	2.147 (1.967 ~ 2.344)	< 0.001**	1.475 (1.348 ~ 1.613)	< 0.001**	< 0.001**
	PCT	2.343 (2.136 ~ 2.569)	< 0.001**	1.504 (1.367 ~ 1.654)	< 0.001**	< 0.001**
Ratios	NLR	1.670 (1.534 ~ 1.819)	< 0.001**	1.406 (1.290 ~ 1.532)	< 0.001**	< 0.001**
	PLR	1.423 (1.311 ~ 1.545)	< 0.001**	1.292 (1.189 ~ 1.403)	< 0.001**	< 0.001**
	MLR	1.211 (1.115 ~ 1.315)	< 0.001**	1.182 (1.088 ~ 1.283)	< 0.001**	< 0.001**
	SII	1.073 (0.989 ~ 1.164)	0.092	1.118 (1.030 ~ 1.213)	0.007*	0.090
	MPVPR	3.338 (3.031 ~ 3.677)	< 0.001**	2.129 (1.927 ~ 2.352)	< 0.001**	< 0.001**
	MPVLR	1.989 (1.822 ~ 2.171)	< 0.001**	1.416 (1.294 ~ 1.548)	< 0.001**	< 0.001**
	MPVMR	1.787 (1.641 ~ 1.946)	< 0.001**	1.324 (1.214 ~ 1.444)	< 0.001**	< 0.001**
	MPVNR	1.117 (1.029 ~ 1.212)	0.008*	1.166 (1.074 ~ 1.266)	< 0.001**	0.008*

** indicates a *p*-value < 0.001, and * signifies a *p*-value < 0.05.

## Discussions

The present study delved into exploring sepsis and septic shock in burn patients, focusing on various Complete Blood Count (CBC) parameters as prognostic markers. Our key findings included identifying significant markers with an Area Under the Curve (AUC) exceeding 0.65, such as RDW, MPV, NLR, MPVPR, and MPVLR for sepsis and platelet count, PDW, and MPVPR for septic shock. Notably, RDW, MPV, PDW, and MPVPR showed Hazard Ratios (HR) and adjusted HRs above 1 in all sepsis patients, suggesting their potential in predicting mortality.

RDW, a marker we found meaningful, has been linked to mortality in septic burn patients. A meta-analysis of 17,961 sepsis patients across eleven studies revealed that higher baseline RDW correlated with increased mortality, consistent across different subgroups. This indicates RDW's potential as a reliable predictor of mortality in sepsis, with higher values suggesting greater risk^7,22^.

Additionally, the neutrophil-to-lymphocyte ratio (NLR) showed prognostic value in sepsis. A separate meta-analysis involving 11,564 patients found that non-surviving sepsis patients had notably higher NLRs than survivors, suggesting its usefulness as a biomarker for sepsis prognosis, with higher values indicating poorer outcomes^23^.

​In our research, MPV-related ratios, particularly MPVPR and MPVLR, emerged as significant markers in diagnosing sepsis, evidenced by an Area Under the Curve (AUC) exceeding 0.65. This aligns with previous studies suggesting the effectiveness of these ratios in various diseases^20,21^. Their broad applicability in different clinical contexts underscores their significance beyond our study's scope. While not yet standard in clinical practice, their potential is evident in numerous studies exploring diverse pathologies. Our study adds to the evidence on the clinical relevance of these ratios, which reflect the interplay between platelet and lymphocyte counts. These ratios are particularly insightful for understanding the immune-inflammatory response in burn infections, considering the roles of platelets in inflammation and lymphocytes in adaptive immunity. However, factors like patient age, TBSA burned, and overall inflammation status, all pivotal in burn patient prognosis, may influence these markers' effectiveness.

Clinically, our findings could expedite diagnosing and starting treatment for sepsis and septic shock in burn patients, a notable challenge due to pathophysiological similarities between large burns and sepsis. Identifying several CBC parameters as potential sepsis, septic shock, and mortality predictors could refine prognostic models and inform treatment approaches. The temporal variations of these markers, as we discovered, suggest that dynamic CBC parameter changes could offer vital insights into patient outcomes. Addressing the high sepsis prevalence in our study is crucial. Our patient cohort, mainly severe burn patients, inherently has a higher sepsis risk due to extensive tissue damage and inflammatory response^24^. Additionally, using Sepsis-3 criteria, which focuses on organ failure as defined by the SOFA score, might have contributed to a higher sepsis incidence in our group. This inclusive diagnostic approach could account for the observed increase in sepsis rates. We've included culture-positive test site data in Fig. S3 to further clarify sepsis diagnoses in our study, providing detailed information on the infection sources among our patients. These considerations highlight the importance of taking into account patient characteristics, diagnostic criteria, and microbiological data when assessing sepsis prevalence in burn patients^25^.

This study marks a significant advancement in medical literature by thoroughly examining a broad spectrum of Complete Blood Count (CBC) parameters in an extensive cohort of burn patients. However, it's important to note its limitations, particularly its single-center design, which may limit the generalizability of the findings to other environments or demographics. Despite accounting for several major confounders, the possibility of residual confounding from unmeasured variables remains. The utility of a diagnostic test goes beyond its Area Under the Curve (AUC) value, encompassing the clinical context of its application. In this research, the CBC markers are proposed as adjuncts to existing diagnostic protocols, rather than standalone tools. This is especially pertinent for markers with moderate AUC values, where integrating them with clinical judgment and other diagnostic criteria could reduce the risk of sepsis underdiagnosis. The pre-test probability of sepsis in a specific patient population should also be considered; markers with moderate AUC values can significantly enhance patient outcomes through early diagnosis and timely intervention in high-risk groups. Future research should explore these markers' applications in various clinical settings and in prospective studies to affirm their role as predictive tools. Integrating these markers into clinical practice should be carefully considered within the full diagnostic process, taking into account the available clinical pathways and resources. This balanced approach ensures these markers improve diagnostic accuracy for sepsis and septic shock without missing potential cases. Overall, this study lays the groundwork for further exploration into the prognostic capabilities of CBC parameters in burn care.

## Conclusion

This research underscores the importance of specific CBC parameters, including RDW, MPV, NLR, MPVPR, and MPVLR, as diagnostic and prognostic tools for sepsis, septic shock, and mortality in burn patients. These markers could significantly enhance the current practices in sepsis management, contributing to improved patient outcomes by allowing for earlier and more accurate diagnosis and treatment. Despite being a single-center study, our findings lay the groundwork for further research. Future studies should aim to validate these markers across diverse settings and populations, potentially through multi-center or prospective studies. Additionally, further investigation into other potential markers could also provide new insights. As we continue to refine our understanding of these markers, we can work towards more personalized and effective treatment strategies for sepsis and septic shock in burn patients.

### Supplementary Information

Supplementary Information 1.

Supplementary Information 2.

## Data Availability

The datasets used and/or analyzed during the current study are available from the corresponding author upon reasonable request.
