# Supplementary material for: Big data insights into the diagnostic values of CBC parameters for sepsis and septic shock in burn patients: a retrospective study
Source: Sci Rep. 2024 Jan 8;14:800. doi: 10.1038/s41598-023-50695-z (PMC10774327; doi:10.1038/s41598-023-50695-z)

**Supplementary material**

**Big Data Insights into the Diagnostic Values of CBC Parameters for Sepsis and Septic Shock in Burn Patients: A Retrospective Study**

Myongjin Kim1, Dohern Kym,1,2*, Jongsoo Park1, Jaechul Yoon1, Yong Suk Cho1,2, Jun Hur1,2, Wook Chun1,2, Dogeon Yoon2

1 Department of Surgery and Critical Care, Burn Center, Hangang Sacred Heart Hospital, Hallym University Medical Center, 12, Beodeunaru-ro 7-gil, Youngdeungpo-gu, Seoul, Korea, 07247

2 Burn Institutes, Hangang Sacred Heart Hospital, Hallym University Medical Center, 12, Beodeunaru-ro 7-gil, Youngdeungpo-gu, Seoul, Korea, 07247

+ These authors contributed this word equally as a corresponding author.

*Corresponding authors

Department of Surgery and Critical Care, Burn Center, Hangang Sacred Heart Hospital, College of Medicine, Hallym University 12, Beodeunaru-ro 7-gil, Youngdeungpo-gu, Seoul, Korea, 07247

Tel. 82-2-2639-5446, Fax. 82-2-2678-4386, E-mail: dohern@hallym.or.kr,hammerj@hallym.or.kr

Contents

[Table S1. Odds Ratios for Sepsis Diagnosis Using Generalized Estimating Equations Model 5](#_Toc152615603)

[Figure S1. Temporal changes of all markers according to the presence or absence of sepsis. 7](#_Toc152615604)

[a) WBC 7](#_Toc152615605)

[b) Neutrophil 8](#_Toc152615606)

[c) Lymphocyte 9](#_Toc152615607)

[d) Monocyte 10](#_Toc152615608)

[e) Basophil 11](#_Toc152615609)

[f) Immature Granulocyte 12](#_Toc152615610)

[g) RBC 13](#_Toc152615611)

[h) RDW 14](#_Toc152615612)

[i) Hct 15](#_Toc152615613)

[j) Hb 16](#_Toc152615614)

[k) MCV 17](#_Toc152615615)

[l) MCH 18](#_Toc152615616)

[m) MCHC 19](#_Toc152615617)

[n) Platelet 20](#_Toc152615618)

[o) MPV 21](#_Toc152615619)

[p) PDW 22](#_Toc152615620)

[q) PCT 23](#_Toc152615621)

[r) NLR 24](#_Toc152615622)

[s) PLR 25](#_Toc152615623)

[t) MLR 26](#_Toc152615624)

[u) SII 27](#_Toc152615625)

[v) MPVPR 28](#_Toc152615626)

[x) MPVLR 29](#_Toc152615627)

[y) MPVMR 30](#_Toc152615628)

[z) MPVNR 31](#_Toc152615629)

[Table S2. Characteristics of Sepsis Patients by Shock and Mortality 32](#_Toc152615630)

[Table S3. Odds Ratios for Septic Shock Diagnosis Using Generalized Estimating Equations Model 37](#_Toc152615631)

[Figure S2. Temporal changes in all markers relative to the presence or absence of septic shock. 39](#_Toc152615632)

[a) WBC 39](#_Toc152615633)

[b) Neutrophil 40](#_Toc152615634)

[c) Lymphocyte 41](#_Toc152615635)

[d) Monocyte 42](#_Toc152615636)

[e) Basophil 43](#_Toc152615637)

[f) Immature Granulocyte 44](#_Toc152615638)

[g) RBC 45](#_Toc152615639)

[h) RDW 46](#_Toc152615640)

[i) Hct 47](#_Toc152615641)

[j) Hb 48](#_Toc152615642)

[k) MCV 49](#_Toc152615643)

[l) MCH 50](#_Toc152615644)

[m) MCHC 51](#_Toc152615645)

[n) Platelet 52](#_Toc152615646)

[o) MPV 53](#_Toc152615647)

[p) PDW 54](#_Toc152615648)

[q) PCT 55](#_Toc152615649)

[r) NLR 56](#_Toc152615650)

[s) PLR 57](#_Toc152615651)

[t) MLR 58](#_Toc152615652)

[u) SII 59](#_Toc152615653)

[v) MPVPR 60](#_Toc152615654)

[x) MPVLR 61](#_Toc152615655)

[y) MPVMR 62](#_Toc152615656)

[z) MPVNR 63](#_Toc152615657)

[Figure S3. Culture-Positivity Frequency during Sepsis Diagnosis 64](#_Toc152615658)

# Table S1. Odds Ratios for Sepsis Diagnosis Using Generalized Estimating Equations Model

| CBC parameters | Variables | Odd Ratio (95%CI) | p-value | Odd ratio over time (95%CI) | p-value | adjusted Odd Ratio (95%CI) | p-value | adjusted Odd ratio over time (95%CI) | p-value |
| --- | --- | --- | --- | --- | --- | --- | --- | --- | --- |
| WBC-realated | WBC | 1.004 (1.000 - 1.008) | 0.069 | 1.002 (1.001 - 1.003) | 0.001 * | 0.993 (0.987 - 0.999) | 0.017 * | 1.005 (1.003 - 1.006) | <0.001 ** |
|  | Neutrophil | 1.006 (1.002 - 1.010) | 0.006 * | 1.002 (1.001 - 1.003) | <0.001 ** | 0.998 (0.992 - 1.003) | 0.437 | 1.005 (1.003 - 1.006) | <0.001 ** |
|  | Lymphocyte | 0.992 (0.988 - 0.996) | <0.001 ** | 0.998 (0.997 - 1.000) | 0.004 * | 0.980 (0.974 - 0.985) | <0.001 ** | 1.001 (0.999 - 1.002) | 0.271 |
|  | Monocyte | 1.016 (1.011 - 1.020) | <0.001 ** | 0.996 (0.995 - 0.997) | <0.001 ** | 1.011 (1.005 - 1.018) | <0.001 ** | 0.998 (0.996 - 0.999) | 0.002 * |
|  | Eosinophil | 0.997 (0.991 - 1.002) | 0.224 | 0.999 (0.998 - 1.001) | 0.253 | 0.995 (0.987 - 1.003) | 0.243 | 1.000 (0.998 - 1.002) | 0.901 |
|  | Basophil | 1.002 (0.995 - 1.009) | 0.574 | 1.002 (1.000 - 1.003) | 0.026 * | 0.986 (0.977 - 0.995) | 0.003 * | 1.005 (1.002 - 1.007) | <0.001 ** |
|  | Immature Granulocyte | 1.014 (1.003 - 1.024) | 0.010 * | 1.000 (0.998 - 1.003) | 0.683 | 1.001 (0.987 - 1.015) | 0.876 | 1.003 (1.000 - 1.006) | 0.068 |
| RBC-realated | RBC | 0.993 (0.987 - 0.999) | 0.019 * | 0.996 (0.995 - 0.997) | <0.001 ** | 0.981 (0.972 - 0.990) | <0.001 ** | 0.997 (0.995 - 0.999) | 0.002 * |
|  | RDW | 1.040 (1.023 - 1.057) | <0.001 ** | 1.008 (1.006 - 1.010) | <0.001 ** | 1.010 (0.989 - 1.032) | 0.349 | 1.012 (1.009 - 1.015) | <0.001 ** |
|  | Hct | 0.991 (0.985 - 0.996) | 0.001 * | 0.998 (0.996 - 0.999) | <0.001 ** | 0.978 (0.970 - 0.986) | <0.001 ** | 0.999 (0.997 - 1.001) | 0.193 |
|  | Hb | 0.995 (0.990 - 1.001) | 0.106 | 0.995 (0.994 - 0.996) | <0.001 ** | 0.984 (0.976 - 0.993) | <0.001 ** | 0.996 (0.994 - 0.997) | <0.001 ** |
|  | MCV | 1.005 (0.993 - 1.016) | 0.425 | 0.999 (0.997 - 1.000) | 0.106 | 0.993 (0.978 - 1.009) | 0.420 | 0.998 (0.996 - 1.000) | 0.066 |
|  | MCH | 1.012 (1.002 - 1.022) | 0.020 * | 0.997 (0.996 - 0.999) | <0.001 ** | 1.019 (1.004 - 1.034) | 0.015 * | 0.996 (0.994 - 0.998) | <0.001 ** |
|  | MCHC | 1.009 (1.003 - 1.015) | 0.002 * | 0.999 (0.998 - 1.000) | 0.038 * | 1.019 (1.010 - 1.028) | <0.001 ** | 0.998 (0.996 - 1.000) | 0.049 * |
| Platelet-realated | Platelet | 0.990 (0.984 - 0.996) | <0.001 ** | 0.998 (0.997 - 1.000) | 0.024 * | 0.980 (0.970 - 0.990) | <0.001 ** | 1.001 (0.999 - 1.003) | 0.268 |
|  | MPV | 1.039 (1.029 - 1.049) | <0.001 ** | 1.001 (0.999 - 1.002) | 0.514 | 1.044 (1.029 - 1.060) | <0.001 ** | 1.000 (0.998 - 1.003) | 0.804 |
|  | PDW | 1.012 (1.007 - 1.017) | <0.001 ** | 1.000 (0.999 - 1.001) | 0.466 | 1.011 (1.003 - 1.018) | 0.005 * | 1.000 (0.998 - 1.001) | 0.558 |
|  | PCT | 0.998 (0.992 - 1.003) | 0.407 | 0.999 (0.998 - 1.000) | 0.092 | 0.988 (0.978 - 0.999) | 0.033 * | 1.001 (0.999 - 1.004) | 0.170 |
| Ratios | NLR | 1.015 (1.010 - 1.019) | <0.001 ** | 1.002 (1.001 - 1.003) | <0.001 ** | 1.018 (1.012 - 1.024) | <0.001 ** | 1.002 (1.001 - 1.004) | 0.002 * |
|  | PLR | 1.013 (1.008 - 1.018) | <0.001 ** | 1.000 (0.999 - 1.001) | 0.652 | 1.019 (1.012 - 1.027) | <0.001 ** | 1.000 (0.998 - 1.001) | 0.702 |
|  | MLR | 1.024 (1.019 - 1.028) | <0.001 ** | 0.998 (0.997 - 0.999) | <0.001 ** | 1.030 (1.024 - 1.037) | <0.001 ** | 0.997 (0.996 - 0.999) | <0.001 ** |
|  | SII | 1.007 (1.003 - 1.011) | <0.001 ** | 1.002 (1.001 - 1.003) | <0.001 ** | 1.006 (1.000 - 1.012) | 0.054 | 1.003 (1.002 - 1.005) | <0.001 ** |
|  | MPVPR | 1.023 (1.017 - 1.029) | <0.001 ** | 1.002 (1.000 - 1.003) | 0.019 * | 1.027 (1.016 - 1.037) | <0.001 ** | 1.000 (0.998 - 1.002) | 0.962 |
|  | MPVLR | 1.027 (1.021 - 1.033) | <0.001 ** | 1.001 (0.999 - 1.002) | 0.359 | 1.040 (1.031 - 1.048) | <0.001 ** | 0.998 (0.997 - 1.000) | 0.097 |
|  | MPVMR | 1.002 (0.997 - 1.007) | 0.476 | 1.003 (1.002 - 1.004) | <0.001 ** | 1.001 (0.994 - 1.009) | 0.741 | 1.003 (1.001 - 1.005) | 0.003 * |
|  | MPVNR | 1.010 (1.006 - 1.015) | <0.001 ** | 0.998 (0.996 - 0.999) | <0.001 ** | 1.016 (1.009 - 1.023) | <0.001 ** | 0.996 (0.994 - 0.997) | <0.001 ** |

** This is p-value < 0.001.; * This is p-value < 0.05

# Figure S1. Temporal changes of all markers according to the presence or absence of sepsis.

## a) WBC


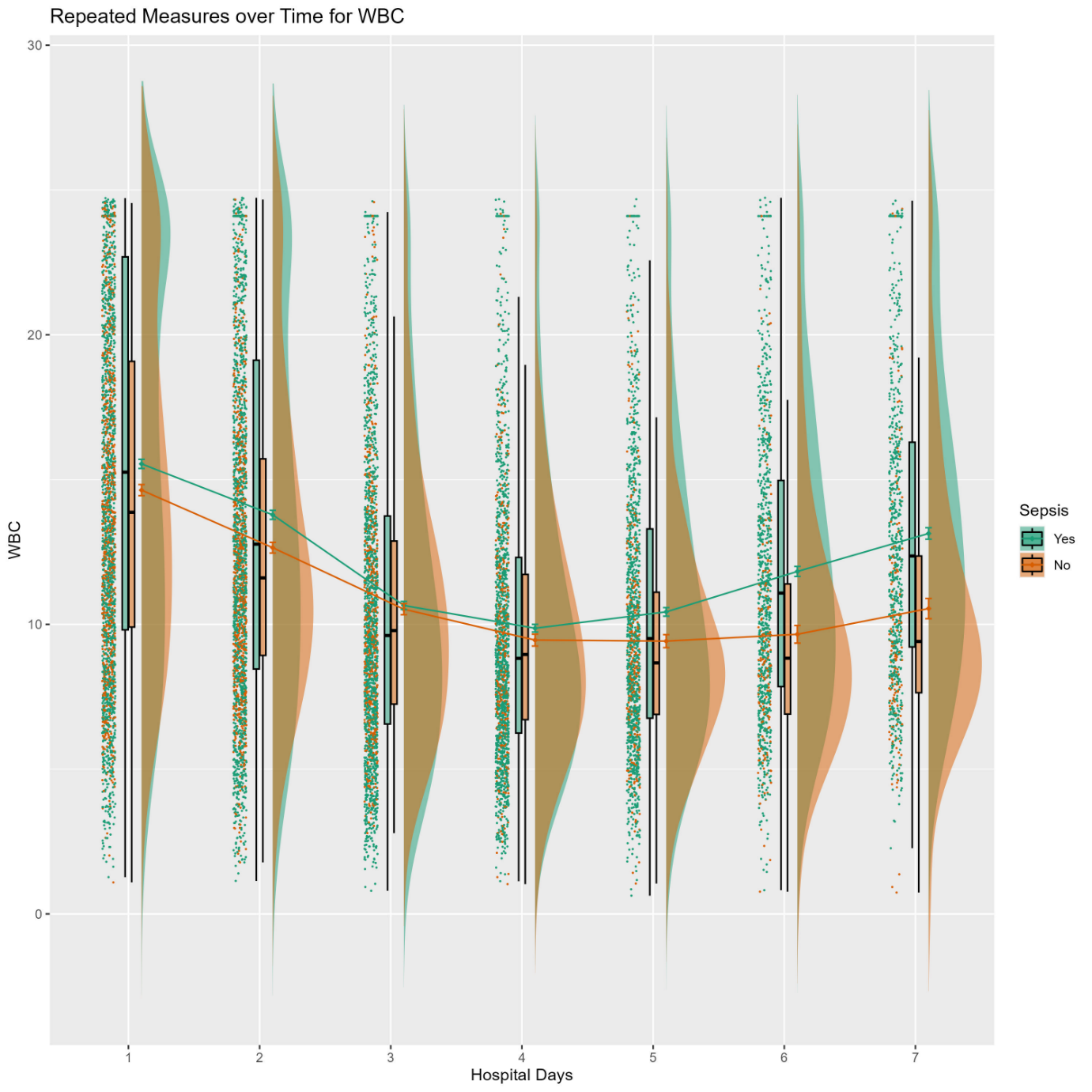


## b) Neutrophil


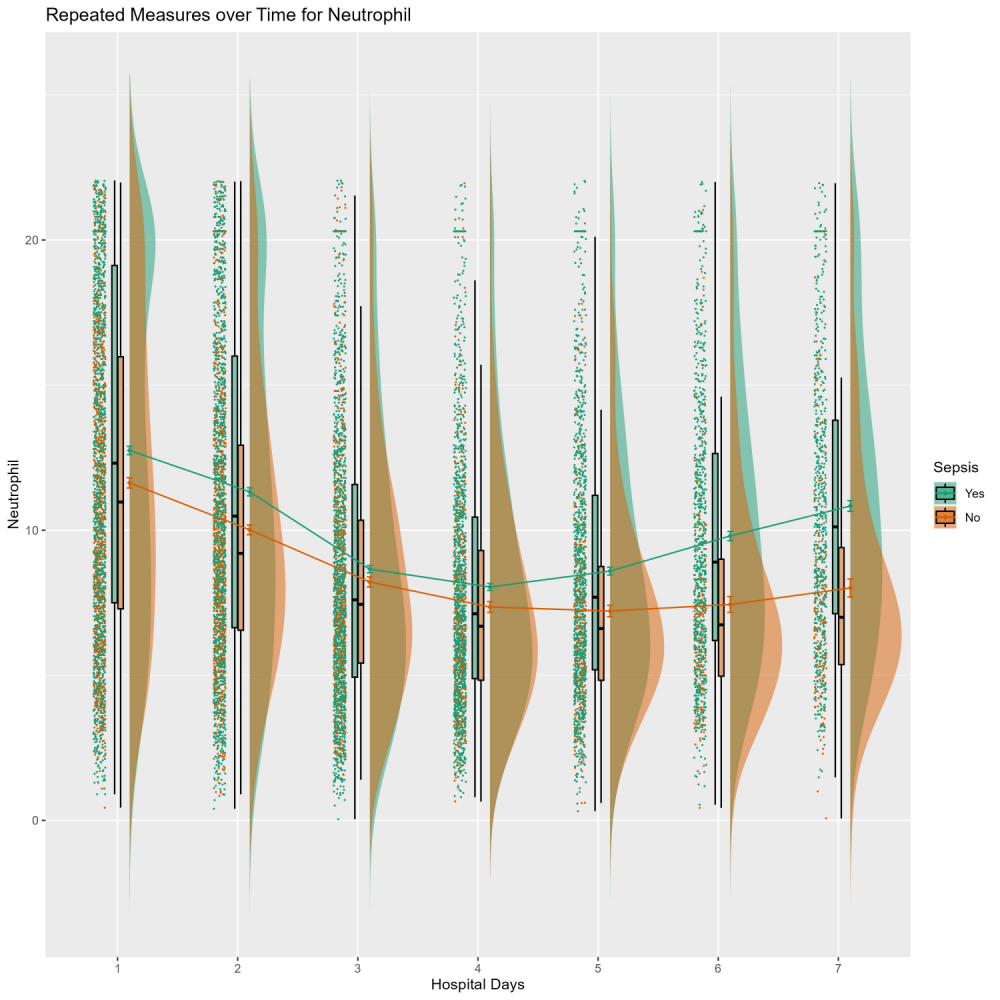


## c) Lymphocyte


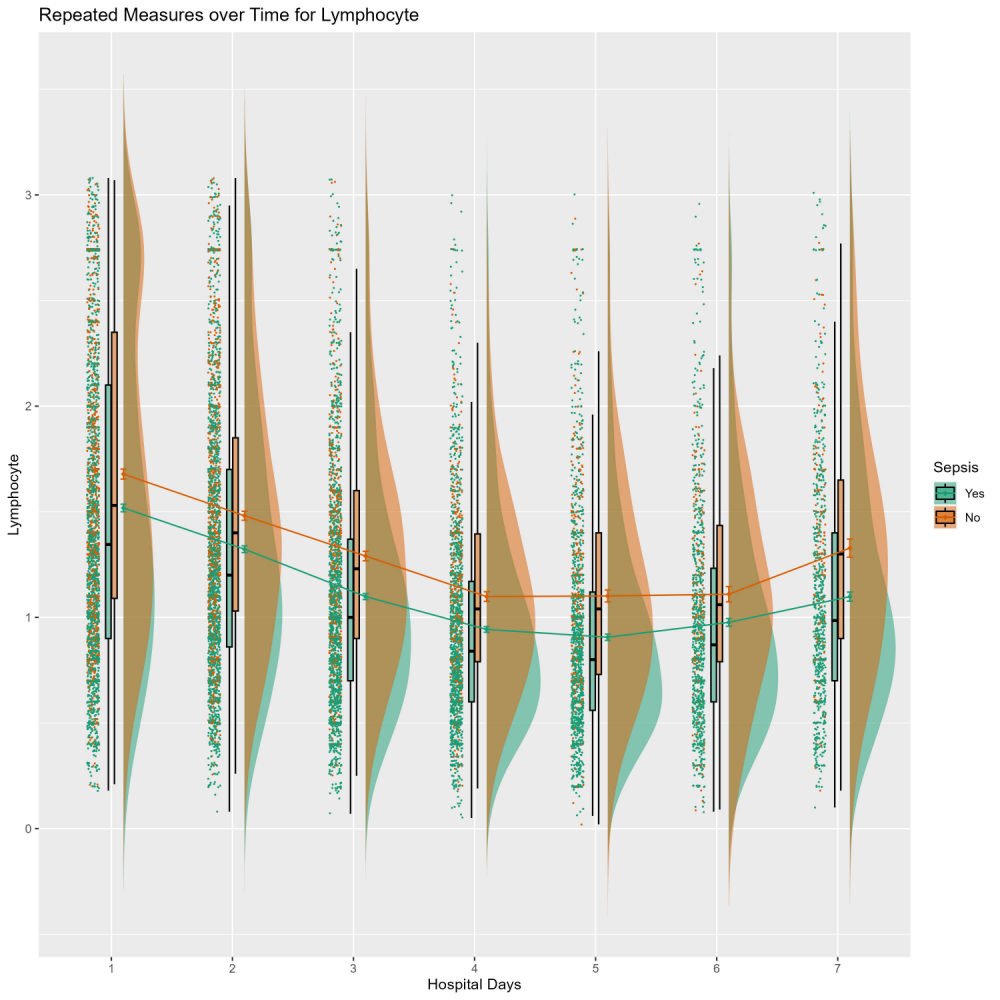


## d) Monocyte


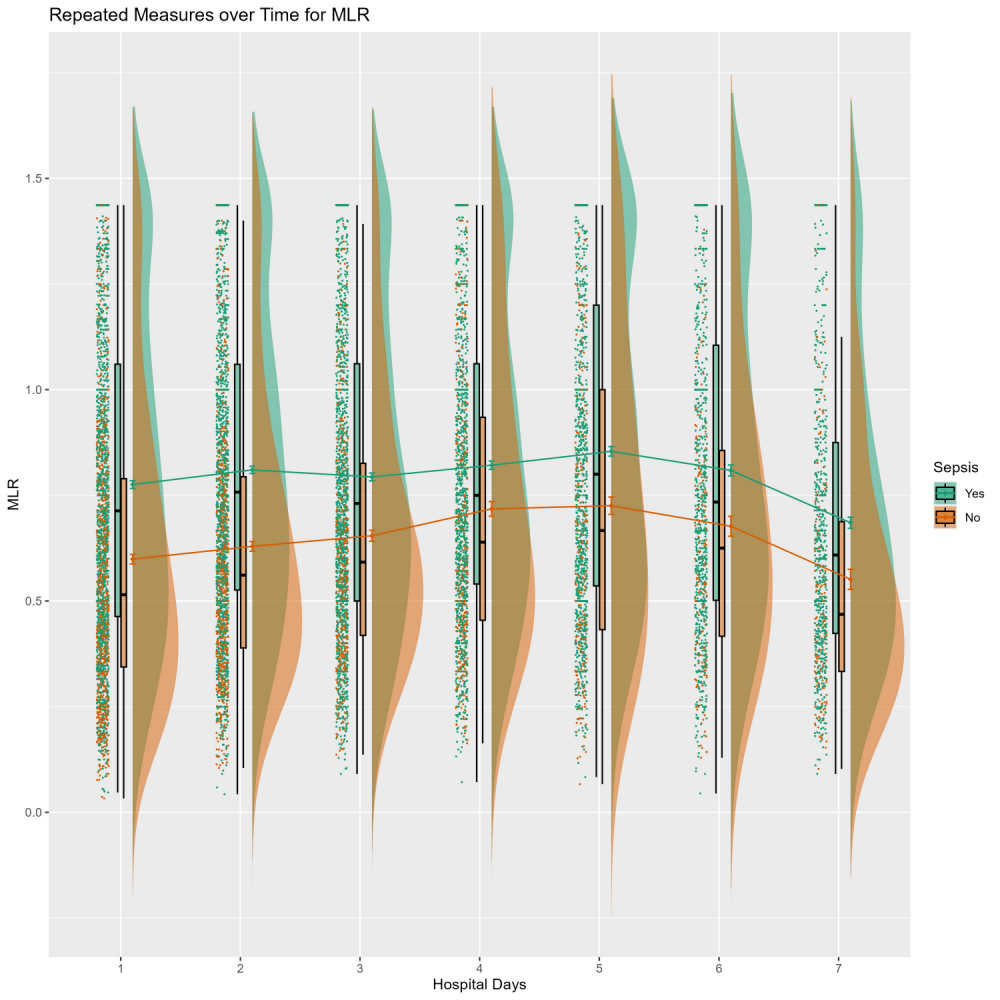


## e) Basophil


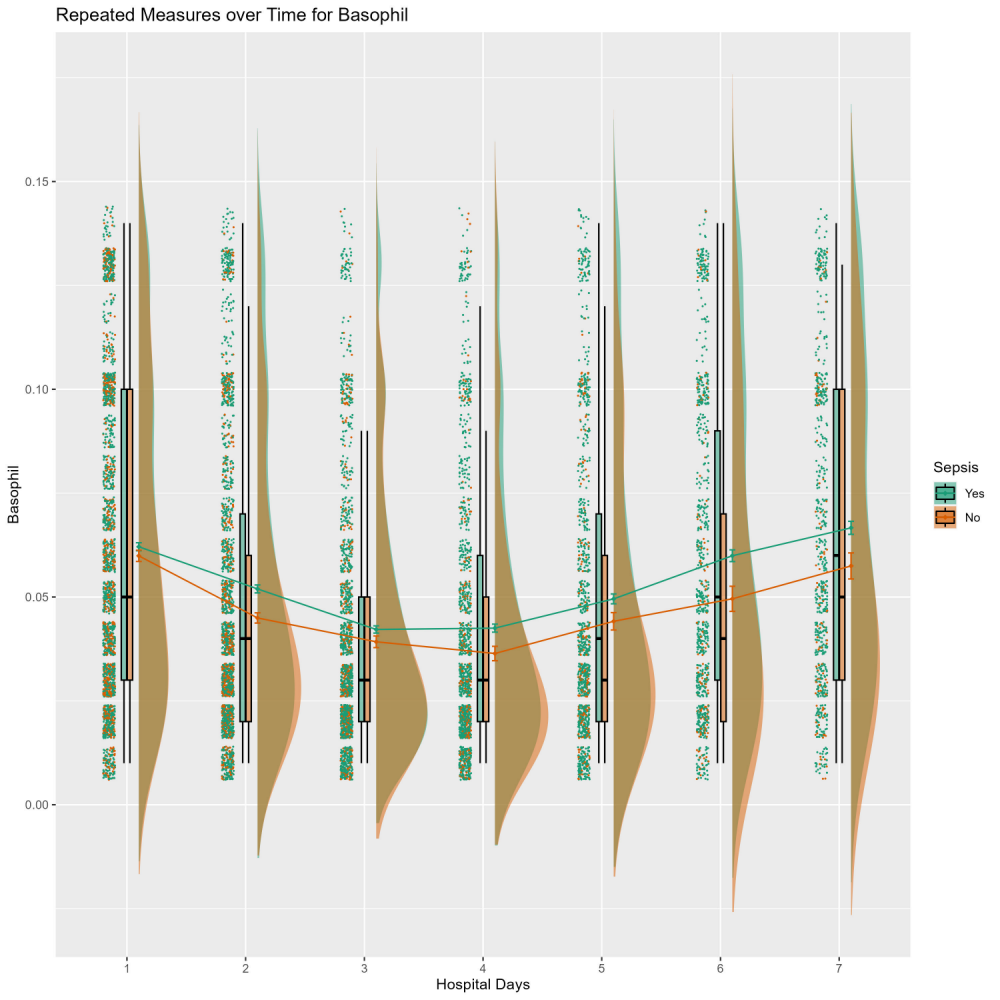


## f) Immature Granulocyte


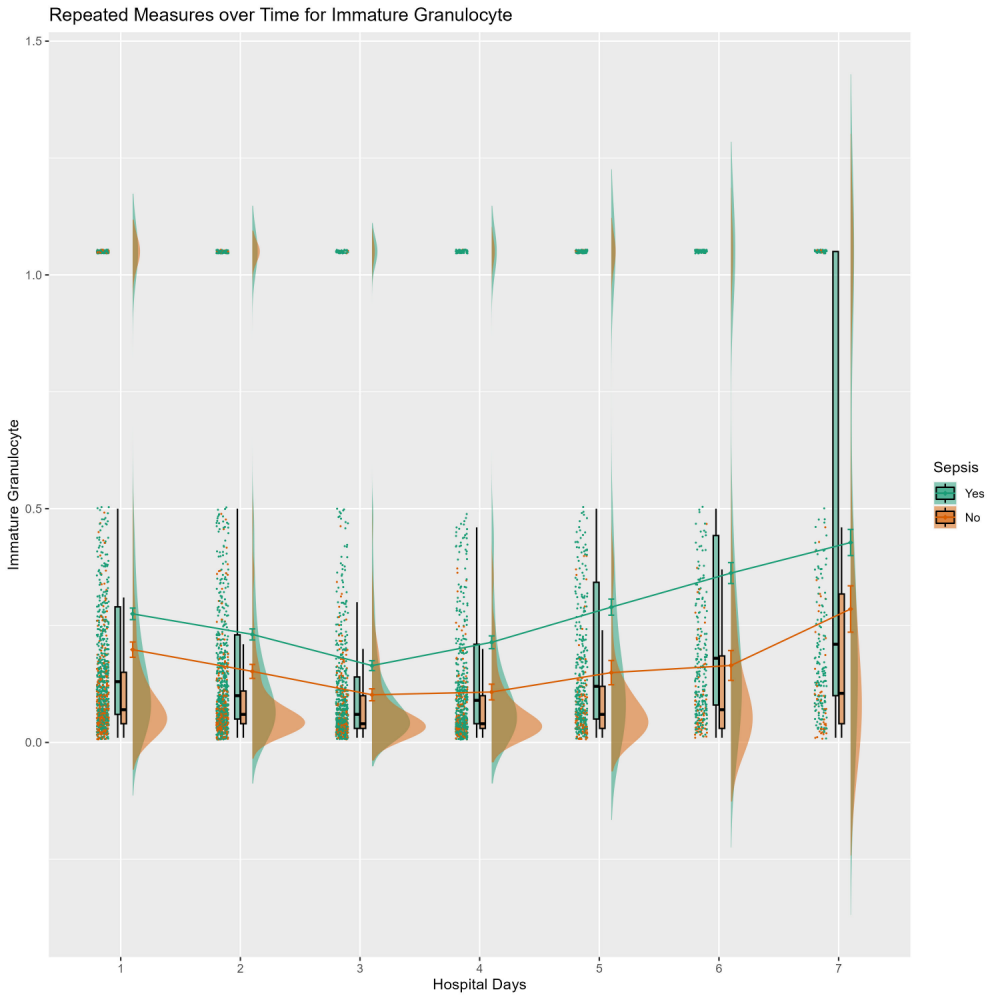


## g) RBC


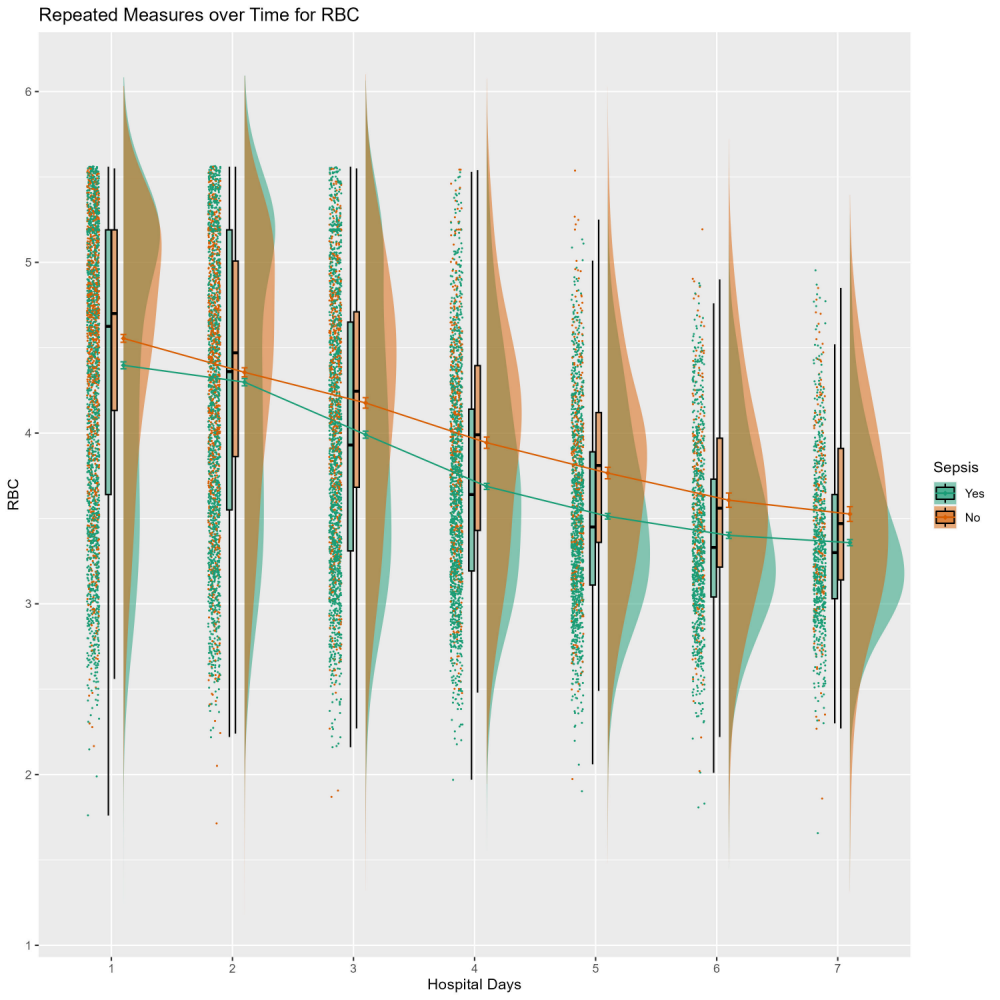


## h) RDW


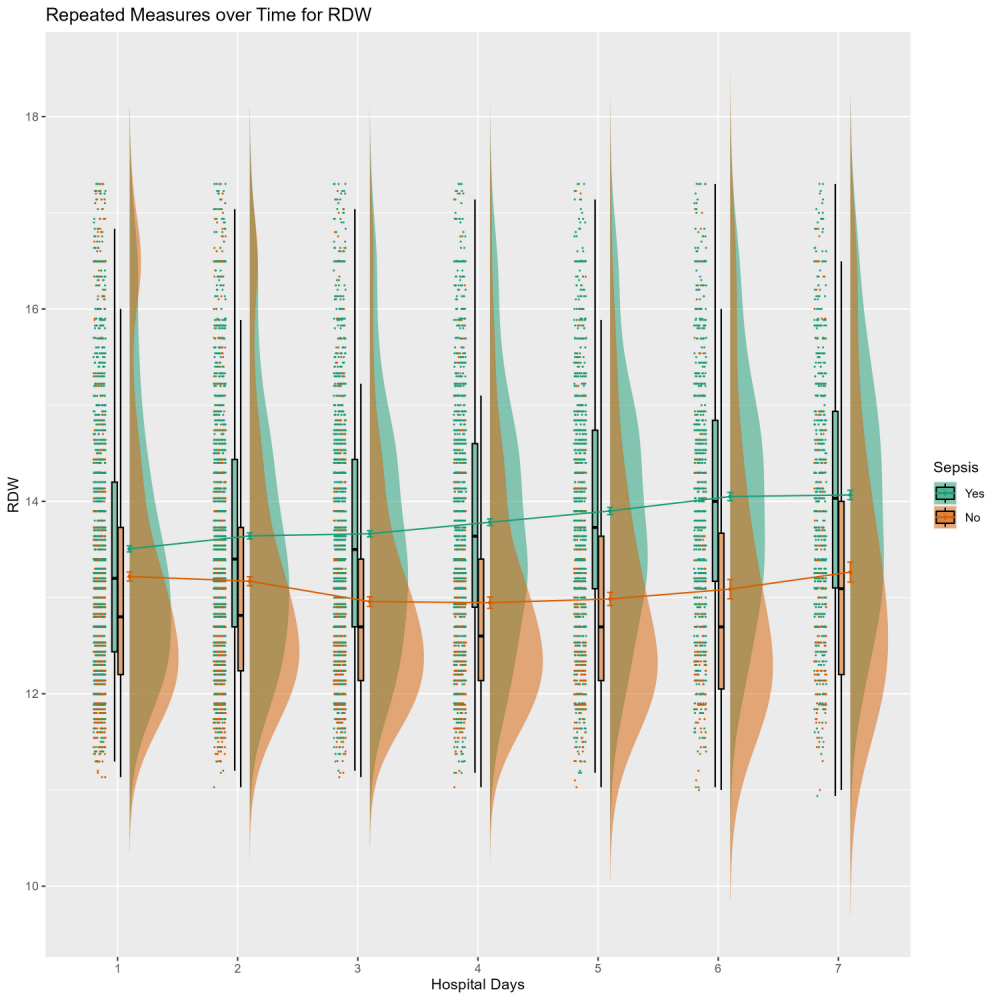


## i) Hct


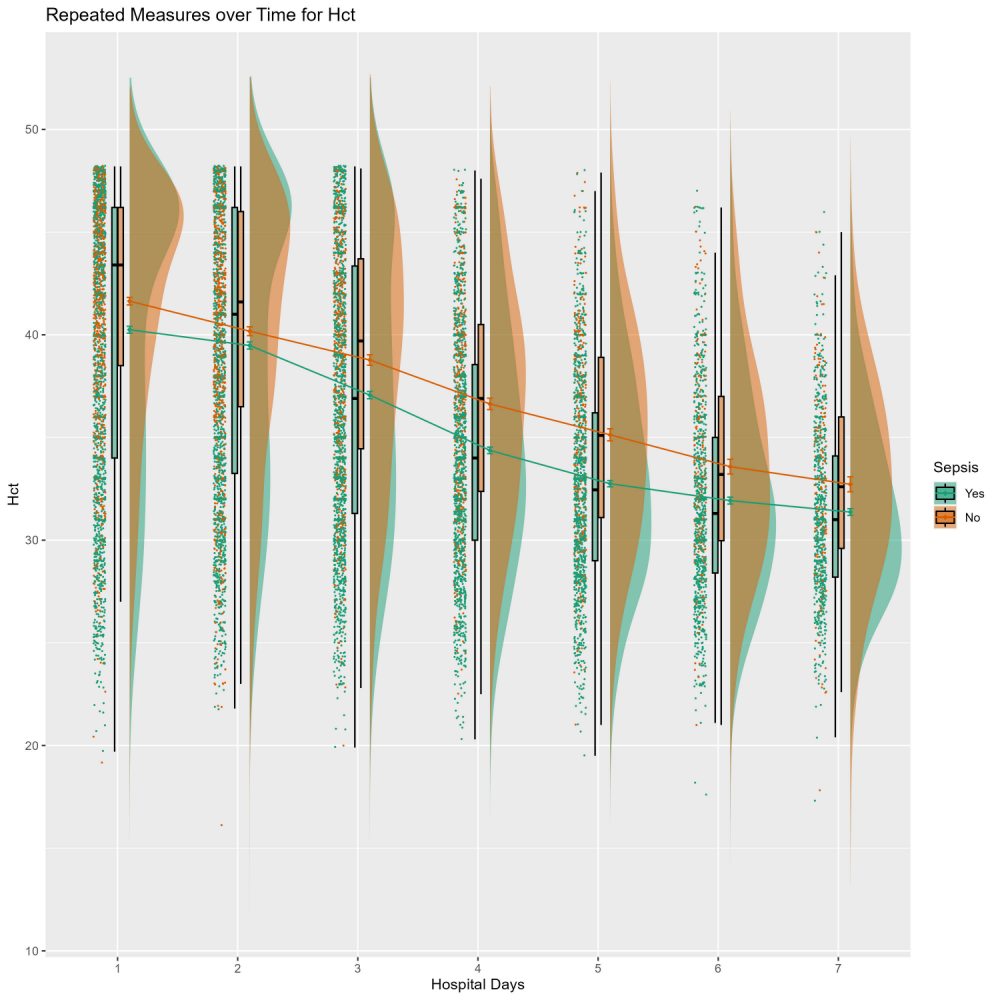


## j) Hb


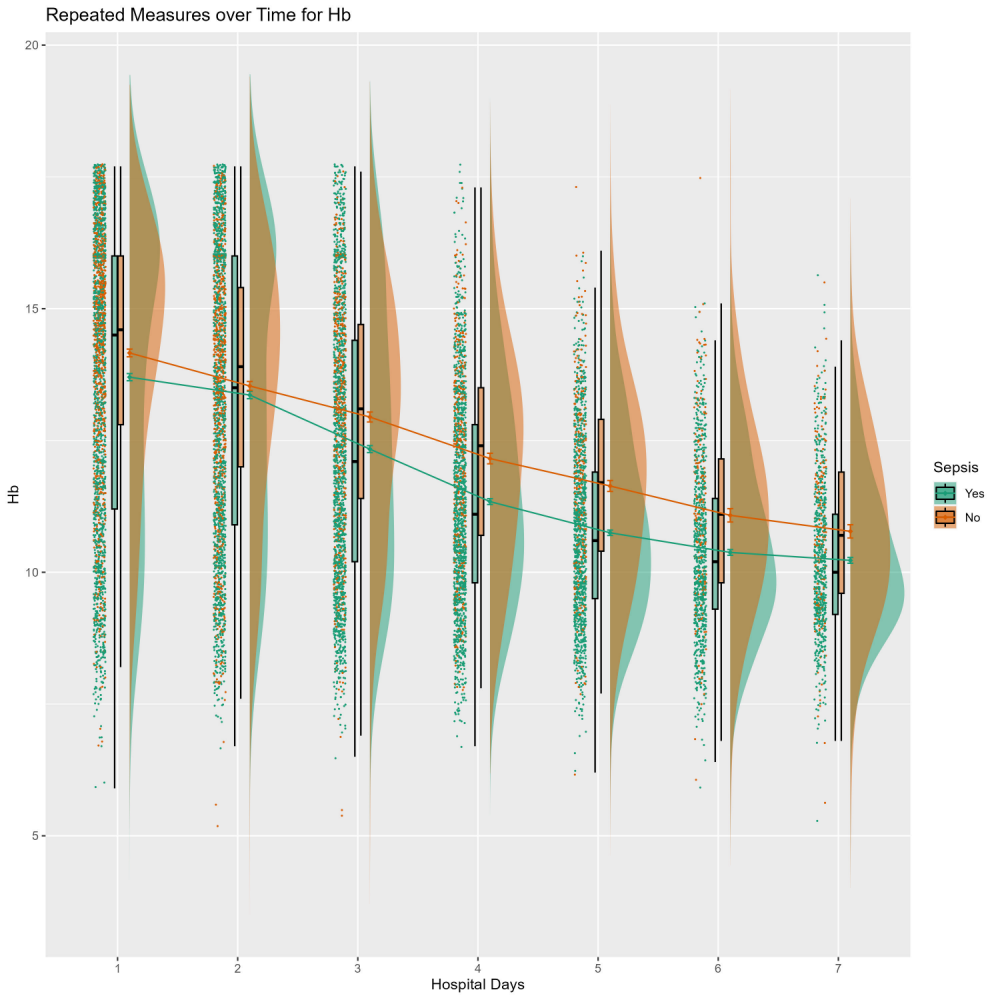


## k) MCV


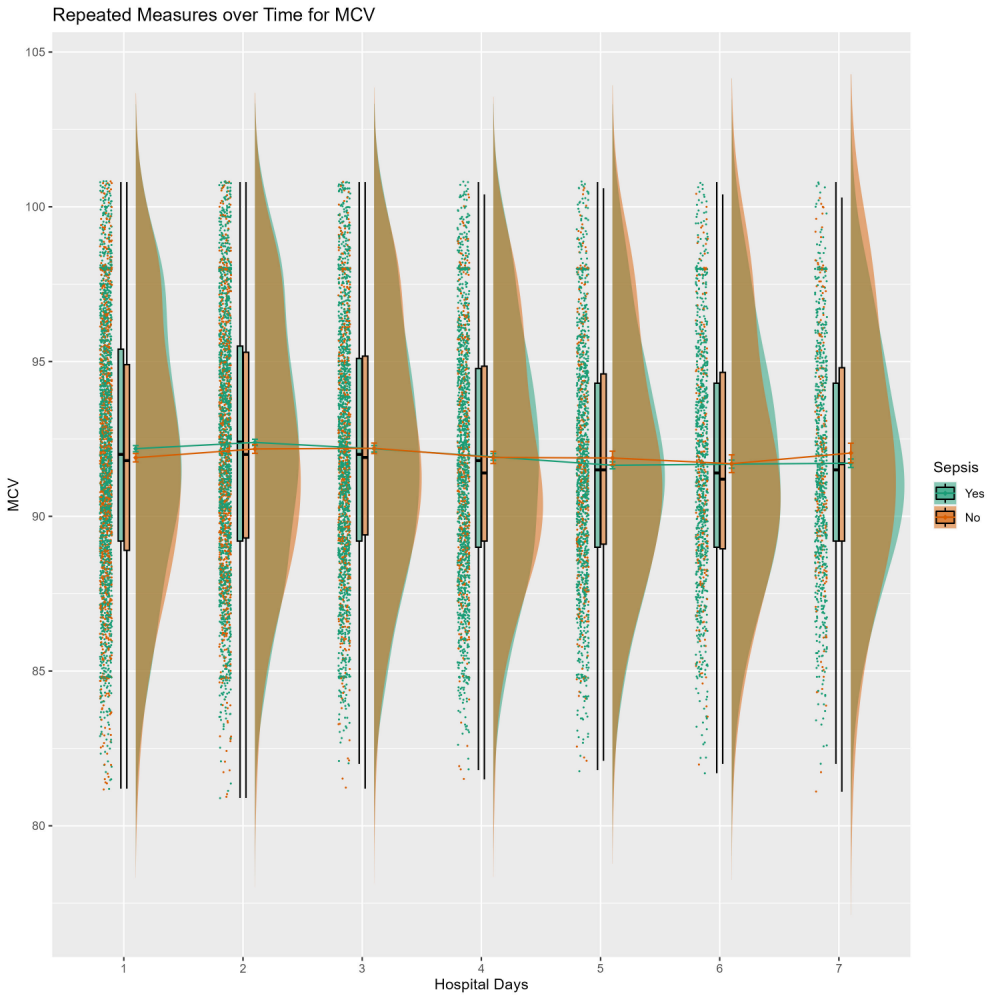


## l) MCH


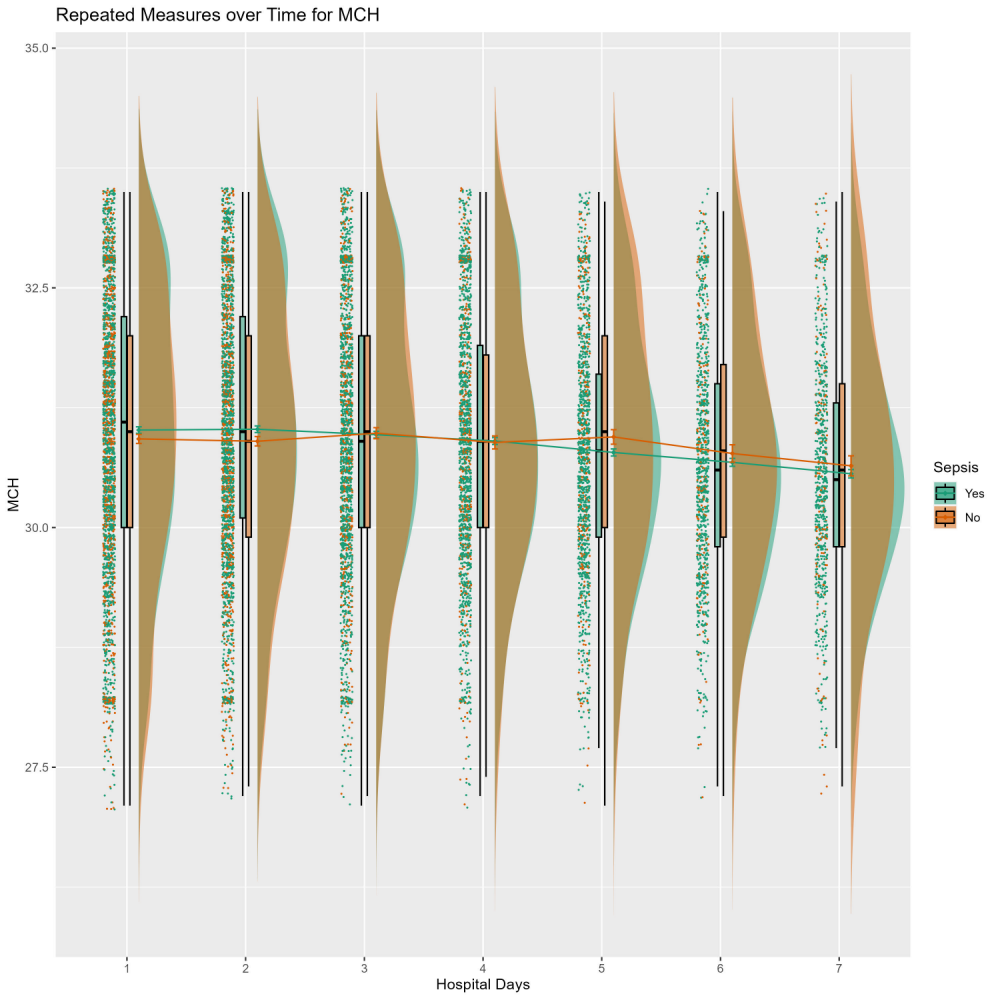


## m) MCHC


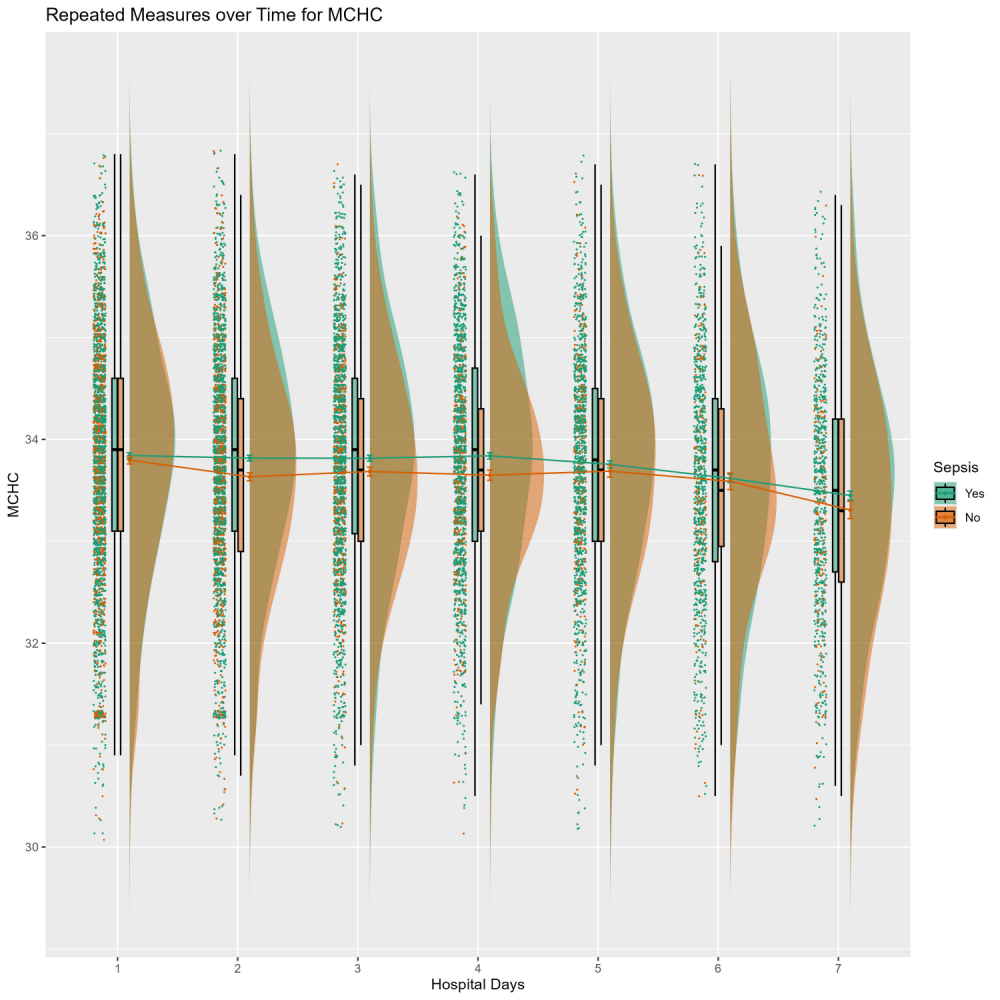


## n) Platelet


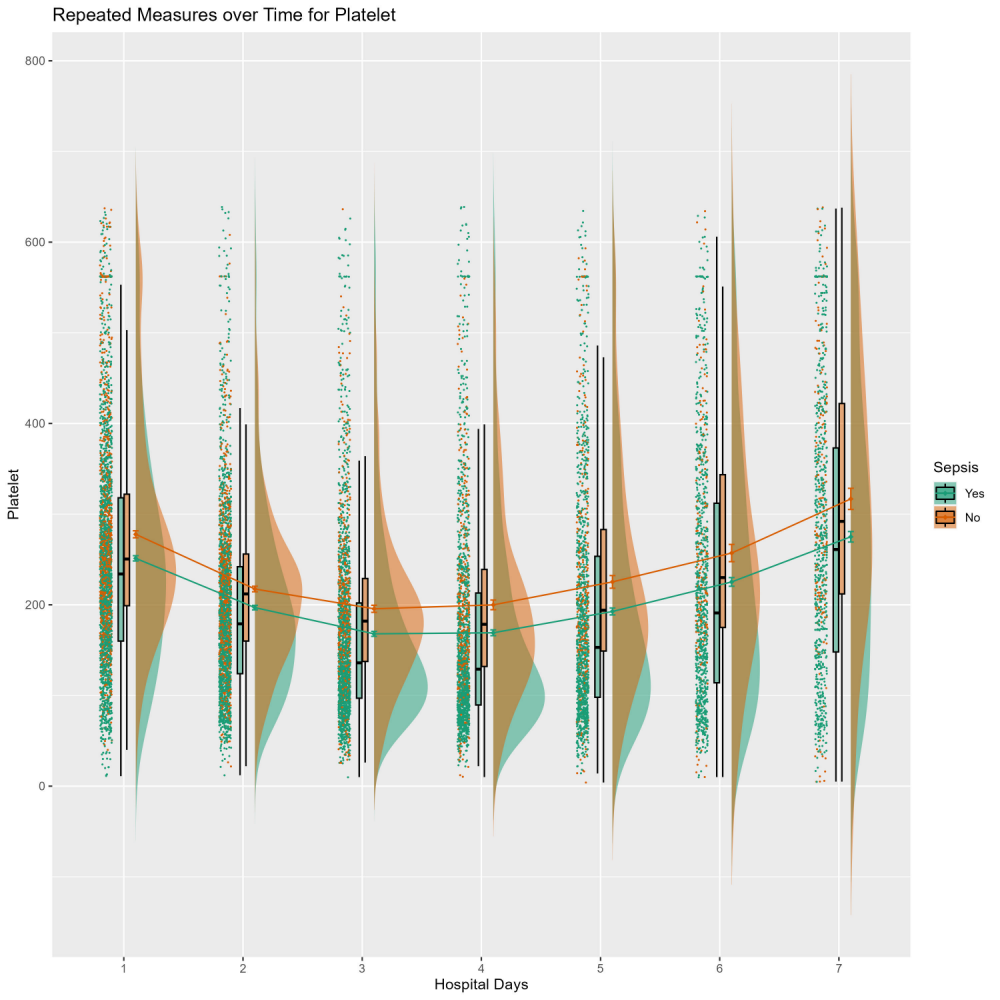


## o) MPV


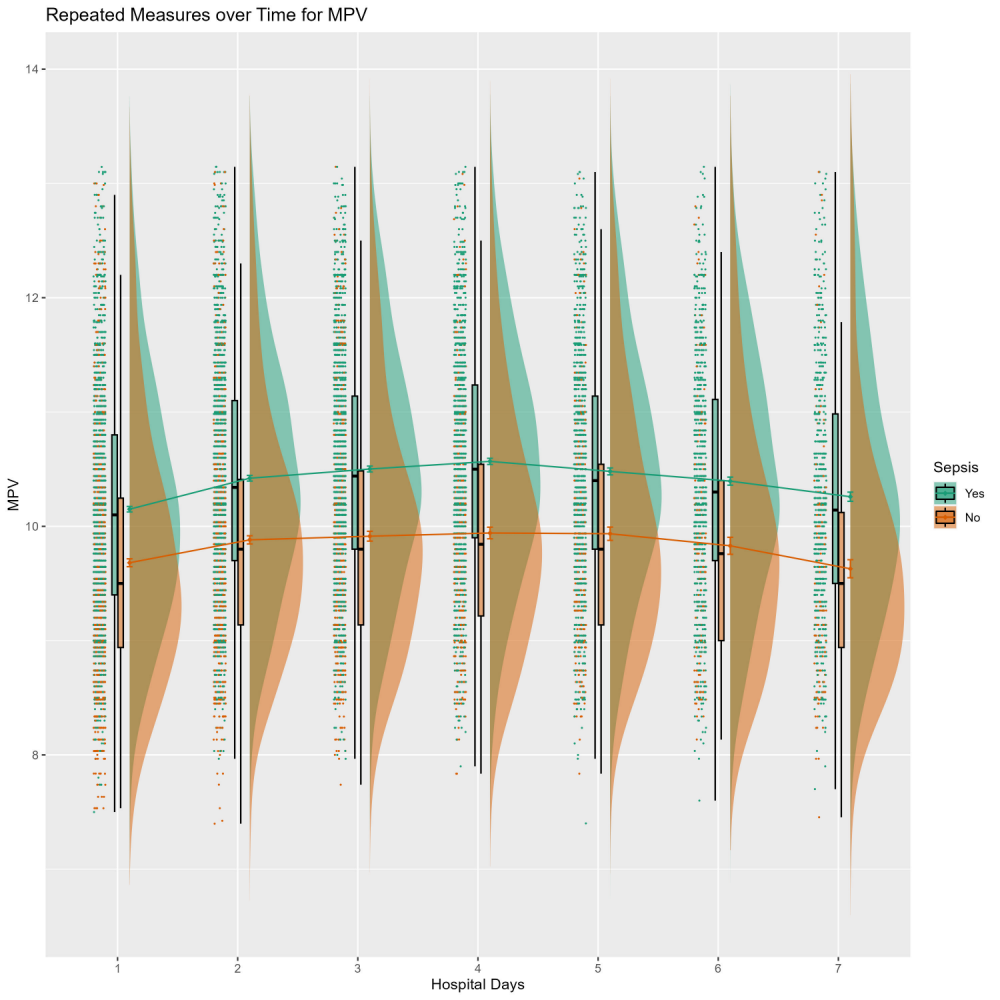


## p) PDW


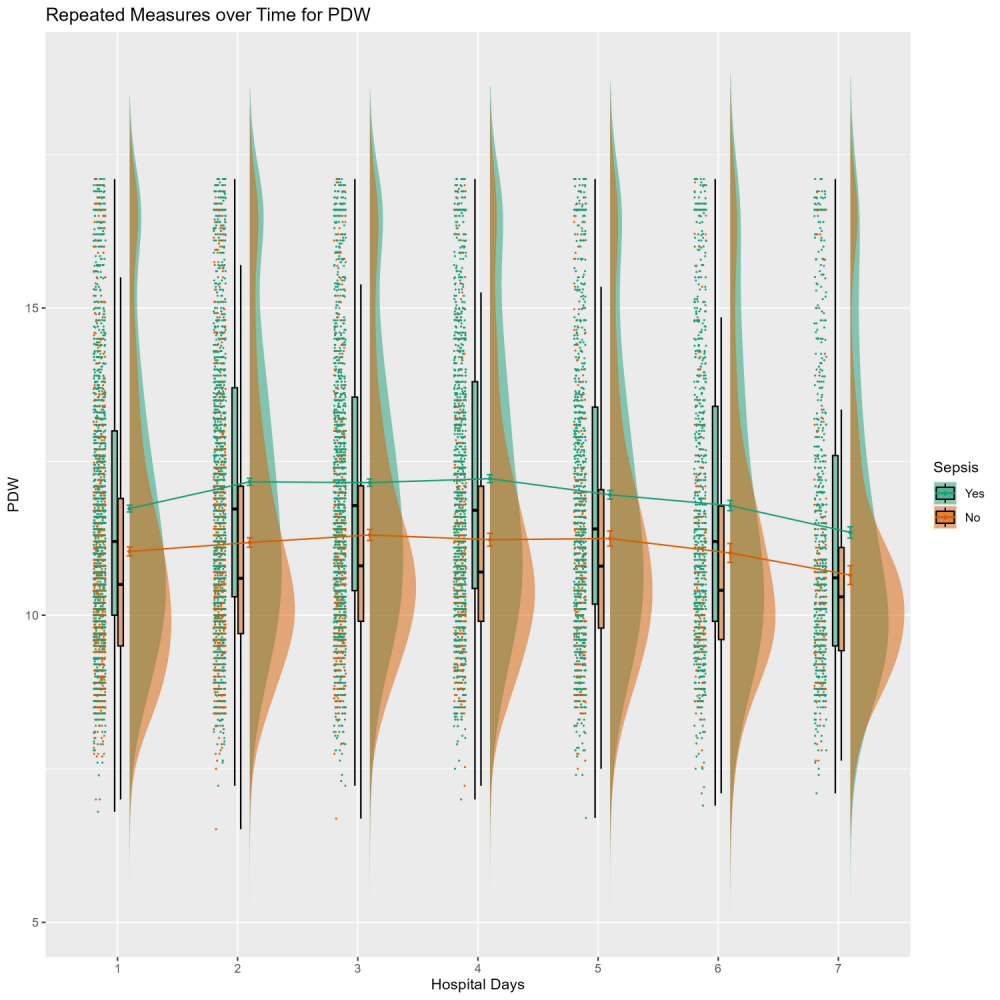


## q) PCT


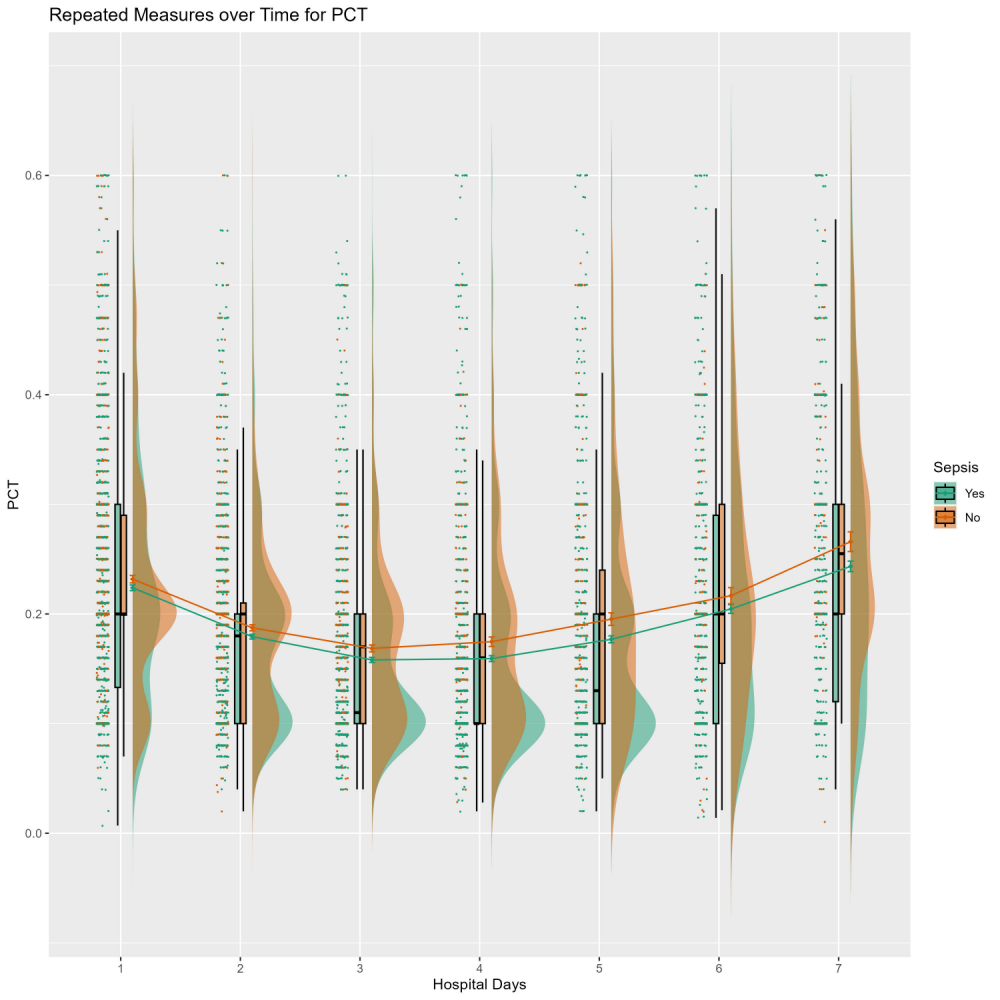


## r) NLR


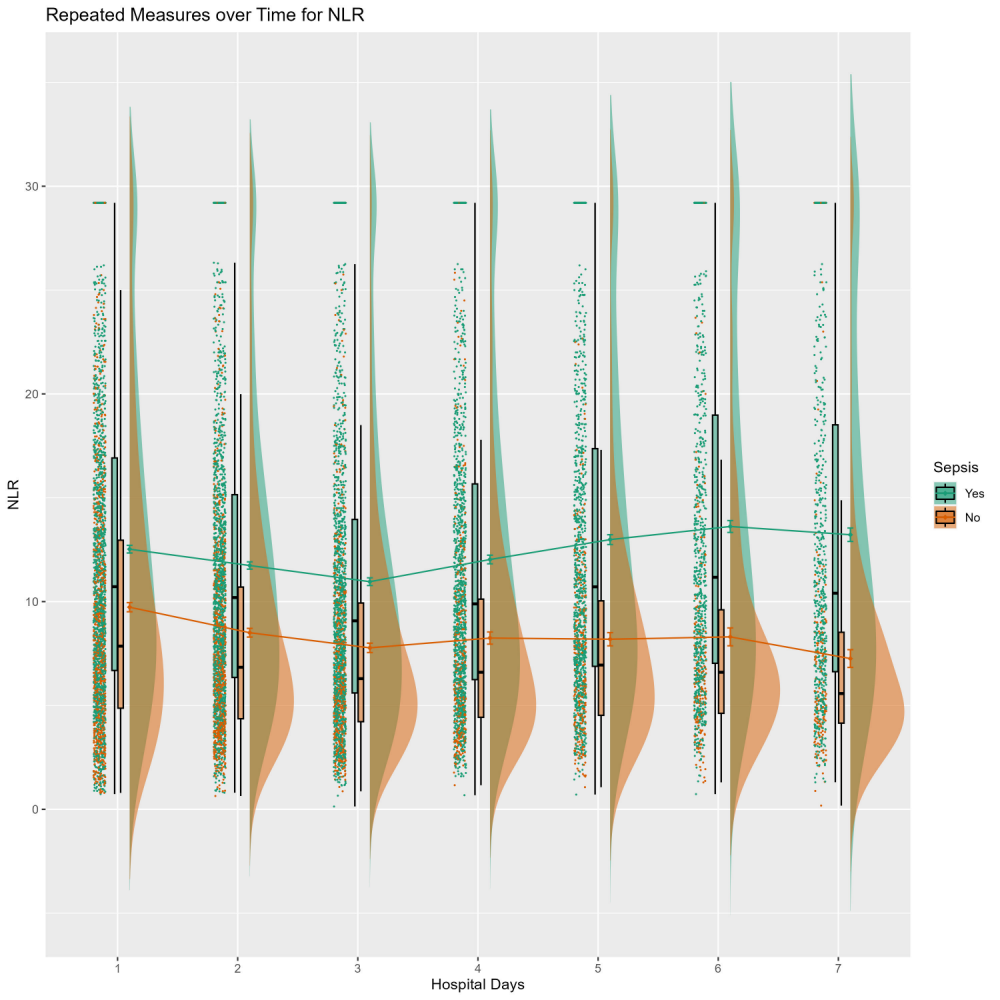


## s) PLR


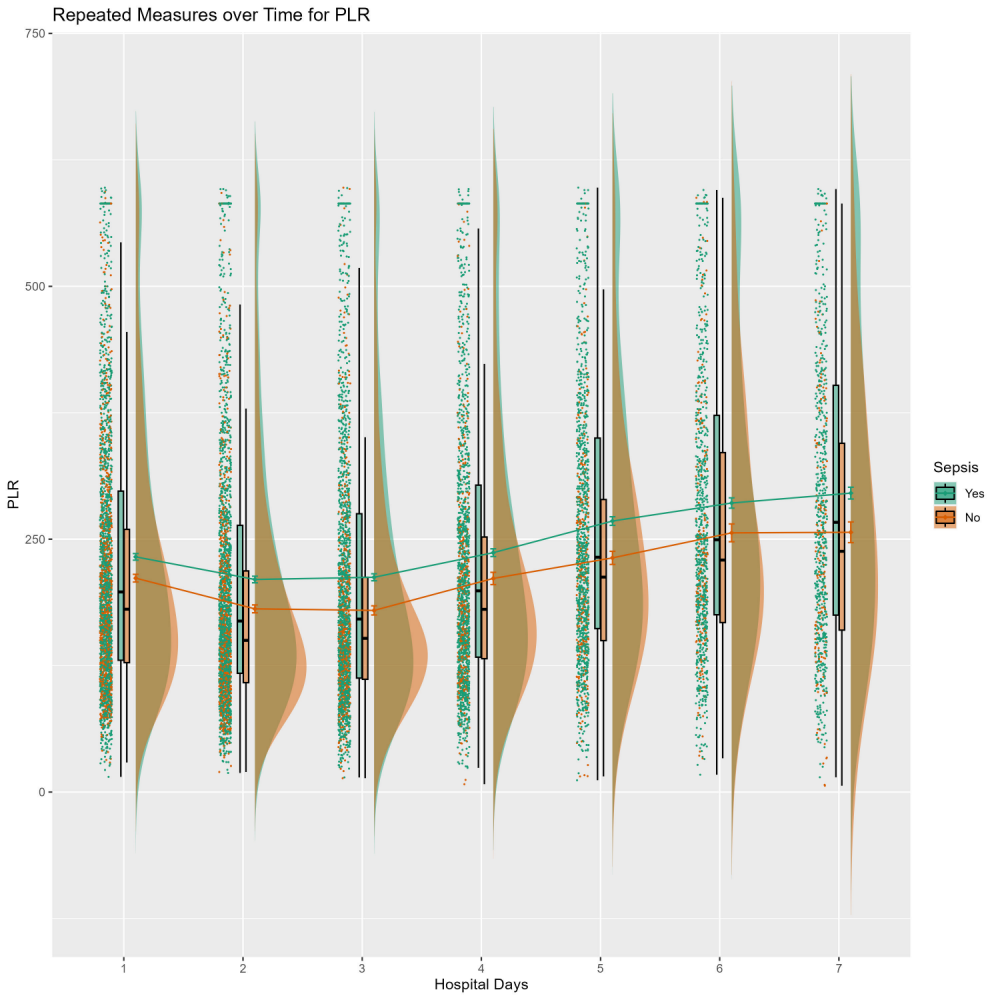


## t) MLR


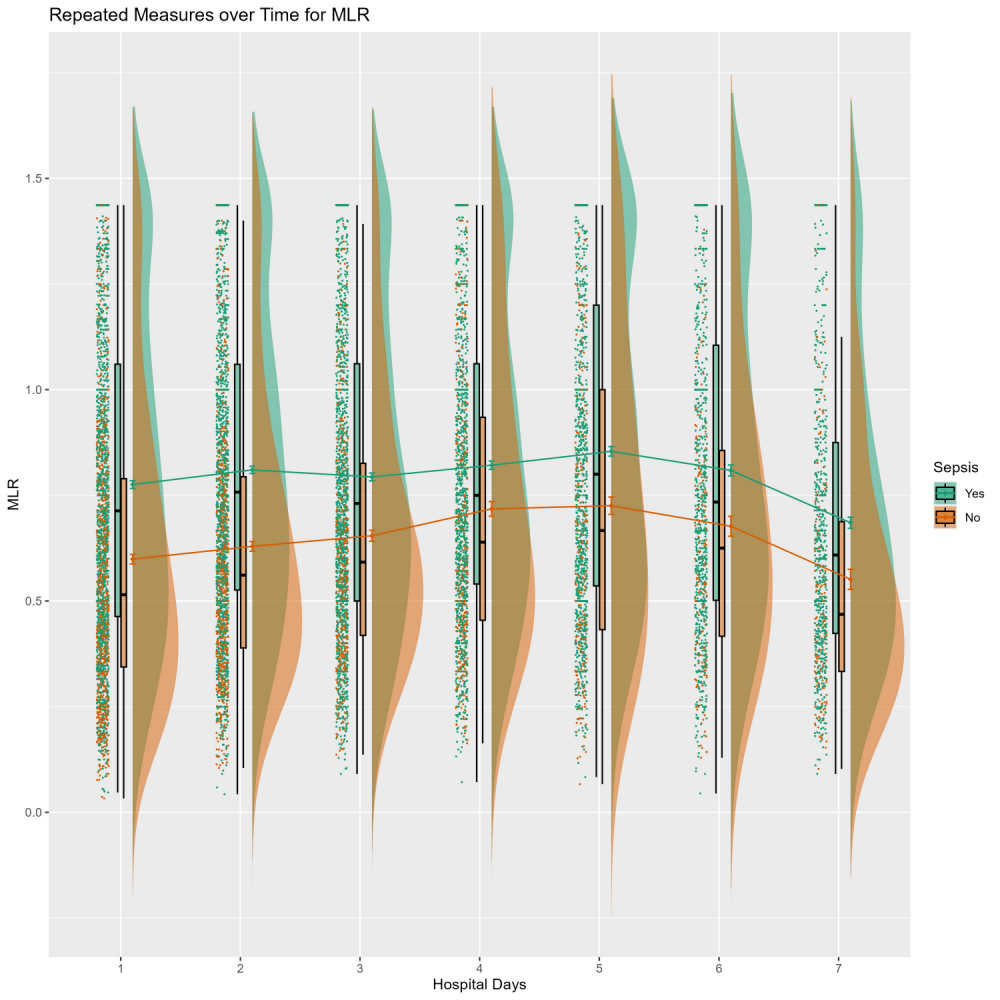


## u) SII


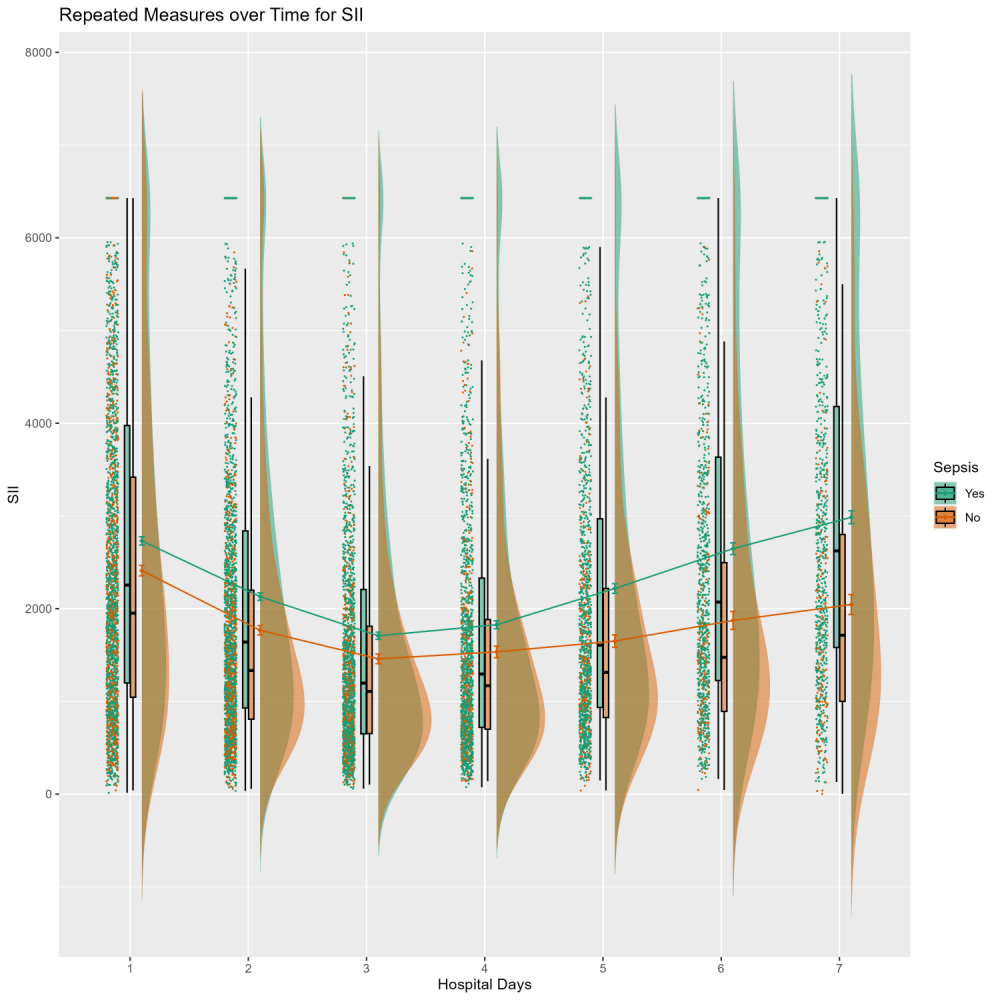


## v) MPVPR


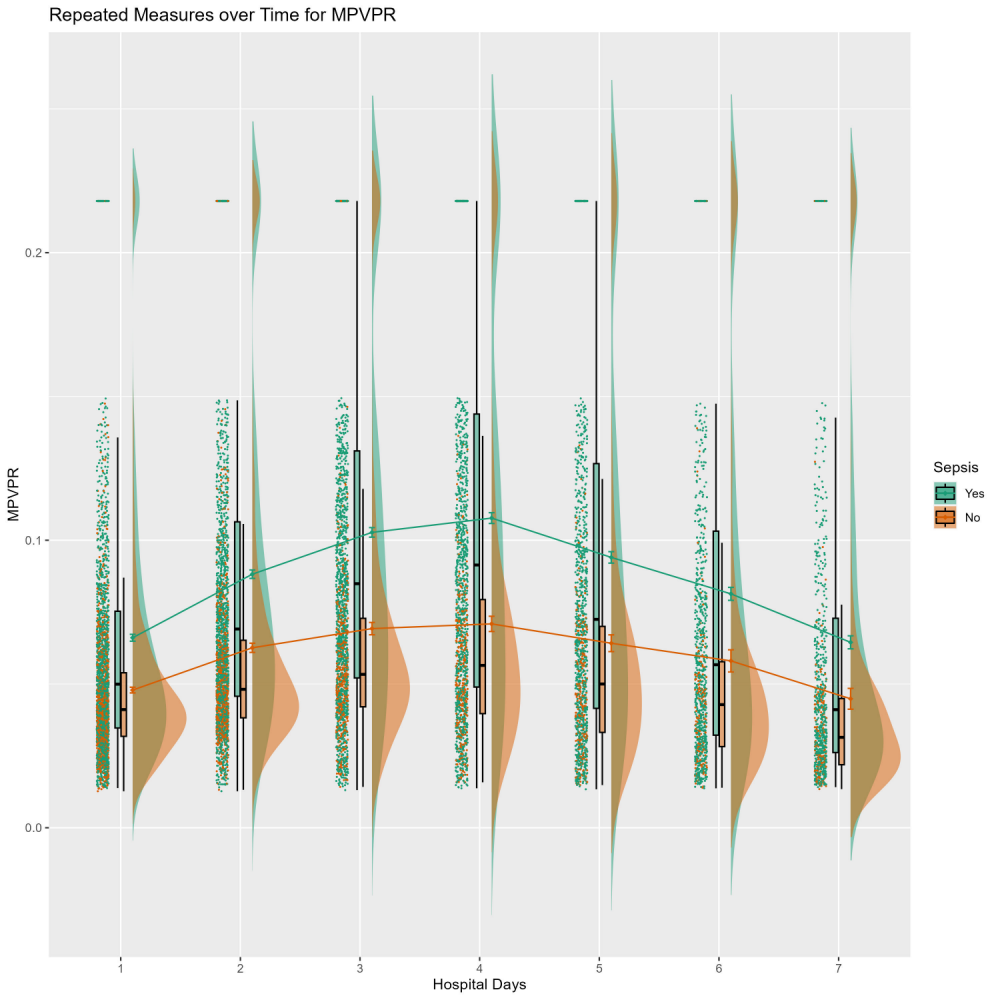


## x) MPVLR


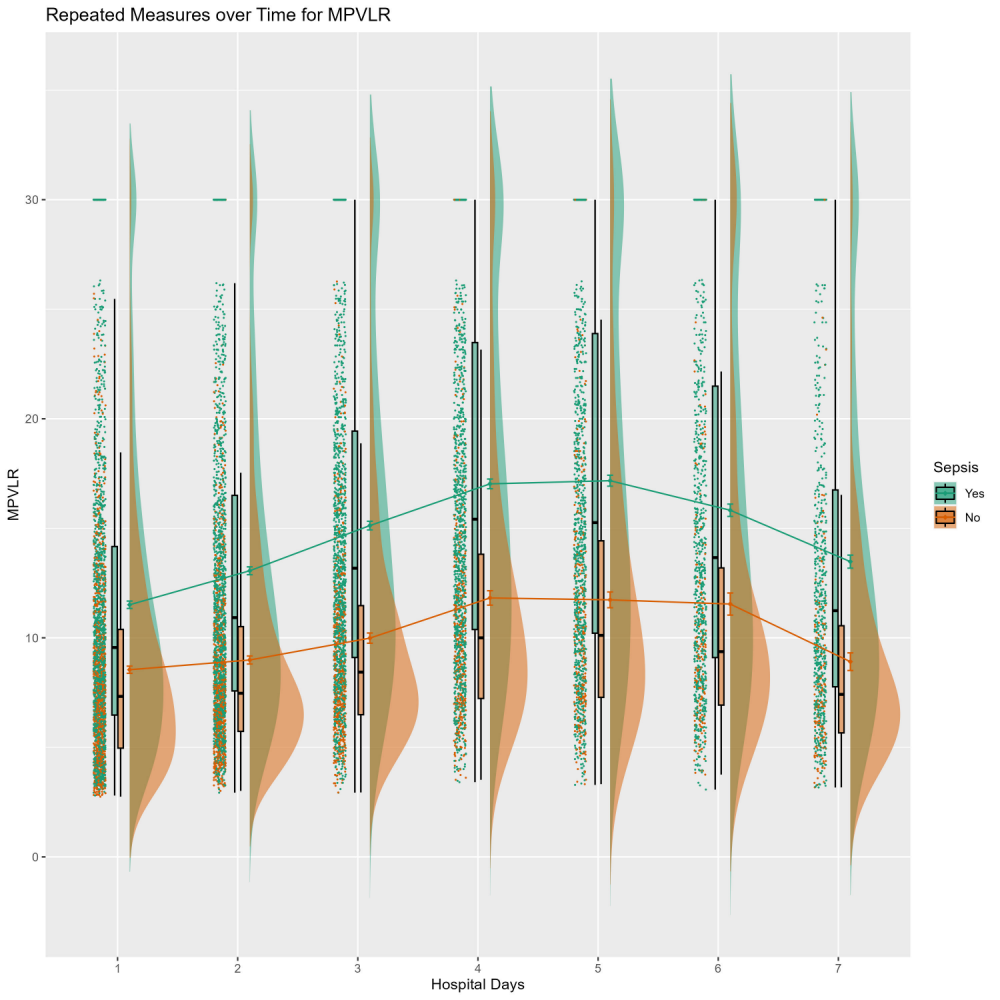


## y) MPVMR


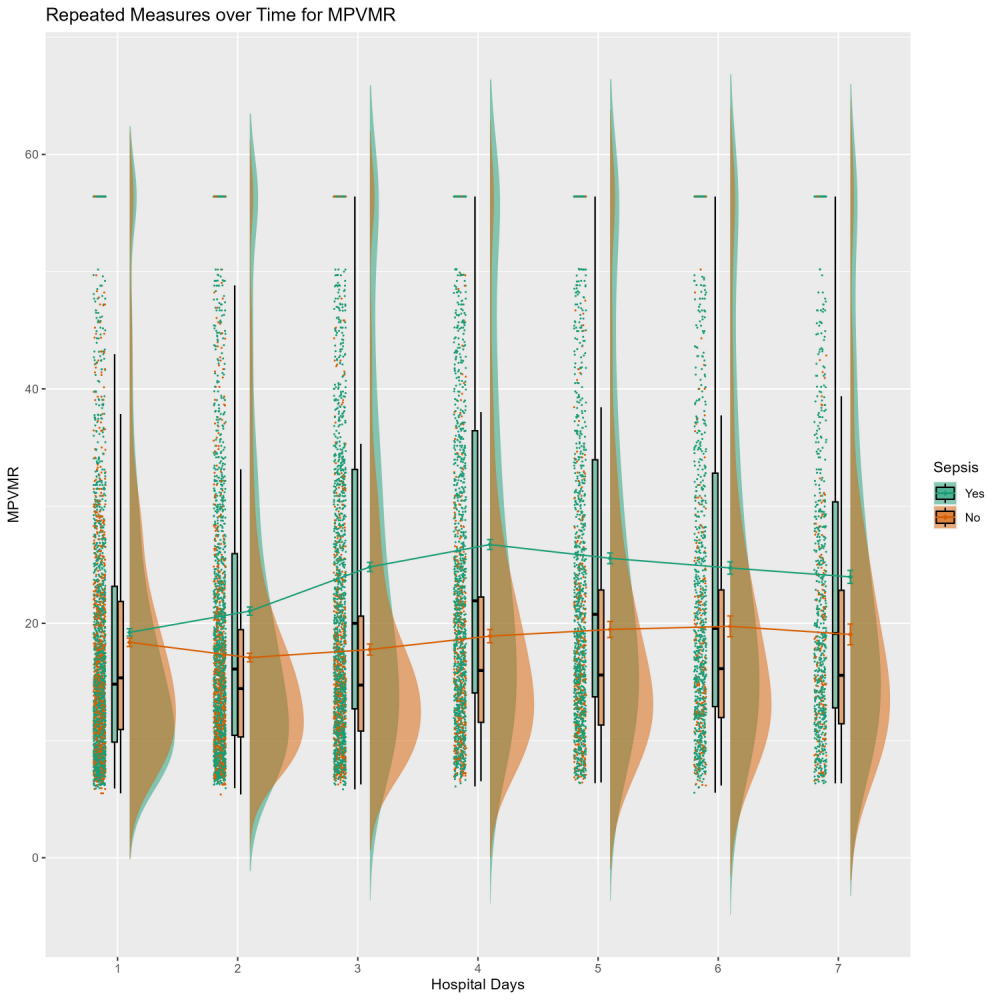


## z) MPVNR


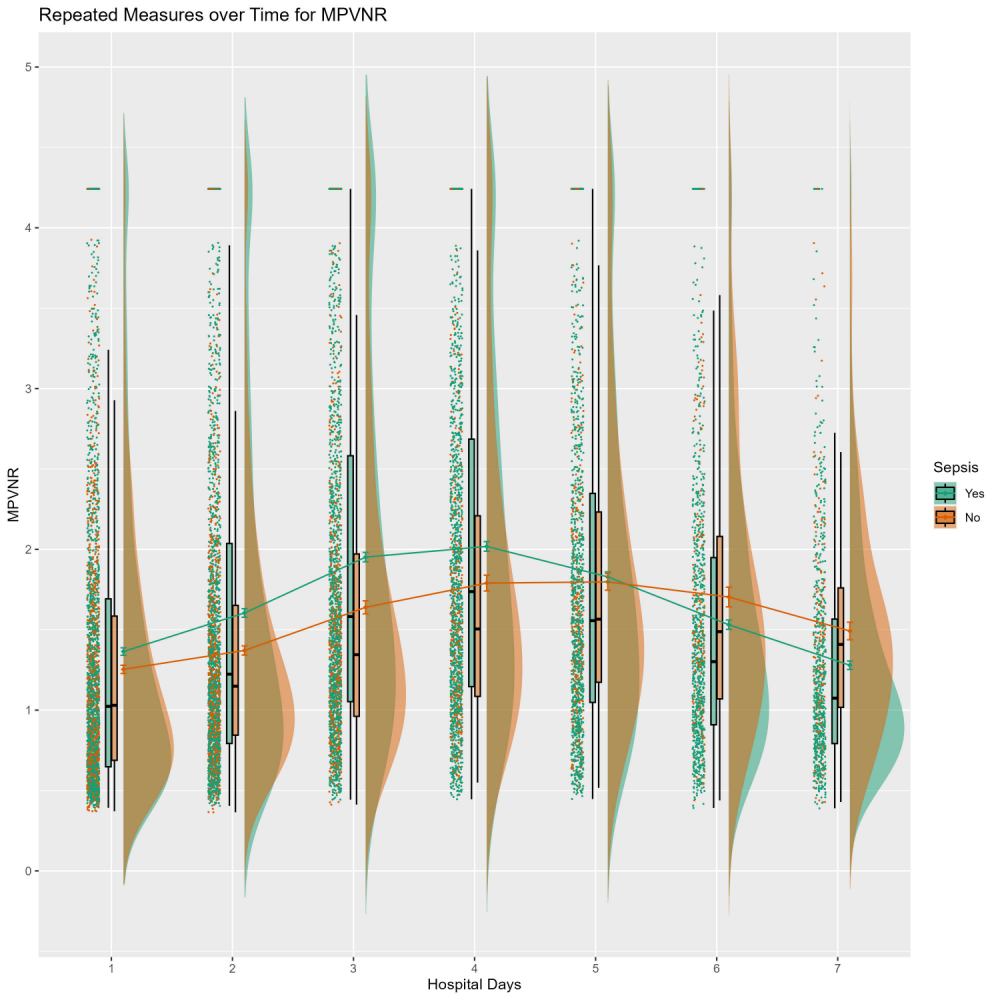


# Table S2. Characteristics of Sepsis Patients by Shock and Mortality

|  | | Shock | | | | Mortality | | |
| --- | --- | --- | --- | --- | --- | --- | --- | --- |
| Group | Variables | Overall, N = 1,806 | Yes, N = 341 (18.9%) | No, N = 1465 (81.1%) | p-value | Yes, N = 484 (26.8%) | **No**, N = 1322 (73.2%) | **p-value** |
| Demographics | Age |  |  |  | 0.034 |  |  | <0.001 |
|  | Median [IQR] | 52 [42, 63] | 54 [42, 66] | 52 [42, 62] |  | 56 [46, 68] | 51 [41, 61] |  |
|  | Sex |  |  |  | 0.826 |  |  | 0.846 |
|  | Male | 1,418 (78.5%) | 266 (78.0%) | 1,152 (78.6%) |  | 382 (78.9%) | 1,036 (78.4%) |  |
|  | Female | 388 (21.5%) | 75 (22.0%) | 313 (21.4%) |  | 102 (21.1%) | 286 (21.6%) |  |
|  | Type |  |  |  | 0.001 |  |  | <0.001 |
|  | FB | 1,340 (74.2%) | 267 (78.3%) | 1,073 (73.2%) |  | 405 (83.7%) | 935 (70.7%) |  |
|  | SB | 173 (9.6%) | 36 (10.6%) | 137 (9.4%) |  | 41 (8.5%) | 132 (10.0%) |  |
|  | EB | 168 (9.3%) | 13 (3.8%) | 155 (10.6%) |  | 13 (2.7%) | 155 (11.7%) |  |
|  | ChB | 26 (1.4%) | 5 (1.5%) | 21 (1.4%) |  | 5 (1.0%) | 21 (1.6%) |  |
|  | CoB | 99 (5.5%) | 20 (5.9%) | 79 (5.4%) |  | 20 (4.1%) | 79 (6.0%) |  |
|  | TBSA |  |  |  | <0.001 |  |  | <0.001 |
|  | Median [IQR] | 33 [20, 51] | 50 [31, 69] | 30 [19, 46] |  | 60 [36, 80] | 28 [17, 41] |  |
|  | Inhalation | 757 (41.9%) | 156 (45.7%) | 601 (41.0%) | 0.114 | 260 (53.7%) | 497 (37.6%) | <0.001 |
|  | LOS |  |  |  | <0.001 |  |  | <0.001 |
|  | Median [IQR] | 21 [10, 35] | 28 [16, 49] | 20 [10, 33] |  | 15 [9, 25] | 23 [11, 40] |  |
| Severity Scores | ABSI |  |  |  | <0.001 |  |  | <0.001 |
|  | Median [IQR] | 8 [7, 10] | 10 [8, 12] | 8 [7, 10] |  | 11 [9, 13] | 8 [6, 9] |  |
|  | rBaux |  |  |  | <0.001 |  |  | <0.001 |
|  | Median [IQR] | 95 [78, 114] | 111 [96, 129] | 91 [76, 109] |  | 121 [105, 140] | 88 [74, 101] |  |
|  | Hangang |  |  |  | <0.001 |  |  | <0.001 |
|  | Median [IQR] | 135 [124, 149] | 148 [138, 159] | 132 [122, 144] |  | 157 [146, 171] | 130 [121, 140] |  |
|  | APACHE_IV |  |  |  | <0.001 |  |  | <0.001 |
|  | Median [IQR] | 44 [29, 63] | 55 [40, 74] | 40 [27, 60] |  | 60 [45, 81] | 37 [25, 55] |  |
|  | SOFA |  |  |  | <0.001 |  |  | <0.001 |
|  | Median [IQR] | 3 [2, 5] | 4 [2, 6] | 3 [2, 5] |  | 5 [3, 7] | 3 [2, 4] |  |
| WBC related | WBC |  |  |  | <0.001 |  |  | <0.001 |
|  | Median [IQR] | 18.3 [12.6, 24.1] | 22.8 [14.8, 24.1] | 17.5 [12.1, 23.8] |  | 24.1 [15.6, 24.1] | 17.0 [12.0, 22.6] |  |
|  | Neutrophil |  |  |  | <0.001 |  |  | <0.001 |
|  | Median [IQR] | 15.2 [9.9, 20.3] | 19.2 [11.5, 20.3] | 14.6 [9.6, 20.1] |  | 19.6 [11.8, 20.3] | 14.1 [9.3, 19.5] |  |
|  | Lymphocyte |  |  |  | 0.001 |  |  | <0.001 |
|  | Median [IQR] | 1.54 [1.02, 2.34] | 1.76 [1.13, 2.74] | 1.50 [1.00, 2.27] |  | 1.81 [1.08, 2.74] | 1.49 [1.00, 2.15] |  |
|  | Monocyte |  |  |  | <0.001 |  |  | <0.001 |
|  | Median [IQR] | 0.94 [0.61, 1.35] | 1.20 [0.71, 1.37] | 0.90 [0.60, 1.28] |  | 1.20 [0.70, 1.37] | 0.88 [0.60, 1.22] |  |
|  | Eosinophil |  |  |  | 0.004 |  |  | 0.545 |
|  | Median [IQR] | 0.10 [0.04, 0.19] | 0.09 [0.03, 0.15] | 0.10 [0.04, 0.20] |  | 0.10 [0.04, 0.20] | 0.10 [0.04, 0.19] |  |
|  | Basophil |  |  |  | 0.007 |  |  | <0.001 |
|  | Median [IQR] | 0.06 [0.03, 0.10] | 0.07 [0.04, 0.10] | 0.06 [0.03, 0.10] |  | 0.08 [0.05, 0.12] | 0.06 [0.03, 0.10] |  |
|  | Immature Granulocyte |  |  |  | <0.001 |  |  | <0.001 |
|  | Median [IQR] | 0.14 [0.07, 0.29] | 0.23 [0.12, 0.64] | 0.12 [0.06, 0.23] |  | 0.25 [0.13, 0.49] | 0.11 [0.06, 0.22] |  |
| RBC related | RBC |  |  |  | 0.027 |  |  | 0.052 |
|  | Median [IQR] | 4.99 [4.23, 5.19] | 5.19 [4.34, 5.19] | 4.95 [4.23, 5.19] |  | 5.19 [4.33, 5.19] | 4.92 [4.23, 5.19] |  |
|  | RDW |  |  |  | <0.001 |  |  | <0.001 |
|  | Median [IQR] | 13.09 [12.40, 14.03] | 13.39 [12.60, 14.53] | 13.00 [12.34, 13.90] |  | 13.70 [12.93, 15.21] | 12.83 [12.30, 13.64] |  |
|  | Hct |  |  |  | 0.030 |  |  | <0.001 |
|  | Median [IQR] | 46.2 [39.7, 46.2] | 46.2 [42.0, 46.2] | 46.0 [39.5, 46.2] |  | 46.2 [41.3, 46.2] | 45.7 [39.5, 46.2] |  |
|  | Hb |  |  |  | 0.075 |  |  | 0.104 |
|  | Median [IQR] | 15.60 [13.30, 16.40] | 16.00 [13.80, 16.50] | 15.40 [13.20, 16.40] |  | 16.00 [13.35, 16.50] | 15.40 [13.20, 16.40] |  |
|  | MCV |  |  |  | 0.393 |  |  | <0.001 |
|  | Median [IQR] | 92.1 [89.1, 95.4] | 92.2 [89.4, 96.1] | 92.1 [89.0, 95.3] |  | 92.7 [89.8, 96.4] | 92.0 [88.9, 95.0] |  |
|  | MCH |  |  |  | 0.711 |  |  | 0.444 |
|  | Median [IQR] | 31.20 [30.10, 32.30] | 31.20 [30.00, 32.23] | 31.20 [30.10, 32.40] |  | 31.10 [30.00, 32.30] | 31.20 [30.10, 32.40] |  |
|  | MCHC |  |  |  | 0.228 |  |  | <0.001 |
|  | Median [IQR] | 34.10 [33.30, 34.80] | 34.00 [33.08, 34.80] | 34.10 [33.30, 34.80] |  | 33.80 [32.90, 34.50] | 34.10 [33.40, 34.80] |  |
| Platelet related | Platelet |  |  |  | 0.362 |  |  | 0.010 |
|  | Median [IQR] | 255 [192, 327] | 262 [191, 341] | 254 [193, 324] |  | 267 [189, 362] | 251 [194, 317] |  |
|  | MPV |  |  |  | <0.001 |  |  | <0.001 |
|  | Median [IQR] | 9.94 [9.34, 10.64] | 10.10 [9.34, 11.00] | 9.94 [9.34, 10.54] |  | 10.25 [9.40, 11.20] | 9.84 [9.30, 10.44] |  |
|  | PDW |  |  |  | <0.001 |  |  | <0.001 |
|  | Median [IQR] | 11.05 [9.91, 12.70] | 11.60 [10.40, 13.41] | 10.90 [9.81, 12.50] |  | 11.89 [10.34, 13.90] | 10.90 [9.80, 12.21] |  |
|  | PCT |  |  |  | 0.221 |  |  | 0.011 |
|  | Median [IQR] | 0.20 [0.20, 0.30] | 0.20 [0.20, 0.30] | 0.20 [0.20, 0.30] |  | 0.20 [0.20, 0.30] | 0.20 [0.19, 0.29] |  |
| Ratios | NLR |  |  |  | 0.014 |  |  | 0.001 |
|  | Median [IQR] | 12 [7, 18] | 13 [8, 20] | 12 [7, 18] |  | 13 [8, 20] | 12 [7, 18] |  |
|  | PLR |  |  |  | 0.018 |  |  | 0.015 |
|  | Median [IQR] | 199 [131, 288] | 182 [119, 272] | 201 [135, 293] |  | 188 [121, 280] | 200 [137, 292] |  |
|  | MLR |  |  |  | 0.012 |  |  | 0.004 |
|  | Median [IQR] | 0.73 [0.48, 1.09] | 0.80 [0.50, 1.25] | 0.71 [0.48, 1.04] |  | 0.80 [0.50, 1.19] | 0.71 [0.47, 1.05] |  |
|  | SII |  |  |  | 0.079 |  |  | 0.083 |
|  | Median [IQR] | 2,736 [1,528, 4,465] | 3,114 [1,569, 4,806] | 2,665 [1,524, 4,374] |  | 2,828 [1,711, 4,744] | 2,698 [1,498, 4,379] |  |
|  | MPVPR |  |  |  | 0.008 |  |  | <0.001 |
|  | Median [IQR] | 0.05 [0.03, 0.06] | 0.05 [0.03, 0.07] | 0.04 [0.03, 0.06] |  | 0.05 [0.04, 0.08] | 0.04 [0.03, 0.06] |  |
|  | MPVLR |  |  |  | 0.386 |  |  | 0.657 |
|  | Median [IQR] | 9 [6, 13] | 9 [5, 13] | 9 [6, 13] |  | 9 [5, 14] | 9 [6, 13] |  |
|  | MPVMR |  |  |  | <0.001 |  |  | 0.134 |
|  | Median [IQR] | 13 [9, 20] | 12 [8, 19] | 14 [10, 21] |  | 13 [8, 21] | 13 [9, 20] |  |
|  | MPVNR |  |  |  | <0.001 |  |  | 0.003 |
|  | Median [IQR] | 0.83 [0.57, 1.37] | 0.73 [0.53, 1.19] | 0.85 [0.58, 1.40] |  | 0.74 [0.54, 1.27] | 0.86 [0.59, 1.38] |  |

# Table S3. Odds Ratios for Septic Shock Diagnosis Using Generalized Estimating Equations Model

| CBC parameters | Variables | Odd Ratio (95%CI) | p-value | Odd ratio over time (95%CI) | p-value | adjusted Odd Ratio (95%CI) | p-value | adjusted Odd ratio over time (95%CI) | p-value |
| --- | --- | --- | --- | --- | --- | --- | --- | --- | --- |
| WBC-realated | WBC | 1.003 (1.002 - 1.005) | <0.001 ** | 1.000 (0.999 - 1.000) | 0.250 | 1.003 (1.000 - 1.006) | 0.040 * | 1.000 (0.999 - 1.001) | 0.754 |
|  | Neutrophil | 1.003 (1.001 - 1.004) | <0.001 ** | 1.000 (1.000 - 1.000) | 0.955 | 1.003 (1.000 - 1.006) | 0.046 * | 1.000 (0.999 - 1.001) | 0.496 |
|  | Lymphocyte | 1.002 (1.001 - 1.003) | 0.006 * | 0.999 (0.999 - 1.000) | <0.001 ** | 1.001 (0.998 - 1.004) | 0.529 | 1.000 (0.999 - 1.000) | 0.191 |
|  | Monocyte | 1.003 (1.002 - 1.005) | <0.001 ** | 0.999 (0.999 - 1.000) | <0.001 ** | 1.003 (1.000 - 1.006) | 0.083 | 1.000 (0.999 - 1.001) | 0.676 |
|  | Eosinophil | 0.992 (0.988 - 0.996) | <0.001 ** | 1.000 (0.999 - 1.002) | 0.350 | 0.985 (0.978 - 0.992) | <0.001 ** | 1.002 (1.000 - 1.004) | 0.052 |
|  | Basophil | 1.002 (0.999 - 1.005) | 0.107 | 1.000 (0.999 - 1.001) | 0.716 | 0.998 (0.993 - 1.004) | 0.525 | 1.001 (1.000 - 1.002) | 0.200 |
|  | Immature Granulocyte | 1.008 (1.004 - 1.012) | <0.001 ** | 0.999 (0.998 - 1.000) | 0.068 | 1.008 (1.002 - 1.014) | 0.008 * | 0.999 (0.998 - 1.000) | 0.128 |
| RBC-realated | RBC | 1.003 (1.000 - 1.005) | 0.052 | 0.999 (0.999 - 1.000) | 0.016 * | 1.000 (0.995 - 1.004) | 0.828 | 1.000 (0.999 - 1.001) | 0.916 |
|  | RDW | 1.011 (1.006 - 1.016) | <0.001 ** | 1.001 (1.001 - 1.002) | <0.001 ** | 1.012 (1.004 - 1.021) | 0.005 * | 1.001 (1.000 - 1.002) | 0.041 * |
|  | Hct | 1.003 (1.000 - 1.005) | 0.044 * | 1.000 (0.999 - 1.000) | 0.151 | 1.000 (0.996 - 1.004) | 0.992 | 1.000 (1.000 - 1.001) | 0.385 |
|  | Hb | 1.002 (1.000 - 1.005) | 0.063 | 0.999 (0.999 - 1.000) | 0.013 * | 1.000 (0.995 - 1.004) | 0.875 | 1.000 (0.999 - 1.001) | 0.938 |
|  | MCV | 1.001 (0.996 - 1.005) | 0.763 | 1.001 (1.000 - 1.001) | 0.004 * | 1.000 (0.992 - 1.007) | 0.941 | 1.001 (1.000 - 1.002) | 0.060 |
|  | MCH | 0.999 (0.995 - 1.002) | 0.449 | 1.000 (1.000 - 1.001) | 0.140 | 0.998 (0.991 - 1.005) | 0.639 | 1.001 (1.000 - 1.002) | 0.144 |
|  | MCHC | 0.999 (0.997 - 1.002) | 0.580 | 1.000 (0.999 - 1.000) | 0.325 | 0.999 (0.995 - 1.003) | 0.663 | 1.000 (0.999 - 1.001) | 0.967 |
| Platelet-realated | Platelet | 1.000 (0.997 - 1.003) | 0.968 | 0.997 (0.996 - 0.997) | <0.001 ** | 1.000 (0.996 - 1.004) | 0.995 | 0.995 (0.994 - 0.996) | <0.001 ** |
|  | MPV | 1.005 (1.001 - 1.008) | 0.008 * | 1.001 (1.000 - 1.002) | 0.002 * | 1.006 (0.999 - 1.012) | 0.079 | 1.001 (1.000 - 1.002) | 0.037 * |
|  | PDW | 1.001 (0.999 - 1.003) | 0.291 | 1.001 (1.000 - 1.001) | <0.001 ** | 1.000 (0.996 - 1.003) | 0.870 | 1.001 (1.001 - 1.002) | 0.001 * |
|  | PCT | 1.002 (0.999 - 1.005) | 0.249 | 0.997 (0.996 - 0.997) | <0.001 ** | 1.002 (0.997 - 1.008) | 0.356 | 0.995 (0.994 - 0.997) | <0.001 ** |
| Ratios | NLR | 1.001 (1.000 - 1.003) | 0.152 | 1.001 (1.000 - 1.001) | 0.002 * | 1.003 (1.000 - 1.006) | 0.063 | 1.001 (1.000 - 1.001) | 0.190 |
|  | PLR | 1.000 (0.998 - 1.002) | 0.877 | 0.999 (0.999 - 1.000) | 0.008 * | 1.002 (0.998 - 1.005) | 0.360 | 0.999 (0.998 - 1.000) | 0.004 * |
|  | MLR | 1.001 (1.000 - 1.003) | 0.097 | 1.000 (1.000 - 1.001) | 0.157 | 1.002 (0.999 - 1.005) | 0.271 | 1.001 (1.000 - 1.001) | 0.096 |
|  | SII | 1.002 (1.001 - 1.003) | 0.006 * | 0.999 (0.999 - 1.000) | 0.001 * | 1.004 (1.001 - 1.007) | 0.009 * | 0.999 (0.998 - 1.000) | 0.004 * |
|  | MPVPR | 1.001 (0.998 - 1.004) | 0.580 | 1.002 (1.001 - 1.003) | <0.001 ** | 1.000 (0.995 - 1.005) | 0.962 | 1.003 (1.001 - 1.004) | <0.001 ** |
|  | MPVLR | 0.999 (0.997 - 1.002) | 0.588 | 1.001 (1.001 - 1.002) | <0.001 ** | 1.000 (0.996 - 1.004) | 0.966 | 1.001 (1.000 - 1.002) | 0.011 * |
|  | MPVMR | 0.999 (0.997 - 1.001) | 0.158 | 1.001 (1.000 - 1.001) | <0.001 ** | 0.998 (0.994 - 1.002) | 0.284 | 1.001 (1.000 - 1.002) | 0.106 |
|  | MPVNR | 0.998 (0.997 - 1.000) | 0.019 * | 1.001 (1.000 - 1.001) | 0.011 * | 0.996 (0.993 - 0.999) | 0.017 * | 1.001 (1.000 - 1.002) | 0.051 |

** This is p-value < 0.001.; * This is p-value < 0.05

# Figure S2. Temporal changes in all markers relative to the presence or absence of septic shock.

## a) WBC


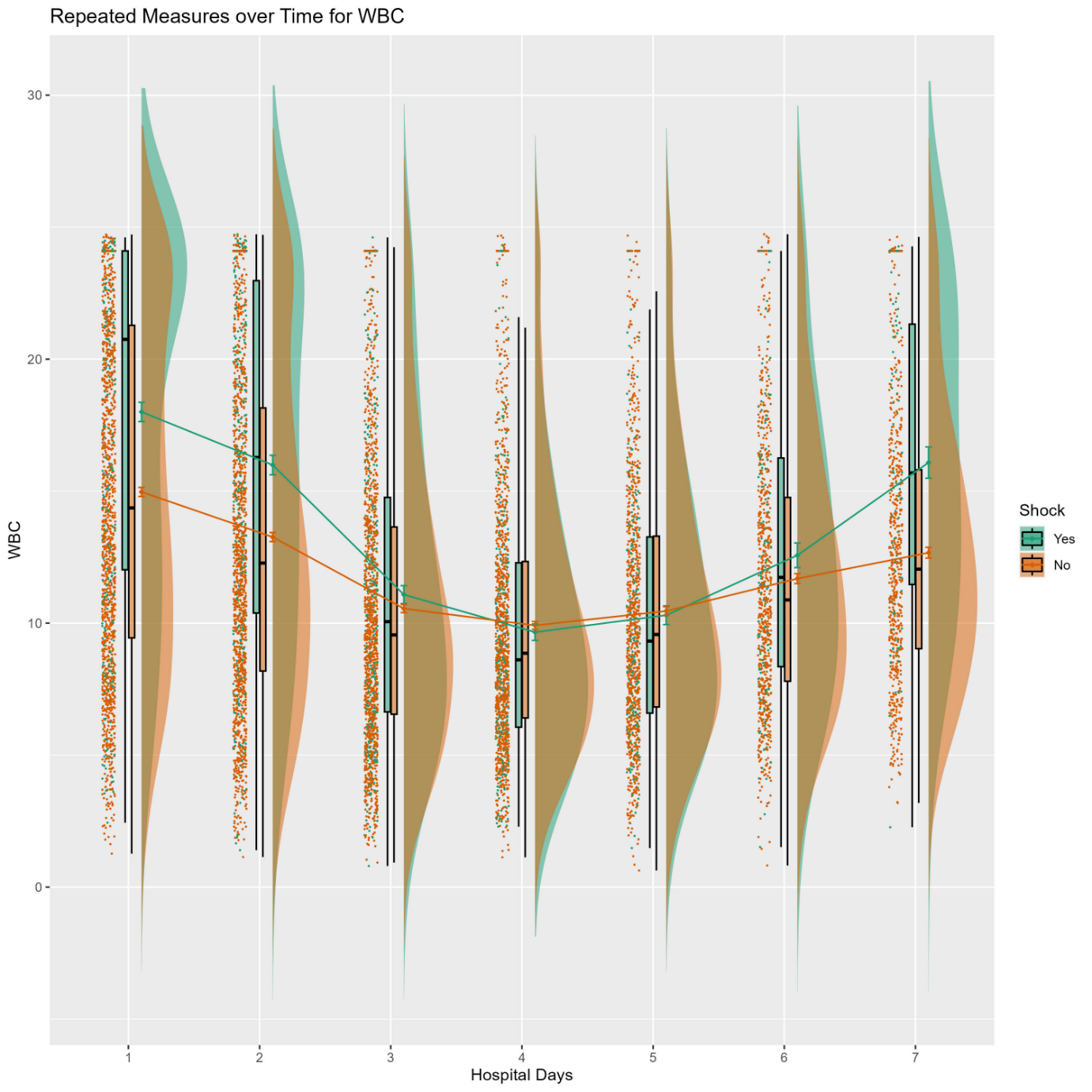


## b) Neutrophil


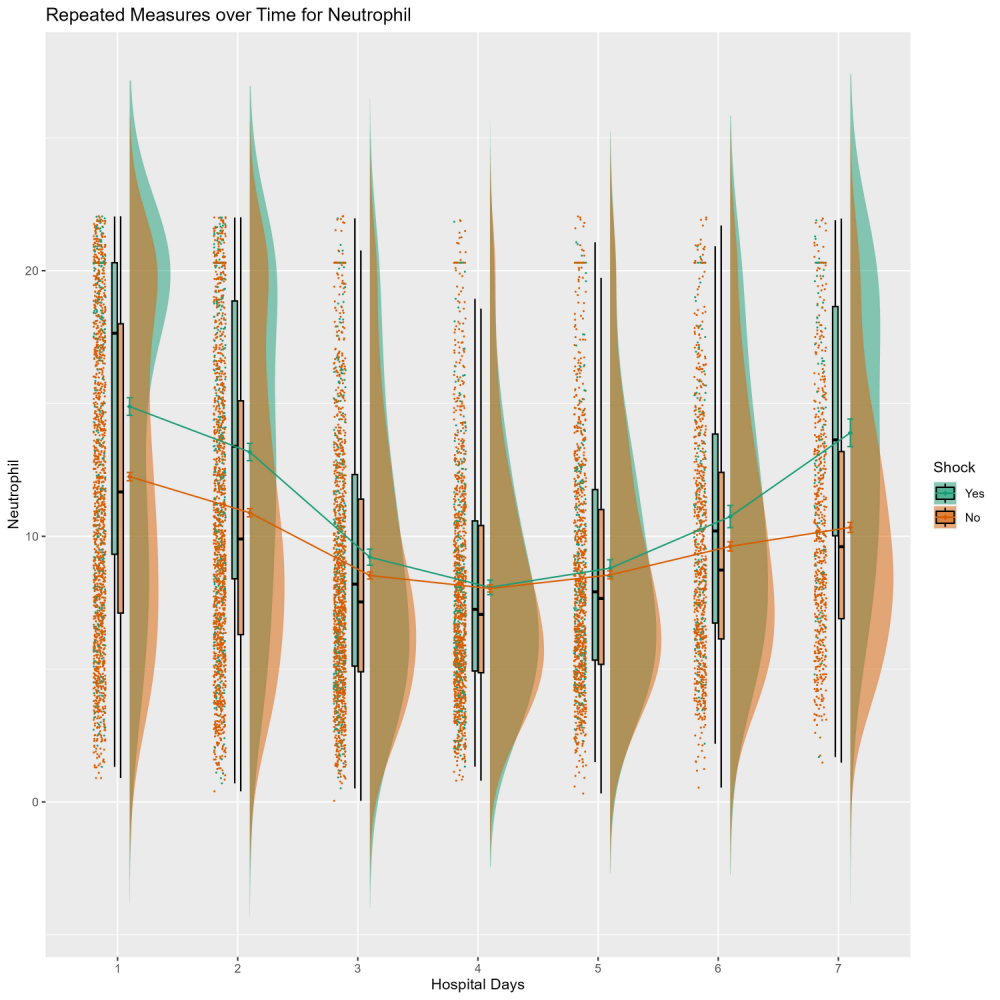


## c) Lymphocyte


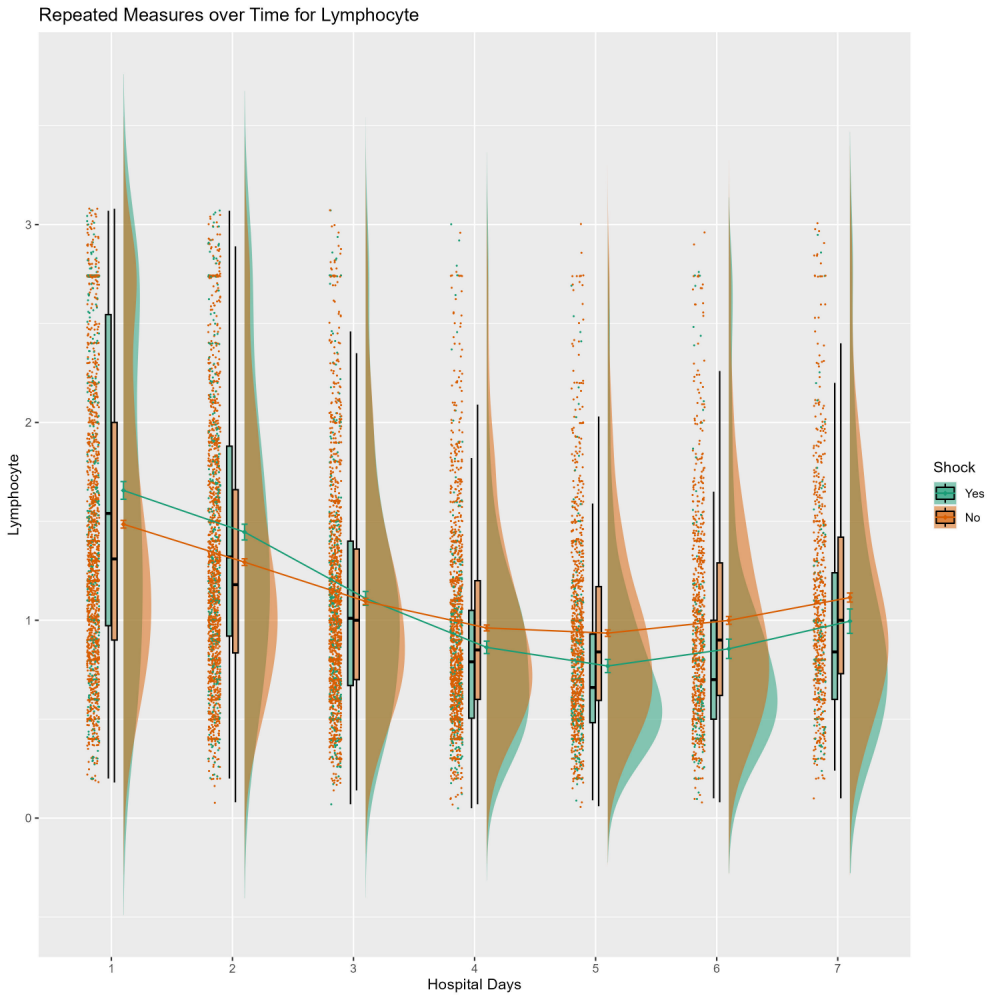


## d) Monocyte


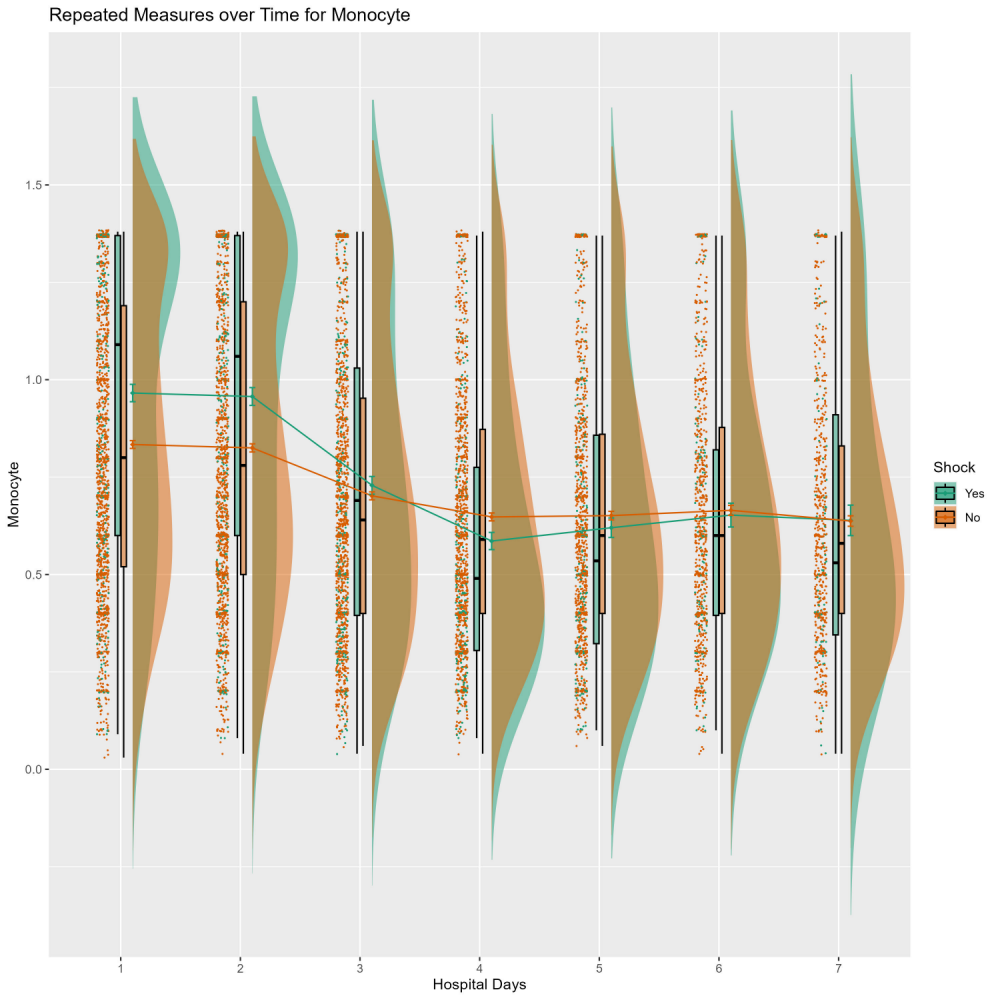


## e) Basophil


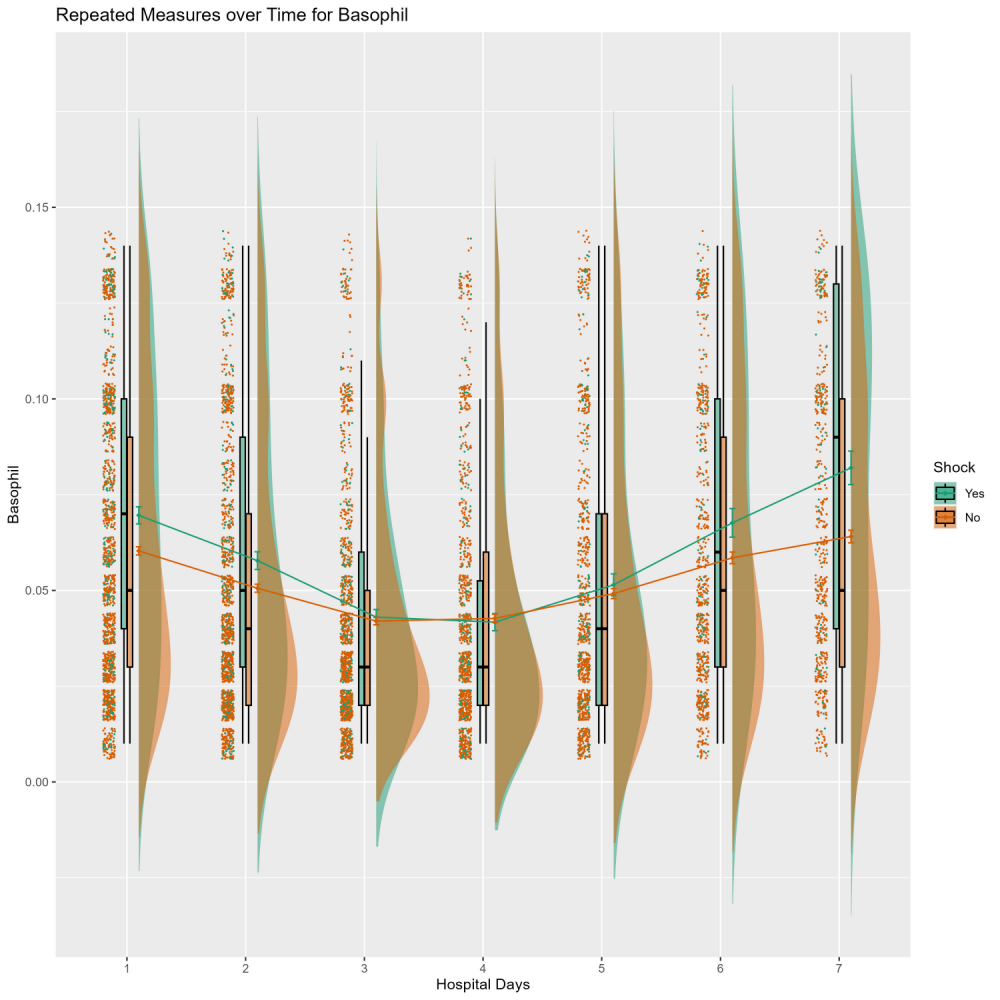


## f) Immature Granulocyte


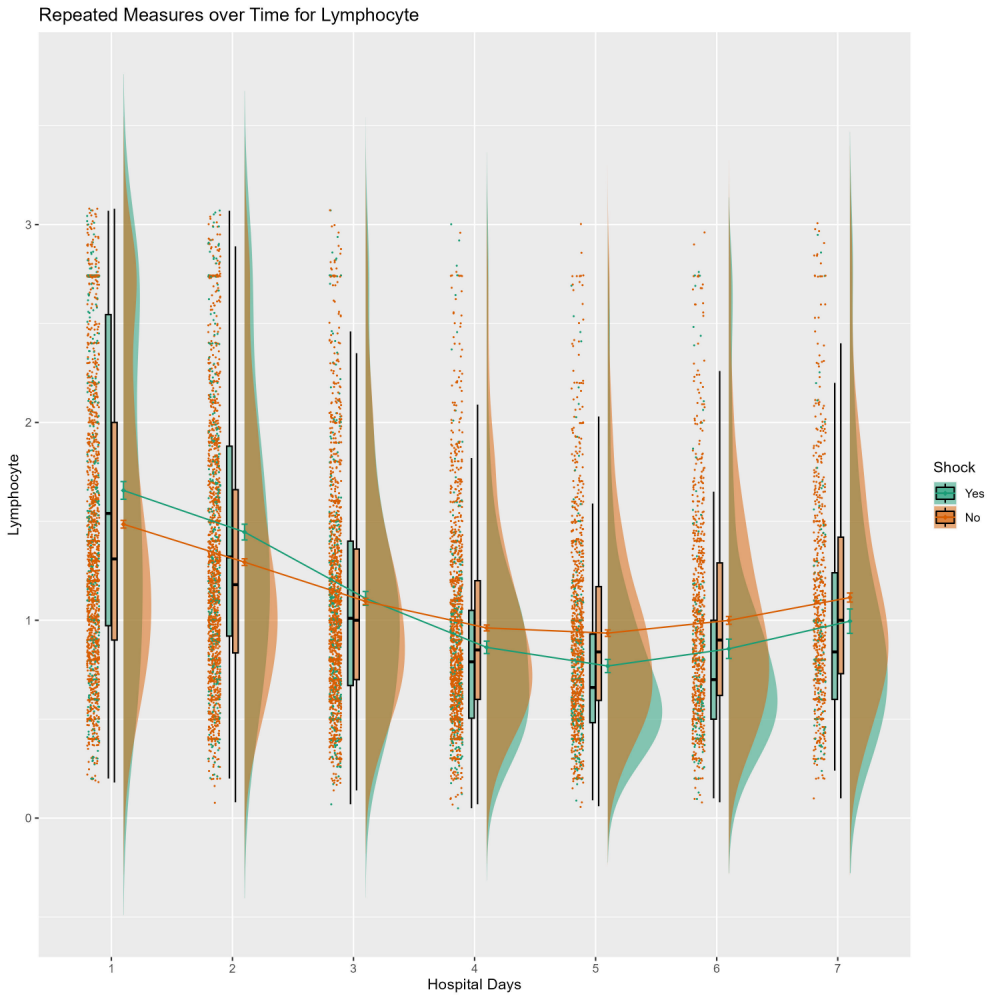


## g) RBC


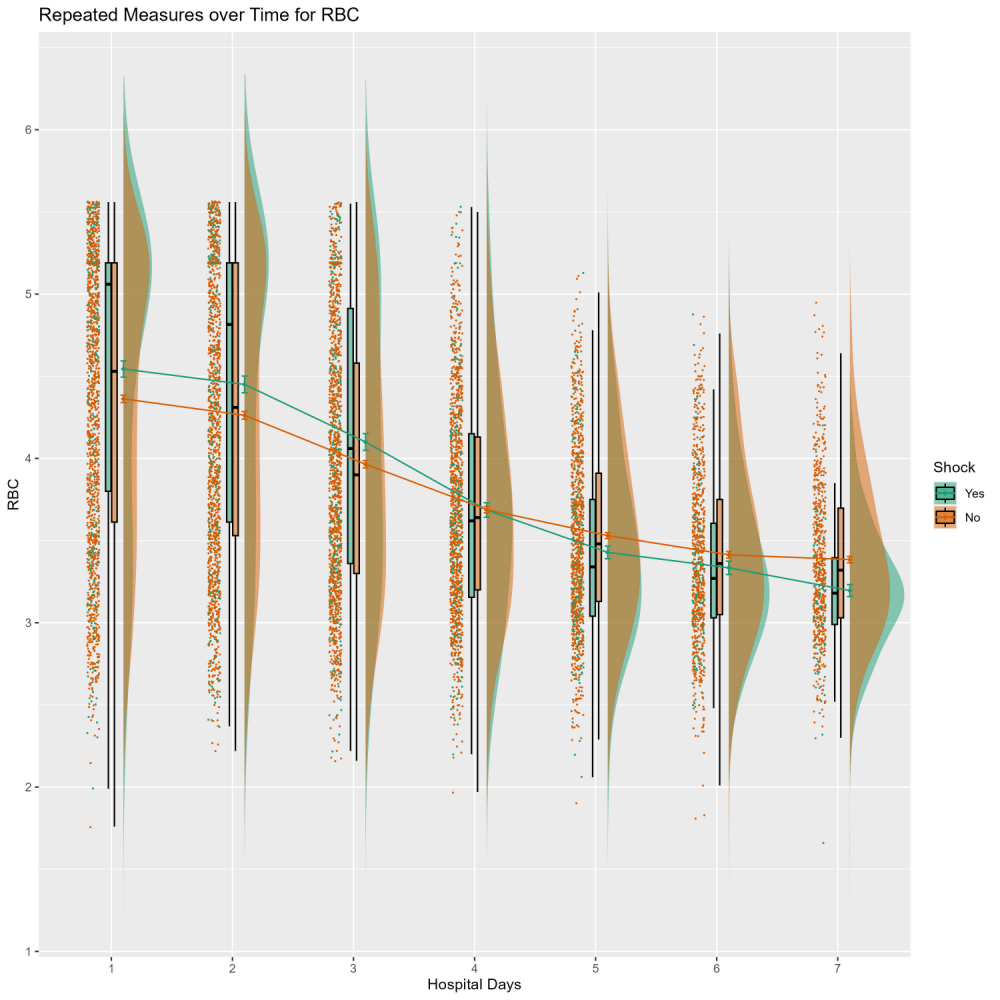


## h) RDW


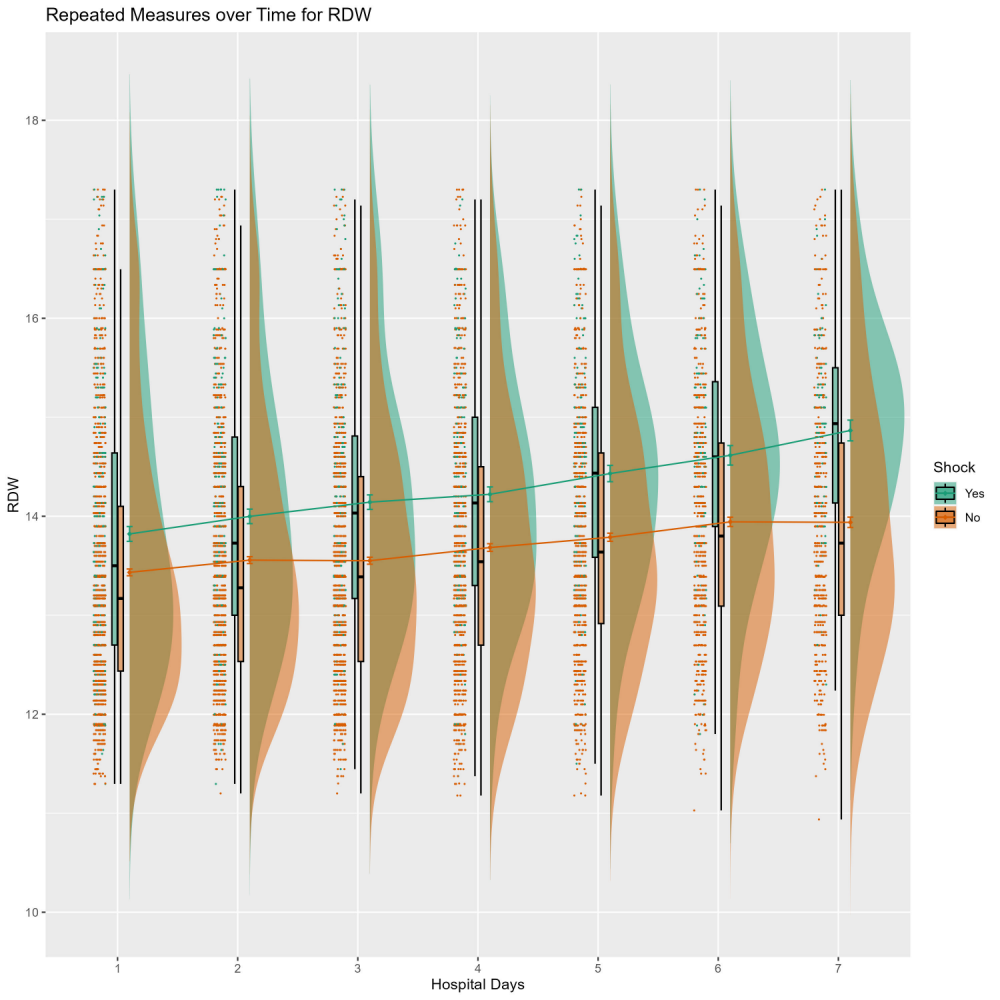


## i) Hct


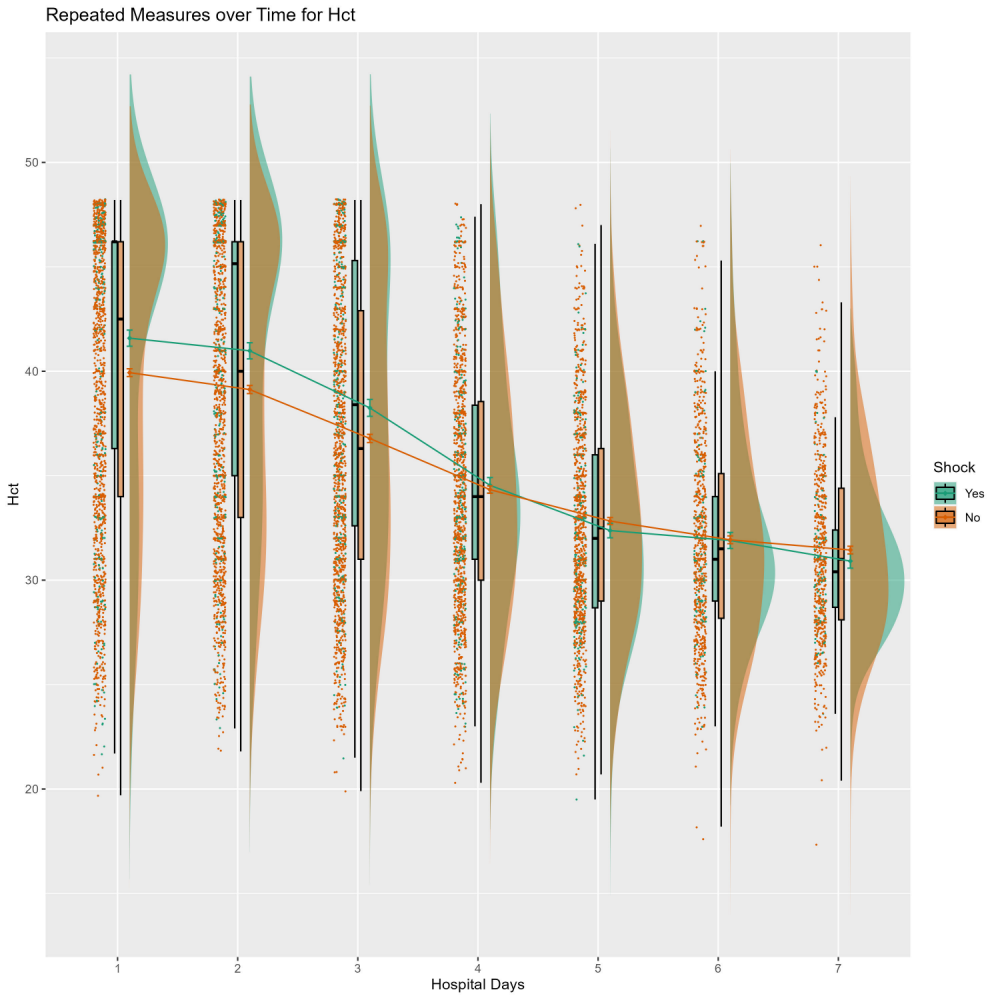


## j) Hb


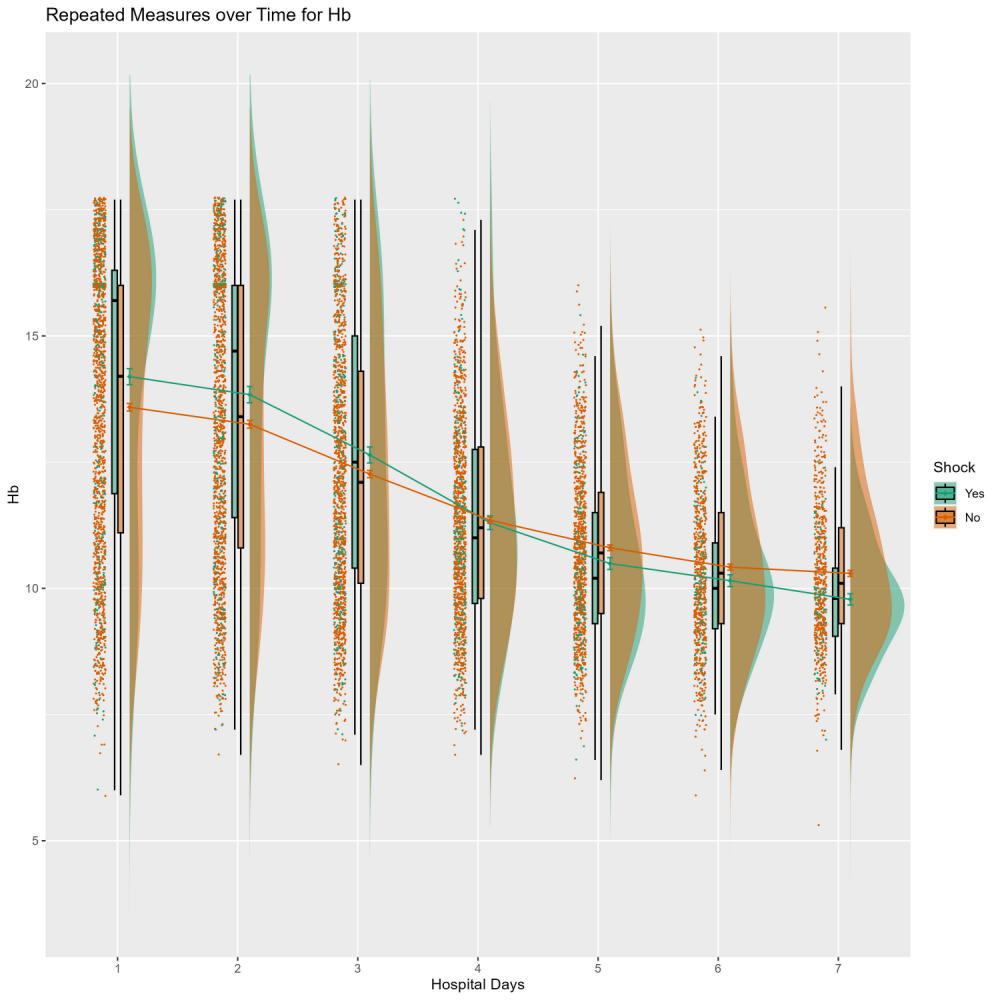


## k) MCV


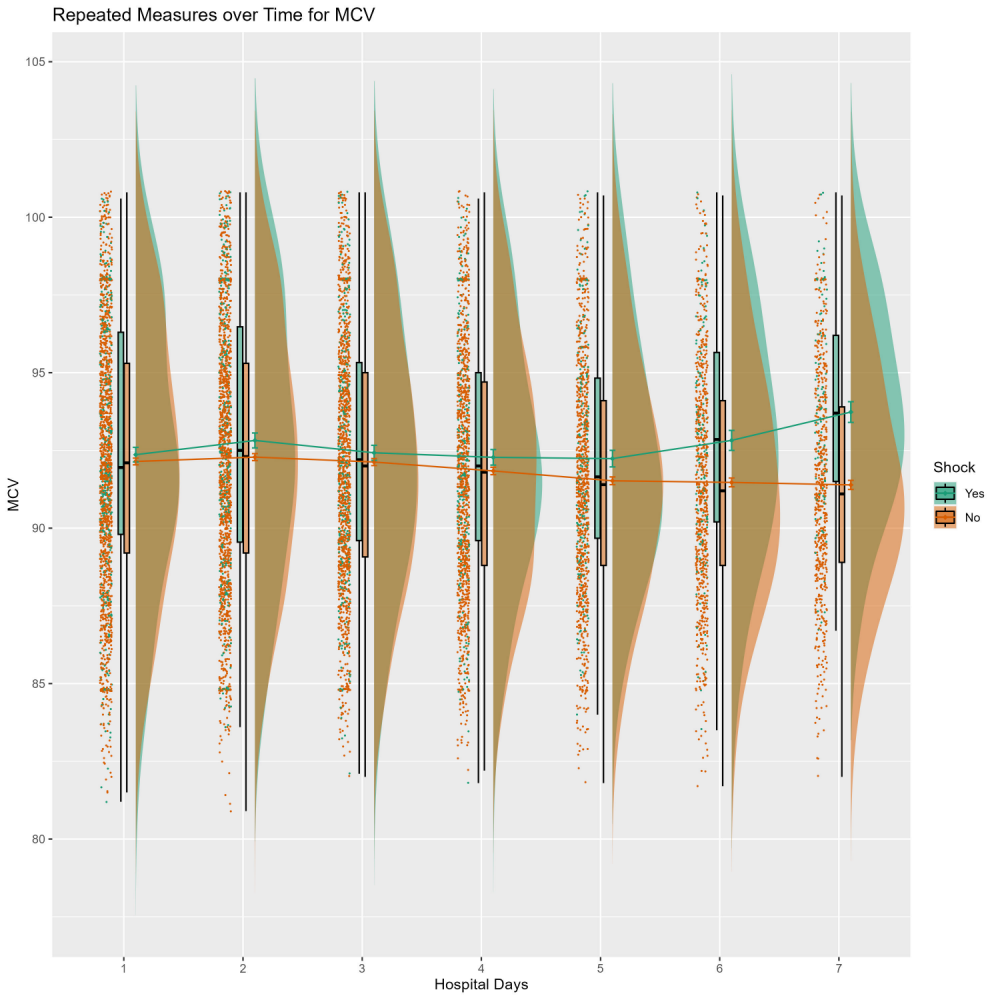


## l) MCH


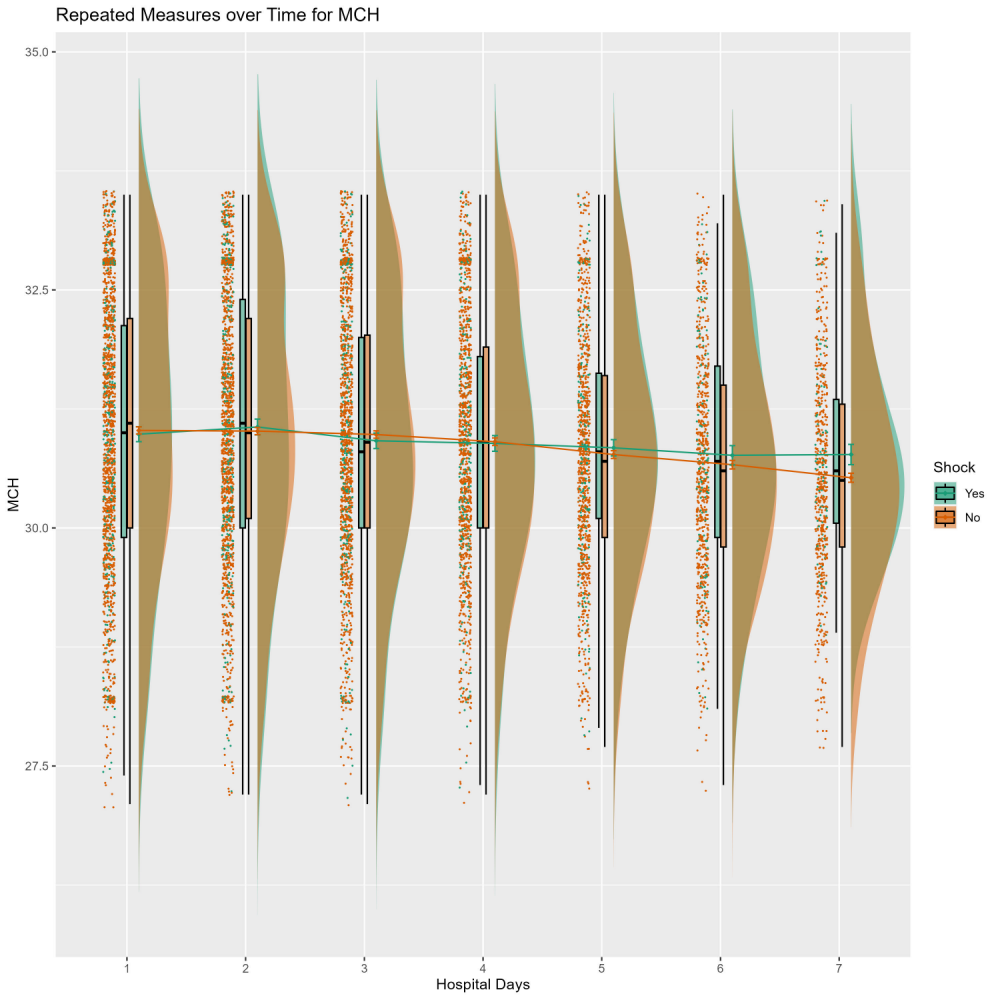


## m) MCHC


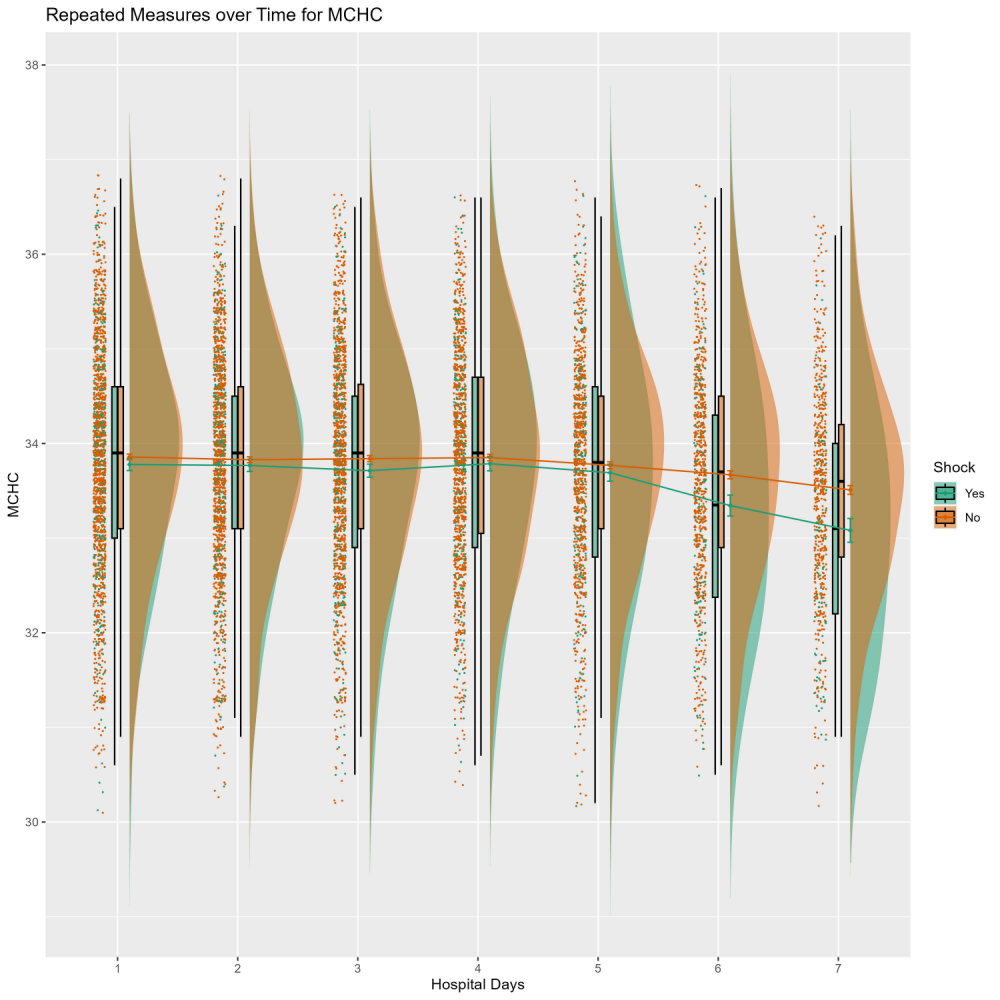


## n) Platelet


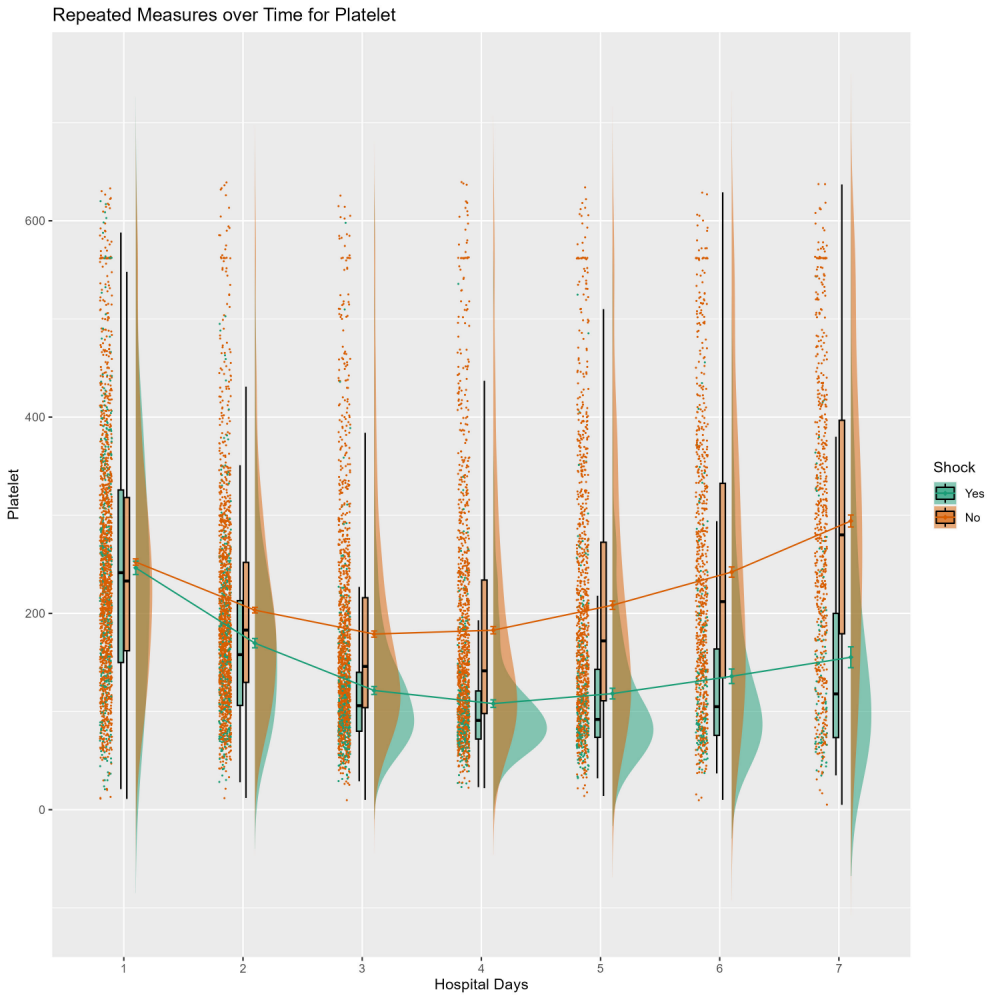


## o) MPV


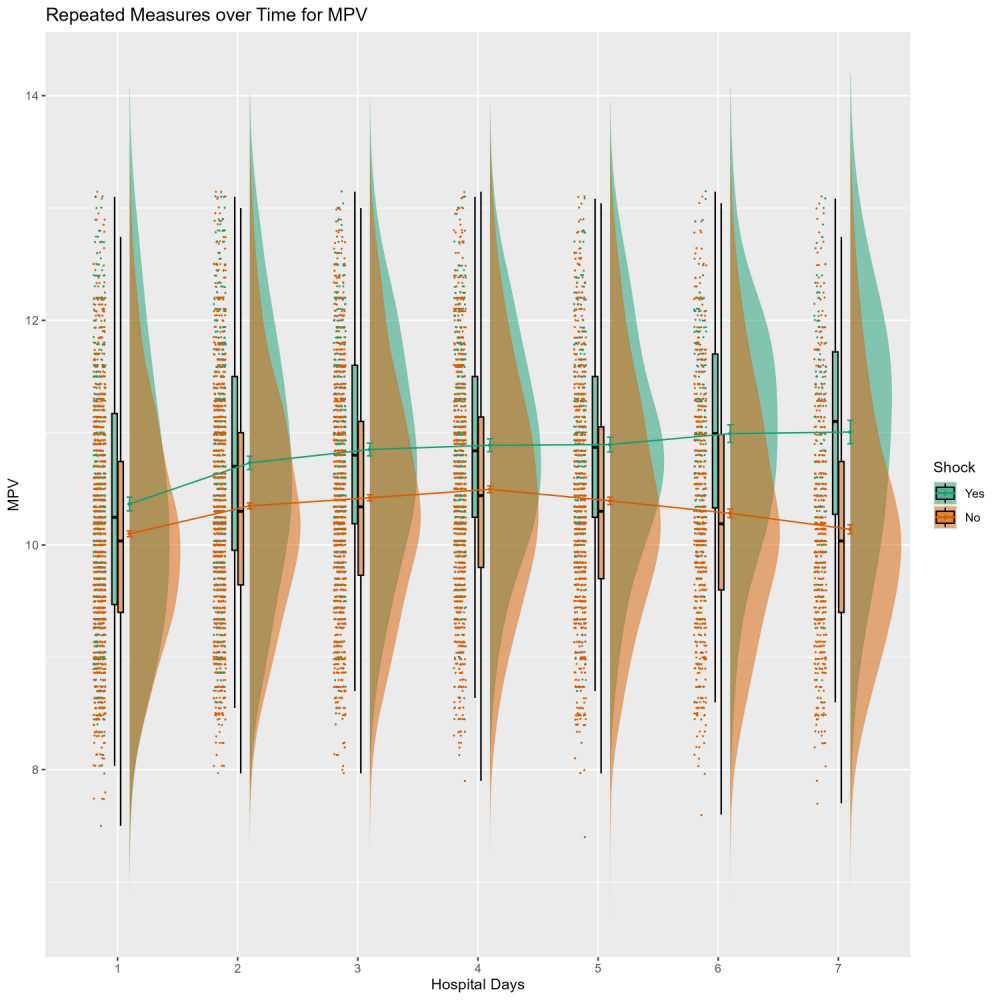


## p) PDW


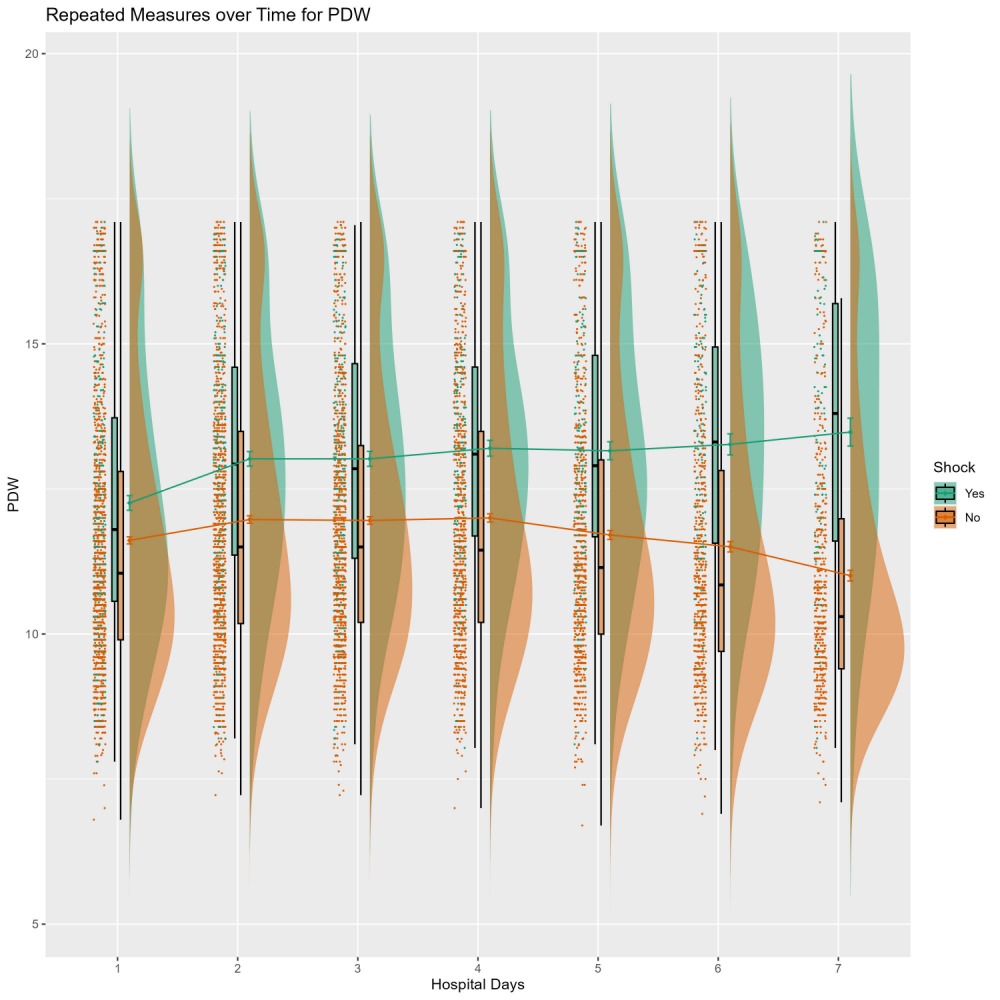


## q) PCT


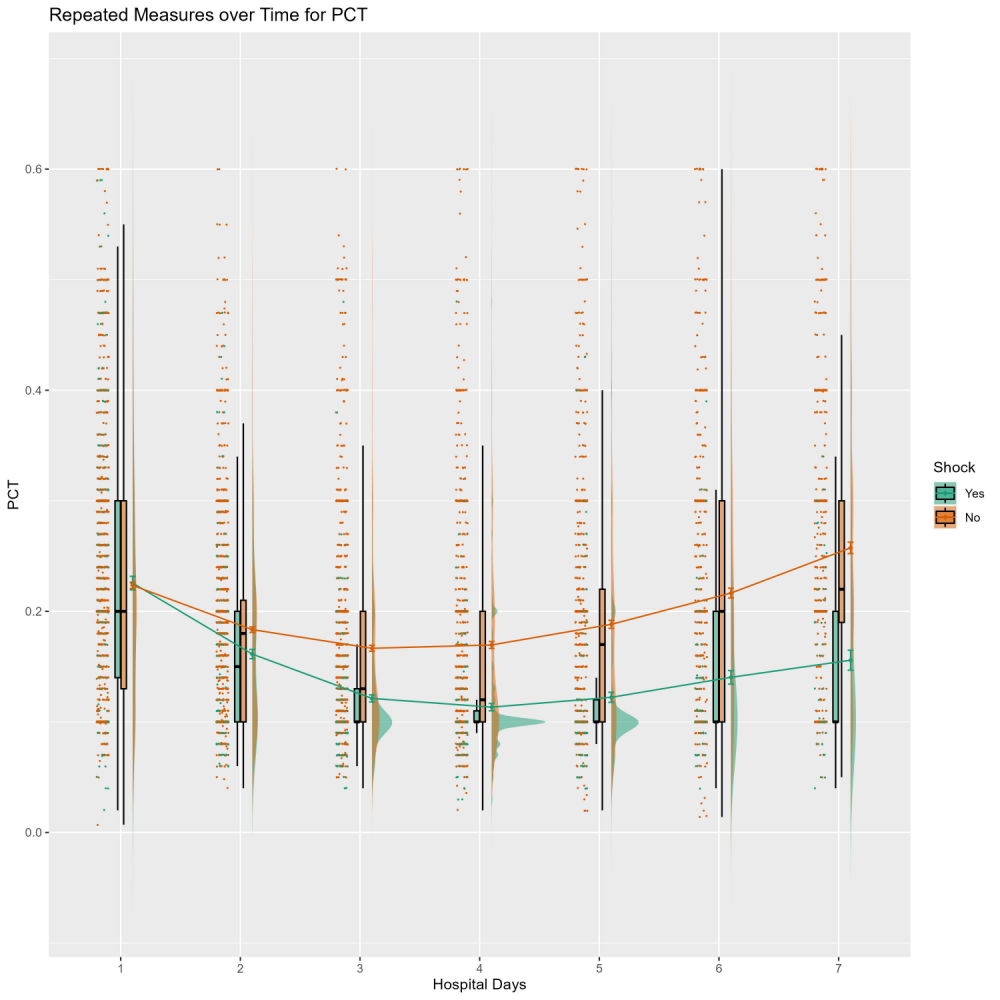


## r) NLR


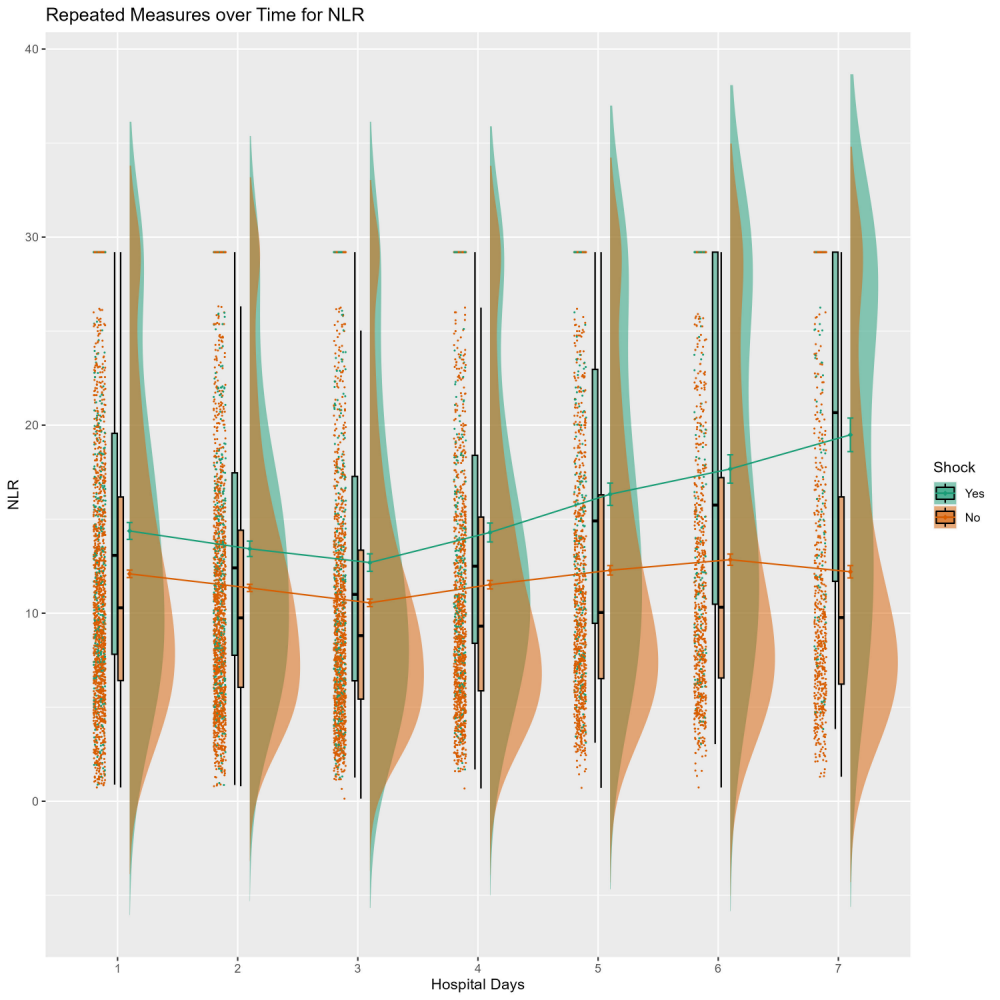


## s) PLR


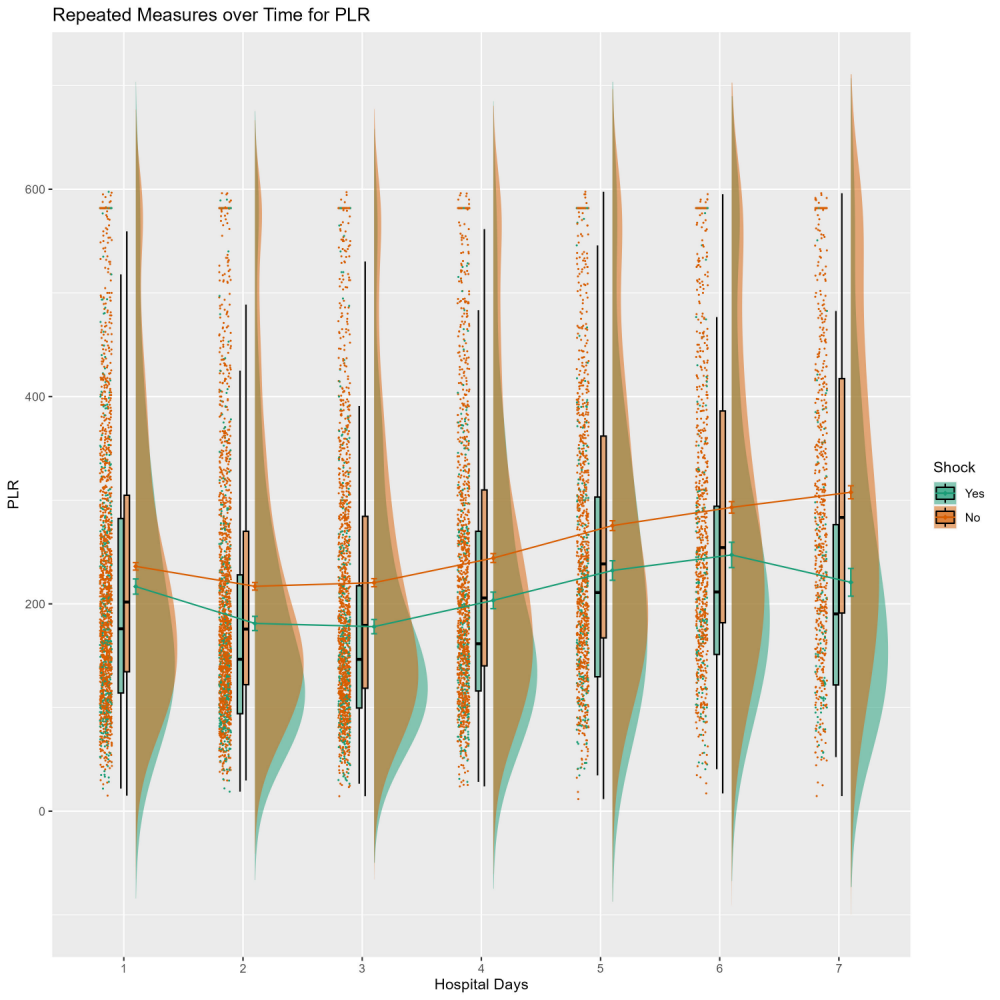


## t) MLR


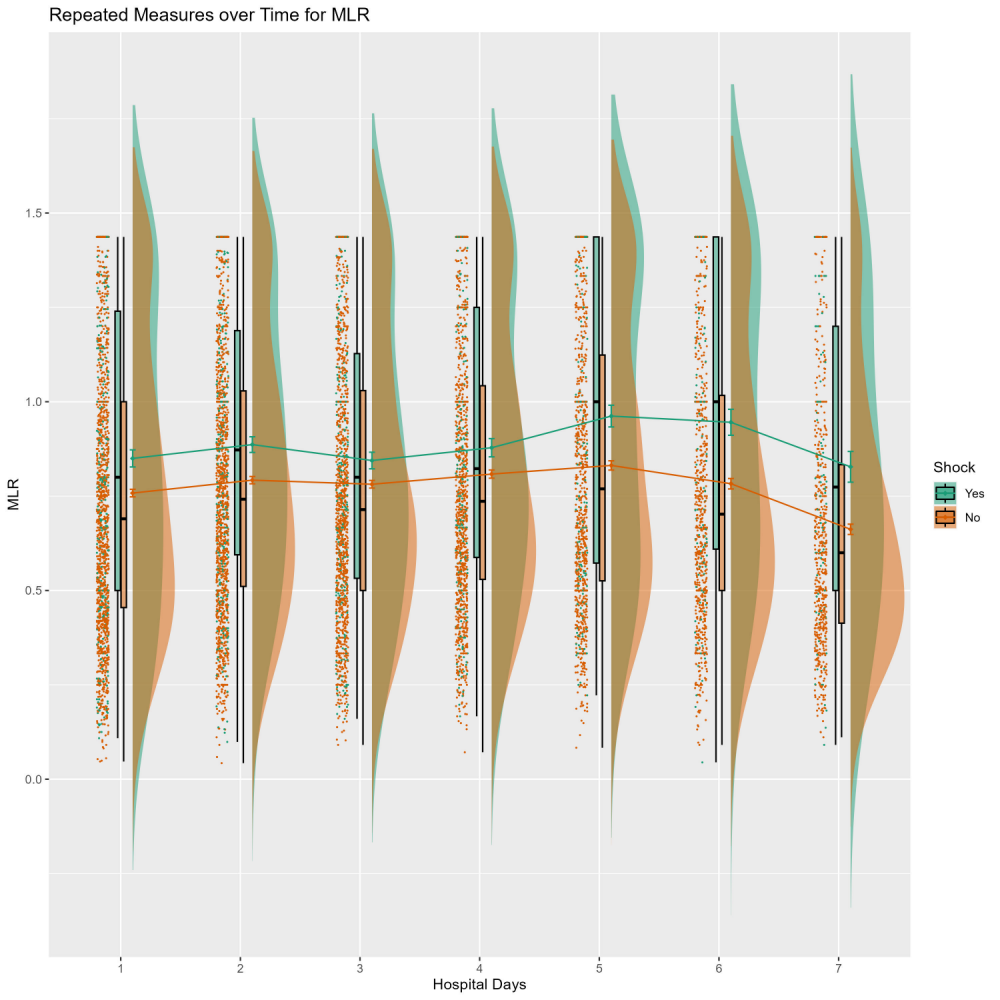


## u) SII


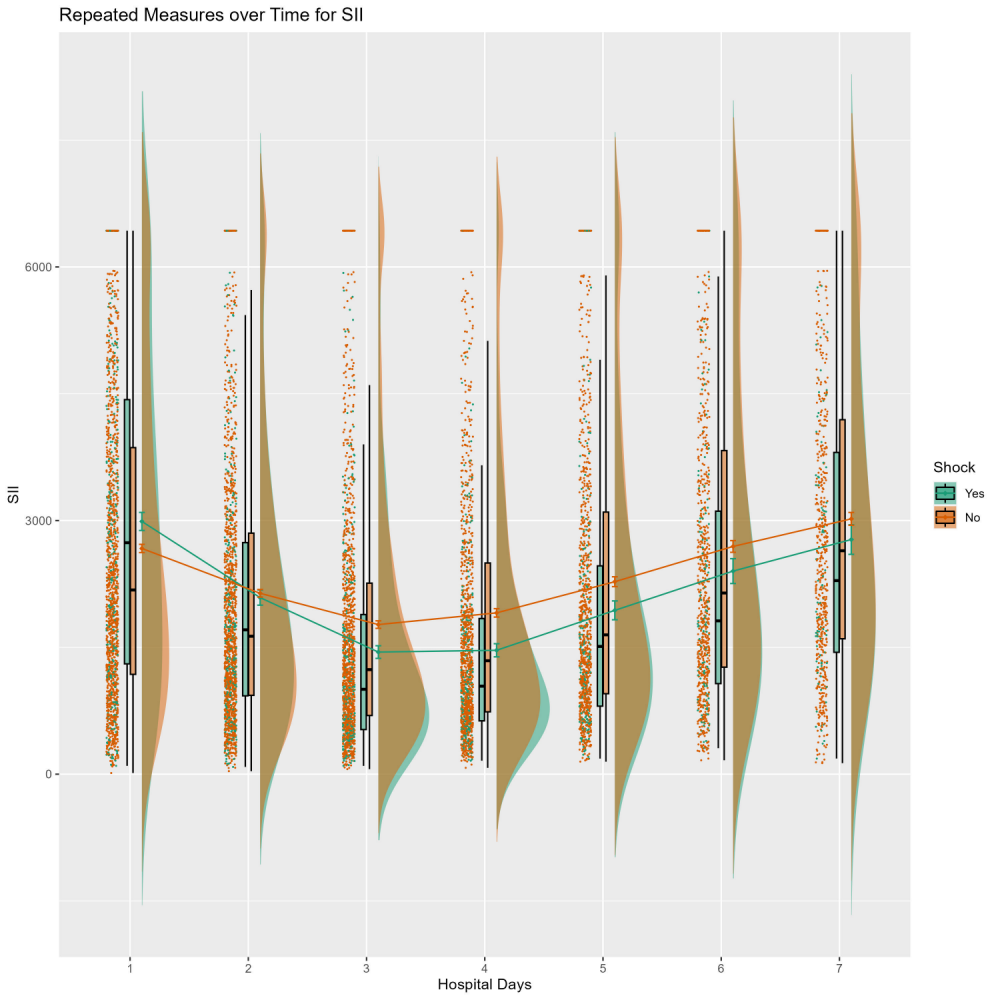


## v) MPVPR


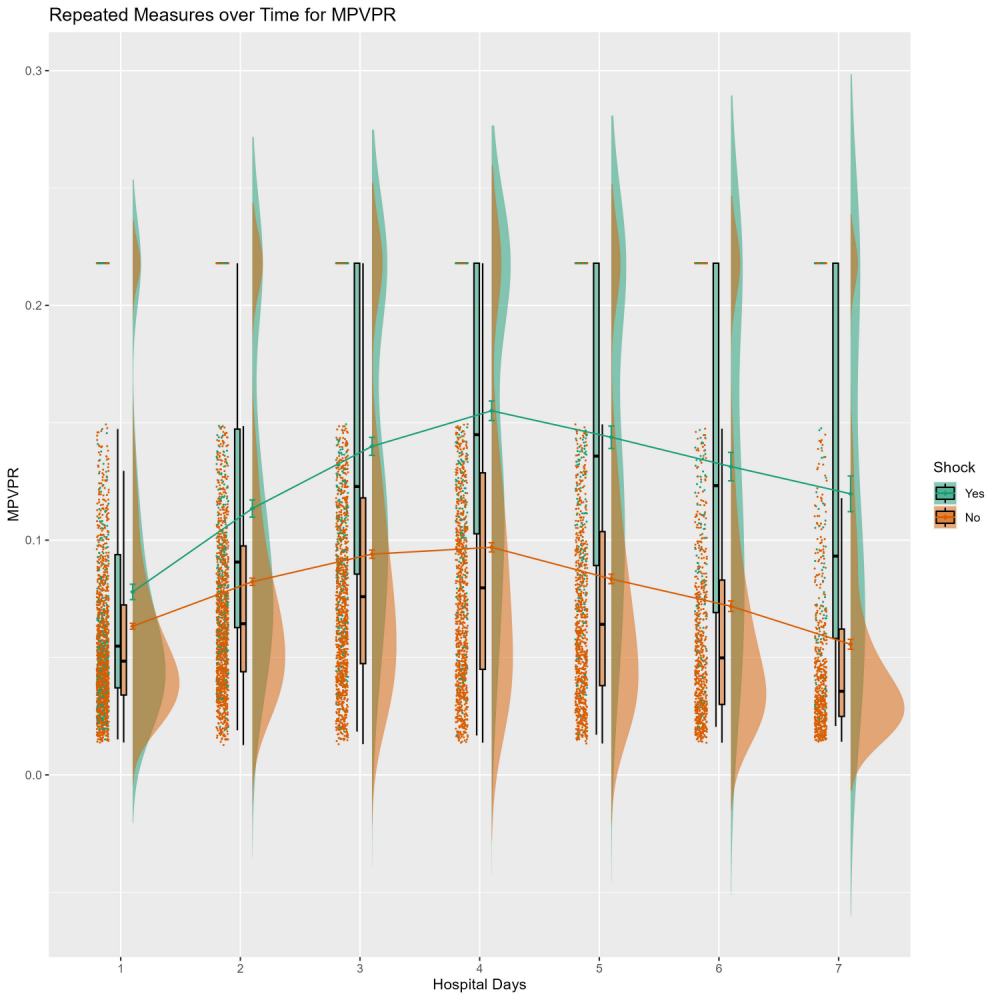


## x) MPVLR


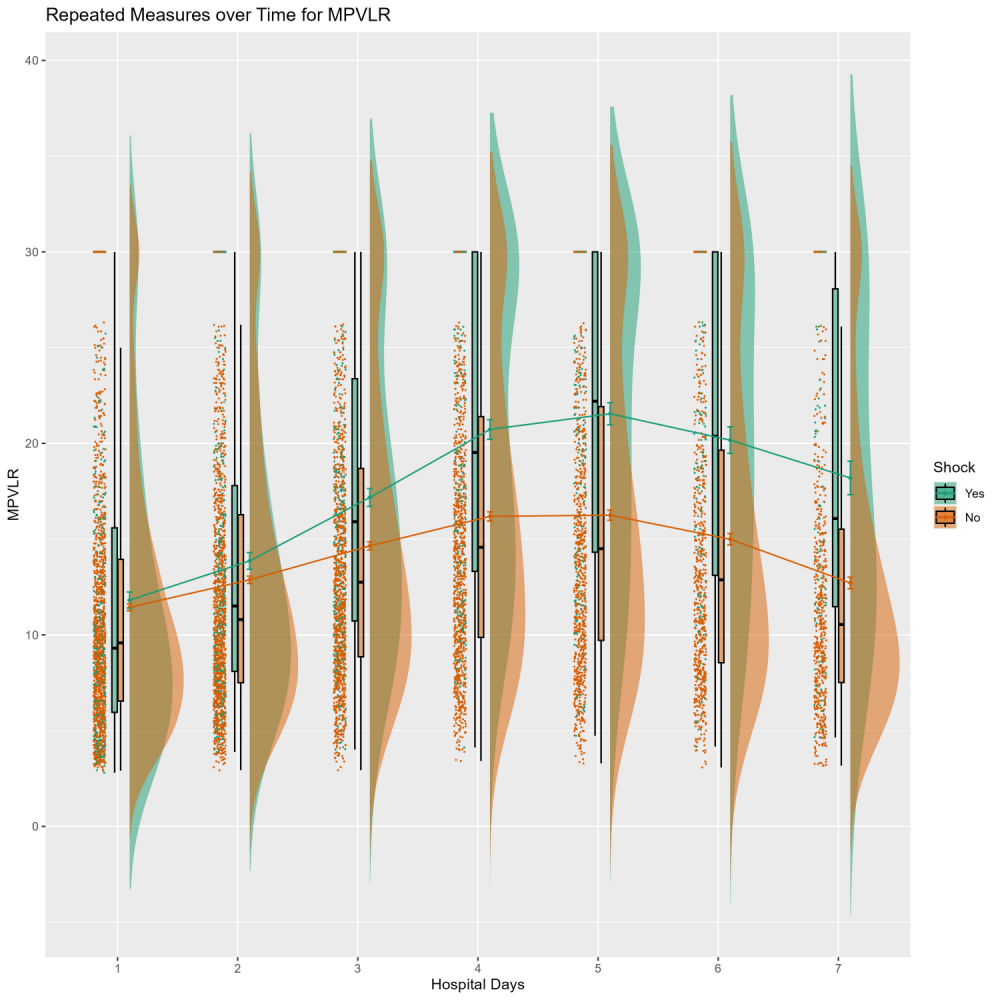


## y) MPVMR


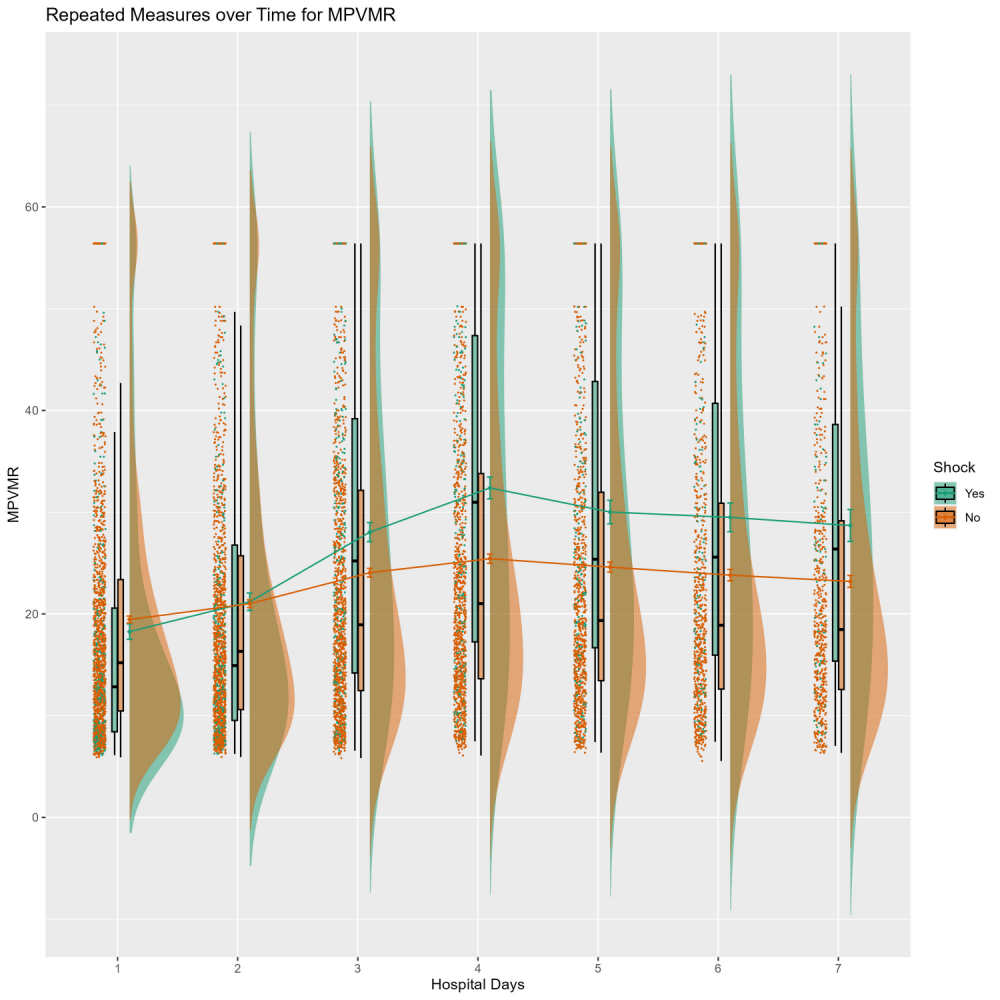


## z) MPVNR


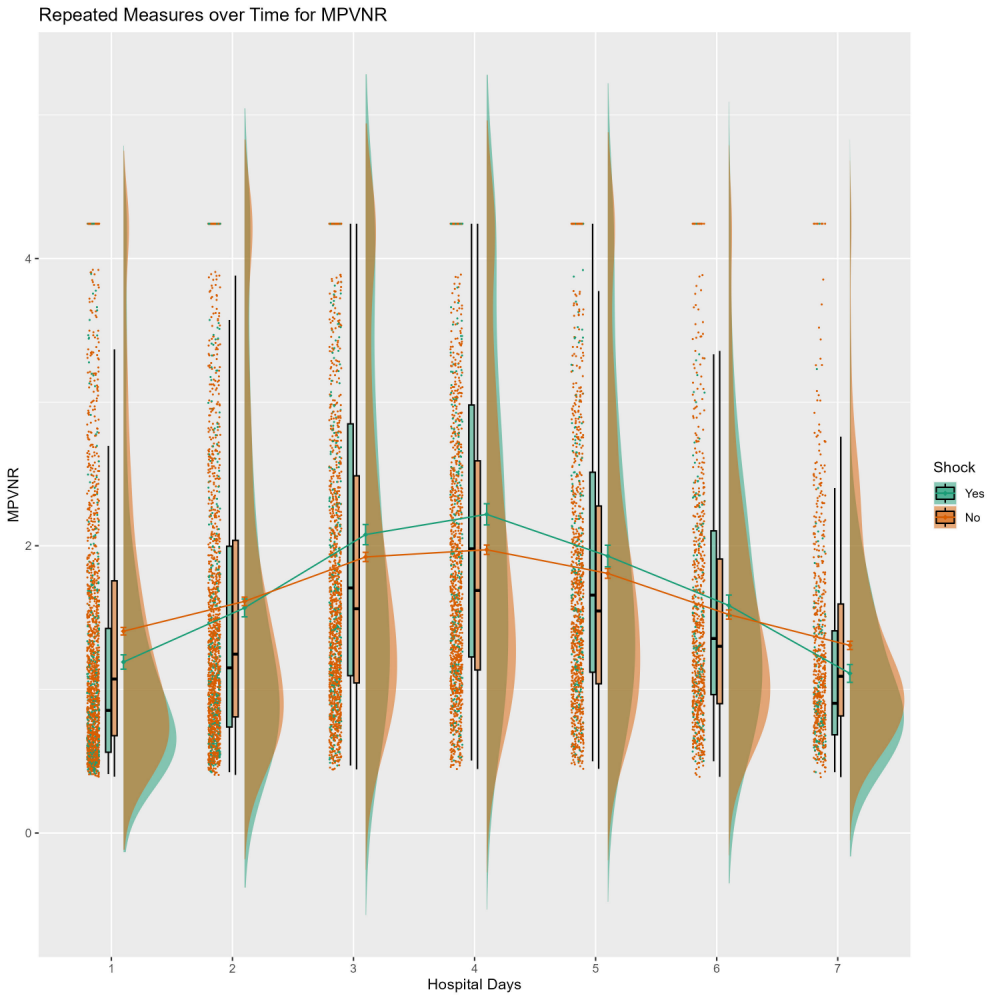


# Figure S3. Culture-Positivity Frequency during Sepsis Diagnosis


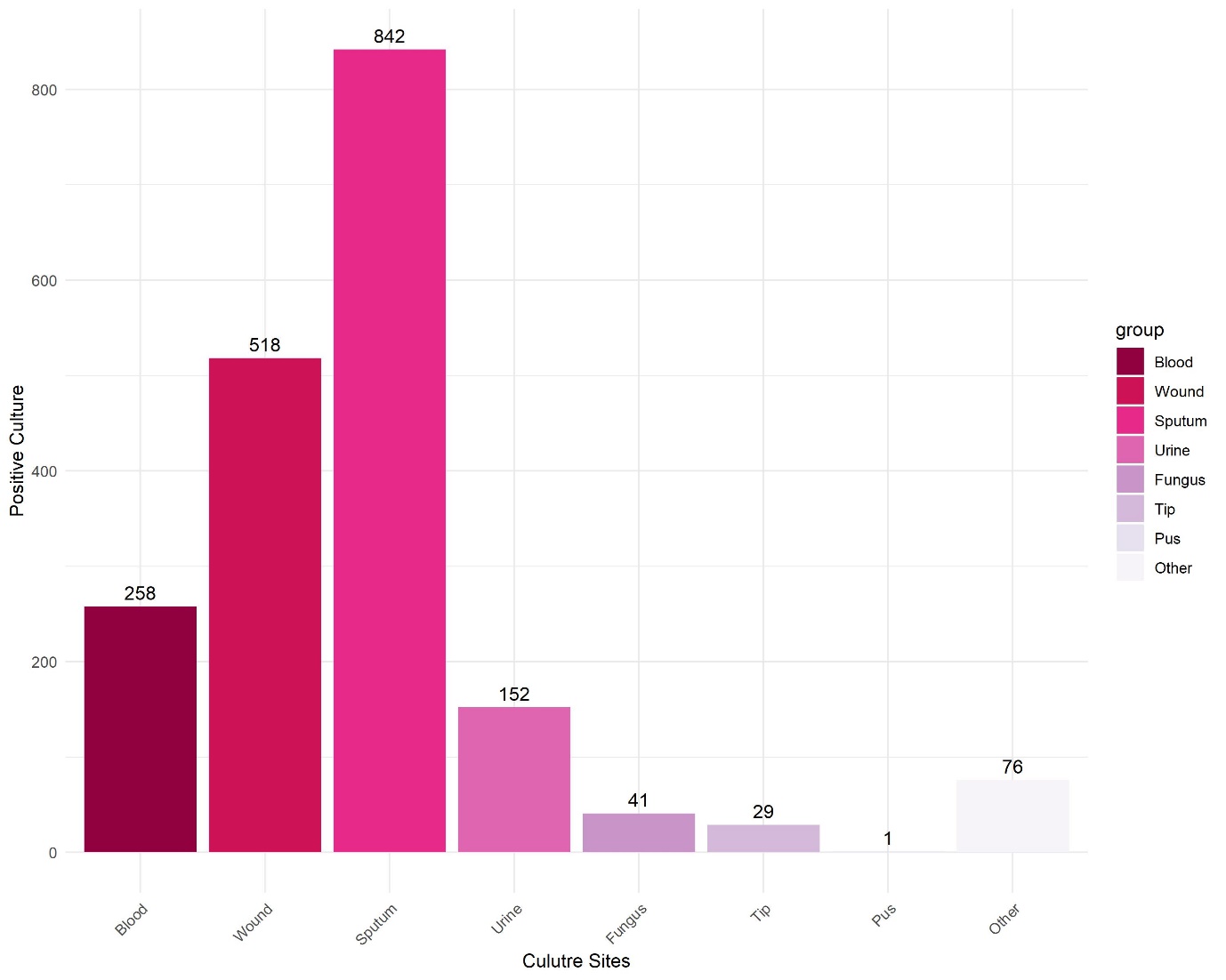

Supplement: Supplementary file 2 — Supplementary Information 2. [file 41598_2023_50695_MOESM2_ESM.docx]
